# β,β-Directly Linked Porphyrin Rings: Synthesis, Photophysical Properties, and Fullerene Binding

**DOI:** 10.1021/jacs.3c03549

**Published:** 2023-05-18

**Authors:** Qiang Chen, Amber L. Thompson, Kirsten E. Christensen, Peter N. Horton, Simon J. Coles, Harry L. Anderson

**Affiliations:** † Department of Chemistry, Chemistry Research Laboratory, University of Oxford, Oxford OX1 3TA, U.K.; ‡ National Crystallography Service, School of Chemistry, University of Southampton, Southampton SO17 1BJ, U.K.

## Abstract

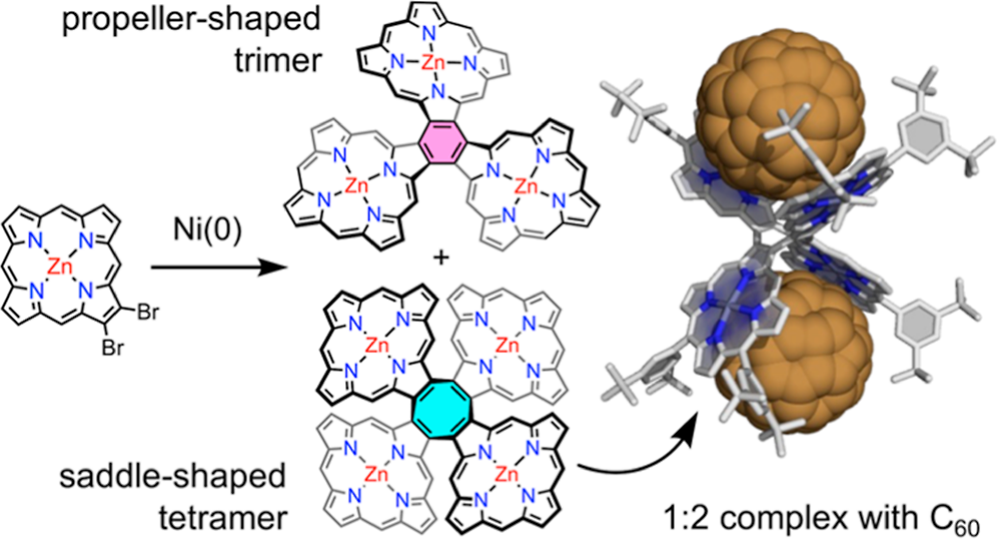

Cyclic porphyrin oligomers have been studied as models for photosynthetic light-harvesting antenna complexes and as potential receptors for supramolecular chemistry. Here, we report the synthesis of unprecedented β,β-directly linked cyclic zinc porphyrin oligomers, the trimer (**CP3**) and tetramer (**CP4**), by Yamamoto coupling of a 2,3-dibromoporphyrin precursor. Their three-dimensional structures were confirmed by nuclear magnetic resonance (NMR) spectroscopy, mass spectrometry, and single-crystal X-ray diffraction analyses. The minimum-energy geometries of **CP3** and **CP4** have propeller and saddle shapes, respectively, as calculated using density functional theory. Their different geometries result in distinct photophysical and electrochemical properties. The smaller dihedral angles between the porphyrin units in **CP3**, compared with **CP4**, result in stronger π-conjugation, splitting the ultraviolet–vis absorption bands and shifting them to longer wavelengths. Analysis of the crystallographic bond lengths indicates that the central benzene ring of the **CP3** is partially aromatic [harmonic oscillator model of aromaticity (HOMA) 0.52], whereas the central cyclooctatetraene ring of the **CP4** is non-aromatic (HOMA –0.02). The saddle-shaped structure of **CP4** makes it a ditopic receptor for fullerenes, with affinity constants of (1.1 ± 0.4) × 10^5^ M^–1^ for C_70_ and (2.2 ± 0.1) × 10^4^ M^–1^ for C_60_, respectively, in toluene solution at 298 K. The formation of a 1:2 complex with C_60_ is confirmed by NMR titration and single-crystal X-ray diffraction.

## Introduction

**Figure 1:**
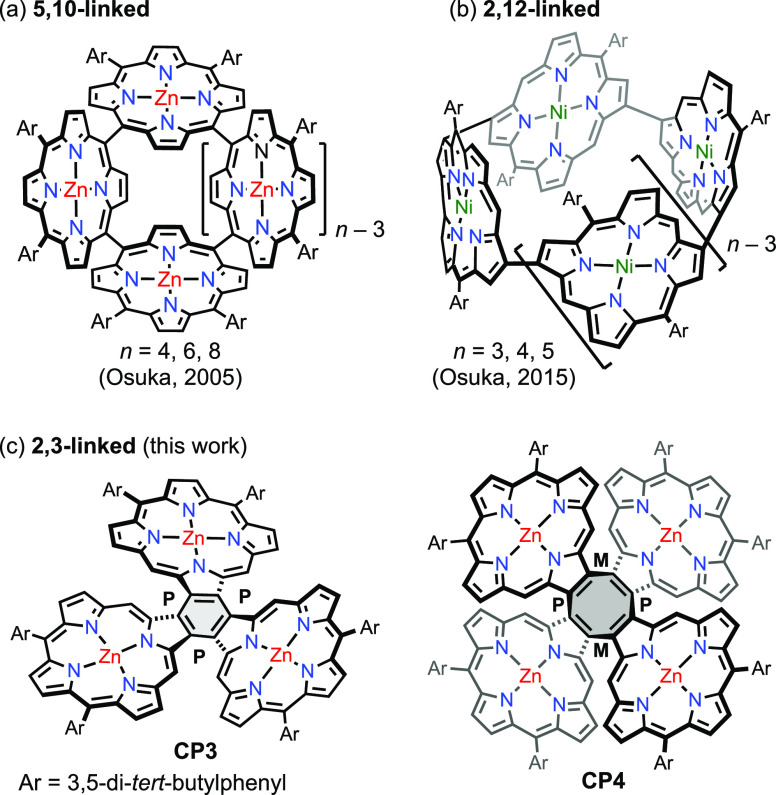
Representative examples of directly linked cyclic porphyrin oligomers: (a) *meso*,*meso* (5,10)-linked; (b) β,β (2,12)-linked; and (c) β,β (2,3)-linked trimer **CP3** and tetramer **CP4** in this work.

**Scheme 1:**
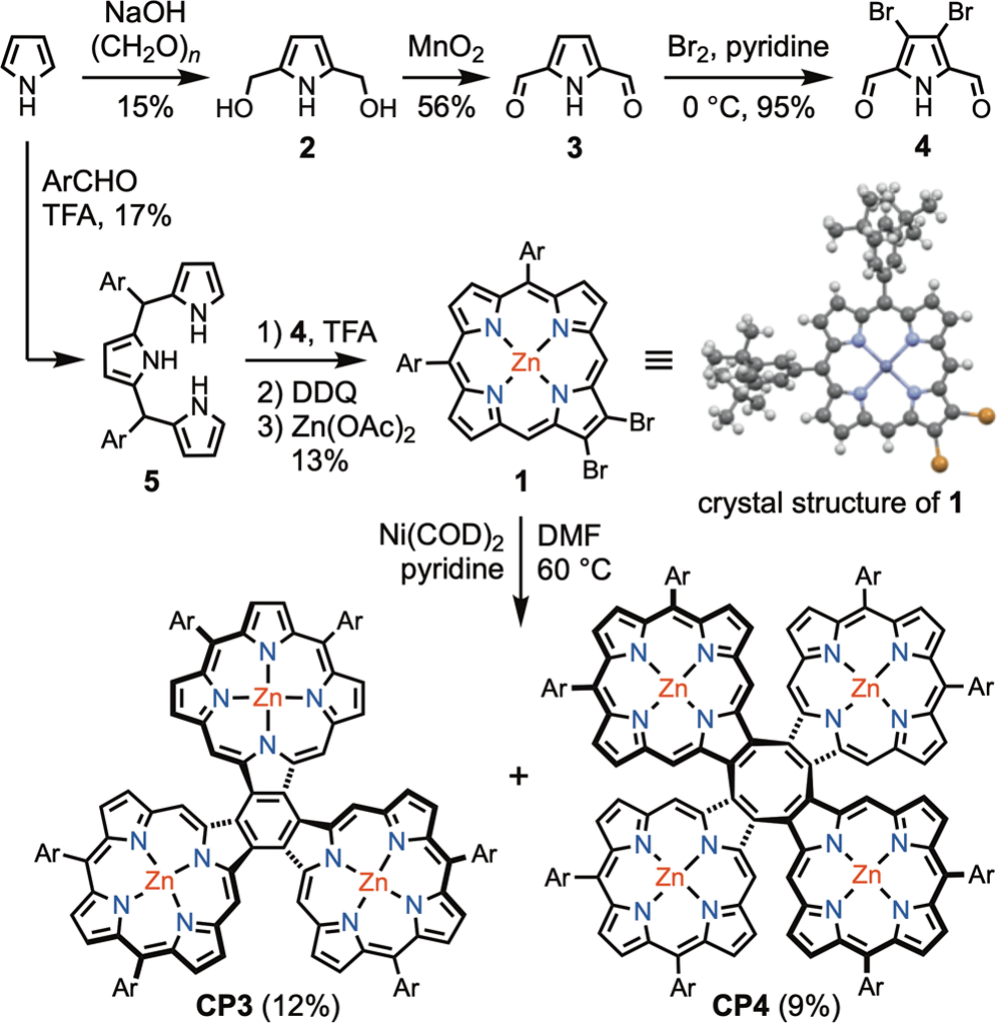
Synthesis of β,β-Linked Cyclic Porphyrin Trimer (**CP3**) and Tetramer (**CP4**) Ar = 3,5-di-*tert*-butylphenyl; COD = 1,5-cyclooctadiene; DDQ = 2,3-dichloro-5,6-dicyano-1,4-benzoquinone; TFA = CF_3_CO_2_H; and DMF = *N*,*N*-dimethylformamide.

Covalent cyclic porphyrin oligomers have been widely studied since the 1970s as models for photosynthetic systems,^1,2^ as multitopic receptors for molecule recognition,^3−6^ as catalysts,^7^ and as models for exploring electronic delocalization and aromaticity.^8^ Most of these macrocycles have “spacers” bridging between the porphyrin units, such as alkynes,^9^ butadiynes,^10^ thiophenes,^11^ or *para*-phenylenes.^12^ Directly linked cyclic porphyrin oligomers (i.e., with no spacers) can exhibit fascinating cooperative behavior as a result of the tight proximity between neighboring porphyrin chromophores,^13^ but they are difficult to synthesize. Osuka and co-workers reported the first *meso*,*meso* (5,10) directly linked cyclic porphyrin oligomers, with various ring sizes, through an oxidative coupling strategy^14^ (Figure 1a). The same team also prepared β,β (2,12)-linked oligomers^15^ (Figure 1b), via the formation of Pt^II^ complexes followed by reductive elimination, as well as β,β (3,7)-linked rings, via Suzuki–Miyaura coupling.^16^ These porphyrin arrays show unusual photophysical properties, such as size-dependent excitation energy transfer.^2,13,15^ Here, we report the synthesis of two unprecedented β,β (2,3) directly linked cyclic porphyrin oligomers (Figure 1c), trimer **CP3** and tetramer **CP4**, via one-step Yamamoto coupling of a 2,3-dibromo-10,15-bis-(3,5-di-*tert*-butylphenyl)porphyrin(Zn) **1** (Figure 1c and Scheme 1). These two porphyrin oligomers feature a central aromatic benzene or nonaromatic cyclooctatetraene (COT) ring, respectively. Their structures have been unambiguously characterized by a combination of nuclear magnetic resonance (NMR) spectroscopy, mass spectrometry, and X-ray single-crystal diffraction analysis.

The difference in geometry between **CP3** and **CP4** has a striking effect on their optical properties, electronic structures, and affinities for fullerenes. Trimer **CP3** is almost flat; its density functional theory (DFT)-calculated geometry is *D*_3_ propeller-shaped (Figure 1c), with a small energy difference of 4.6 kJ/mol with respect to other conformers, making it conformationally dynamic. In contrast, **CP4** has a saddle-shaped conformation with a high barrier to saddle inversion. The almost planar geometry of **CP3** allows efficient π-conjugation, reducing the highest occupied molecular orbital (HOMO)–lowest unoccupied molecular orbital (LUMO) gap and shifting the UV–vis absorption and fluorescence spectra to longer wavelengths, whereas absorption and fluorescence spectra of **CP4** are more like those of a porphyrin monomer, with weak electronic coupling between the porphyrin units. While **CP3** shows no interaction with fullerenes, the saddle-shaped geometry of **CP4** makes it a good ditopic receptor, with association constants of (1.1 ± 0.4) × 10^5^ M^–1^ for C_70_ and (2.2 ± 0.1) × 10^4^ M^–1^ for C_60_ in toluene solution at 298 K. This tetramer is the first directly linked cyclic porphyrin oligomer with a high affinity for fullerenes.

## Results and Discussion

### Synthesis and NMR Spectroscopy

The key intermediate in the synthesis of **CP3** and **CP4** is the 2,3-dibromo-10,15-diarylporphyrin **1**. This monomer was synthesized via [3 + 1] condensation of tripyrane **5** and 3,4-dibromopyrrole **4** (Scheme 1). First, twofold hydroxymethylation of pyrrole with paraformaldehyde gave 2,5-bis(hydroxymethyl)pyrrole **2** in 15% yield.^17^ Then, oxidation of **2** with activated MnO_2_ gave 2,5-diformylpyrrole **3** in 56% yield.^18^ Treatment with bromine in the presence of pyridine converts **3** to 3,4-dibromo-2,5-diformylpyrrole (**4**) in 95% yield.^19^ Meanwhile, tripyrrane **5** was synthesized in 17% yield by condensation of pyrrole and 3,5-di-*tert*-butylbenzaldehyde catalyzed by trifluoroacetic acid.^20^ Condensation of tripyrrane **5** and 3,4-dibromo-2,5-diformylpyrrole (**4**), followed by oxidation with DDQ and metalation with zinc acetate, provided porphyrin **1** in 13% yield over two steps.^21^ The structure of this dibromoporphyrin was confirmed by X-ray crystallography (Scheme 1, CCDC: 2253581). Finally, Yamamoto coupling of **1** using Ni(COD)_2_ in DMF gave a mixture of linear and cyclic porphyrin oligomers. After silica gel column chromatography, **CP3** and **CP4** were isolated in 12 and 9% yields, respectively. Molecular ions were observed at *m*/*z*: 2238.92 for **CP3** (calcd for C_144_H_150_N_12_Zn_3_, 2239.00 [M]^+^) and *m*/*z*: 2985.88 for **CP4** (calcd for C_192_H_200_N_16_Zn_4_, 2985.33 [M]^+^) by matrix-assisted laser desorption ionization time-of-flight mass spectrometry (MALDI-TOF MS) and the isotopic distribution patterns agree well with simulations (Figures S52 and S53). We also detected larger oligomeric byproducts by MS (Figure S48), but they were not isolated or fully characterized.

**Figure 2:**
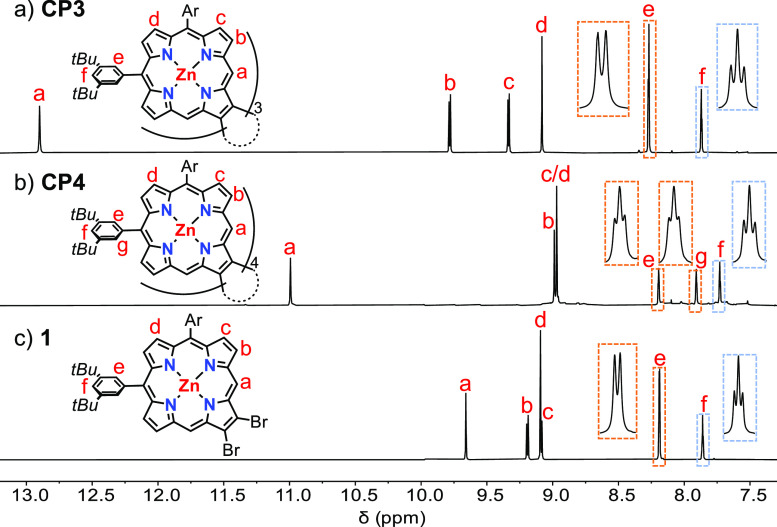
Aromatic regions of the ^1^H NMR spectra of (a) **CP3**, (b) **CP4** and (c) 2,3-dibromo-10,15-bis-(3,5-di-*tert*-butylphenyl)porphyrin(Zn) (**1**) recorded in CDCl_3_ (1% v/v of pyridine-*d*_5_ was added for **CP3** to suppress aggregation, see Figure S47 for spectra without pyridine-*d*_5_) at 298 K, 400 MHz.

**Figure 3:**
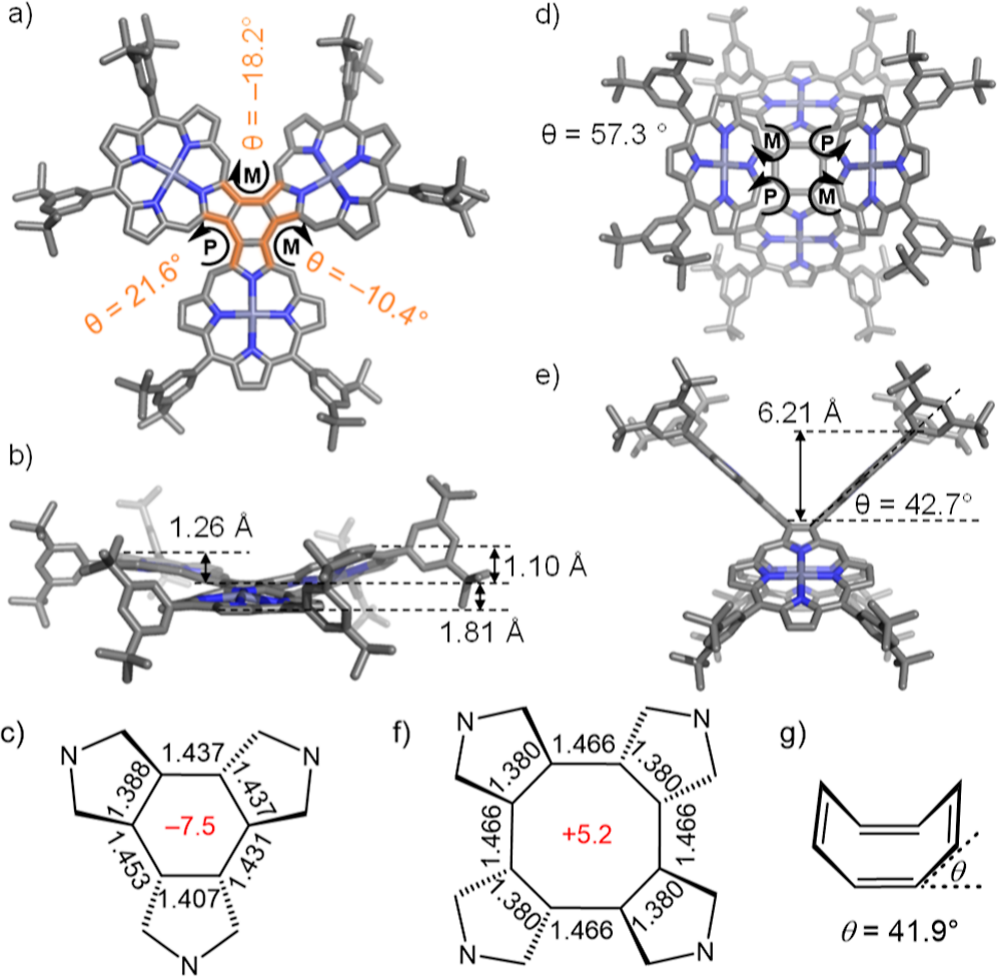
Structure of **CP3** from single-crystal X-ray diffraction studies (only one enantiomer is shown, hydrogen atoms and solvent molecules are omitted for clarity): (a) top view; (b) front view; (c) bond lengths in the central benzene ring and NICS(1) (in red); (d,e) optimized structure of **CP4** by DFT on the B3LYP/6-31G(d,p) level of theory; (f) bond lengths of central COT and NICS(1) (in red); and (g) dihedral angle of unsubstituted COT reported in the literature (CCDC 1320377).^22^

^1^H NMR spectra of **CP3** and **CP4** exhibit simple patterns, reflecting their high symmetries (Figure 2). Distinct sets of signals can be recognized consisting of one singlet associated with the *meso*-protons (H^*a*^), two doublets coupled to each other corresponding to the β-positions (H^*b*^ and H^*c*^), and one singlet from the other β-positions (H^*d*^), similar to that of precursor porphyrin **1** (Figure 2c). The *meso*-protons (H^*a*^) of **CP3** resonate at an extremely high chemical shift (δ = 12.9 ppm) compared with that of **1** (δ = 9.7 ppm), reflecting the proximity between the porphyrin units that puts H^*a*^ in the deshielding region of two porphyrins. However, the *meso*-protons (H^*a*^) of **CP4** is only slightly downfield shifted to 11.0 ppm. ^1^H NMR signals of the 3,5-di-*tert*-butylphenyl groups show that desymmetrization occurs for H^*e*^ and H^*g*^, which appear as two triplets (Figure 2b). The splitting of these signals implies **CP4** adopts a stable *D*_2*d*_ symmetric saddle shape, which cannot convert to other conformers (Figure 3). In contrast, the simple spectrum of **CP3** implies either that the three porphyrin units are coplanar or that the different conformers interconvert rapidly on the NMR time scale. Even when the ^1^H NMR spectrum of **CP3** was recorded at −90 °C (Figure S46), no splitting of ^1^H NMR signals of H^*e*^ could be seen, reflecting the low barrier to conformation exchange. By contrast, for **CP4**, no coalescence of ^1^H NMR peaks H^*e*^ and H^*g*^ was observed, even at 100 °C (Figure S46), demonstrating the high barrier to saddle inversion.

### Crystallography and Computational Modeling

Crystals of **CP3** suitable for X-ray analysis were grown by diffusion of methanol vapor into a solution in 1,2-dichloroethane at room temperature. The structure determination revealed a star-shaped geometry (Figure 3a,b). The dihedral angles around the central benzene ring (measured C_α_–C_β_–C_β′_–C_α′_) are 21.8(19), −18.1(18), and −10.4(17)° (Figure 3a); two of the porphyrin units are twisted, relative to the central benzene, whereas the third is slightly folded. In contrast, DFT calculations predicted that the *D*_3_-symmetric propeller-shaped conformation has the lowest energy in the gas phase. (All DFT calculations in this study were performed with B3LYP/6-31G(d,p), Gaussian 16/A.03; see the Supporting Information for details). The calculated energy difference between the conformation in the crystal and the DFT-optimized geometry of **CP3** is only 4.6 kJ/mol, and coordination of solvent molecules (methanol) to the zinc centers may influence the preferred conformation. In the crystals, two enantiomers were found (P,M,M and P,P,M, where P and M denote right-handed and left-handed helixes, respectively), which form homochiral dimers and these dimers pack alternatively along the *c*-axis (Figure S26).

We were not able to grow suitable single crystals of **CP4** for X-ray analysis, despite screening many solvent systems, although the crystal structure of fullerene complex **CP4**·2C_60_ was determined, as discussed below. According to the DFT calculations, the most stable conformation of **CP4** is saddle-shaped having stereochemistry of P,M,P,M. The lowest energy geometry has a *C*_1_ symmetry, but it is only 0.09 kJ/mol below the idealized *D*_2*d*_ saddle conformation, and these two conformers are more stable than the other diastereoisomers by over 252 kJ/mol (Figure S7). In the lowest energy *C*_1_ symmetric geometry of **CP4**, the angles between the planes of neighboring porphyrin cores (planes each calculated for 24 core C and N atoms) are ∼57.3°, as shown in Figure 3d,e. The angle between the mean plane of each porphyrin and that of the COT ring is 42.7° (Figure 3e). The distance of the outermost β carbon atoms from the mean plane of the central COT is 6.21 Å. The folding angle of the COT unit, defined as the angle between the mean plane of one C–C=C–C unit and the mean plane of the whole COT is 42.2°, which is similar to that in unsubstituted COT (mean 41.9°).^22^

The local aromaticity of the benzene ring at the core of **CP3** can be evaluated from the C–C bond lengths using the harmonic oscillator model of aromaticity (HOMA), which compares the bond lengths around a ring to those in benzene (*R*_opt_ = 1.388 Å).^23^ This analysis gives HOMA values of 0.52(11) and 0.81, from the crystallographic and DFT coordinates, respectively, which indicates that this benzene core has partial aromatic characters (see the Supporting Information for details). The nucleus-independent chemical shift calculated at a position of 1.0 Å above the center of this ring is NICS(1) = −7.5 ppm (for the *D*_3_ geometry of **CP3**), which should be compared with a value of −10.2 ppm for benzene, confirming the partial aromatic character.^24^ The central COT ring in **CP4** has strong bond-length alternation (Figure 3f), giving HOMA values of +0.21 and −0.02(18), from the DFT coordinates and the crystal structure of **CP**_**4**_·2C_60_, respectively. This shows that there is no significant aromatic or antiaromatic delocalization in this core, as found in COT [HOMOA = −0.18(3)].^22^ The NICS(1) for the center of **CP4** is +5.2 ppm, which confirms that it is nonaromatic.

### Photophysics and Electrochemistry

**Figure 4:**
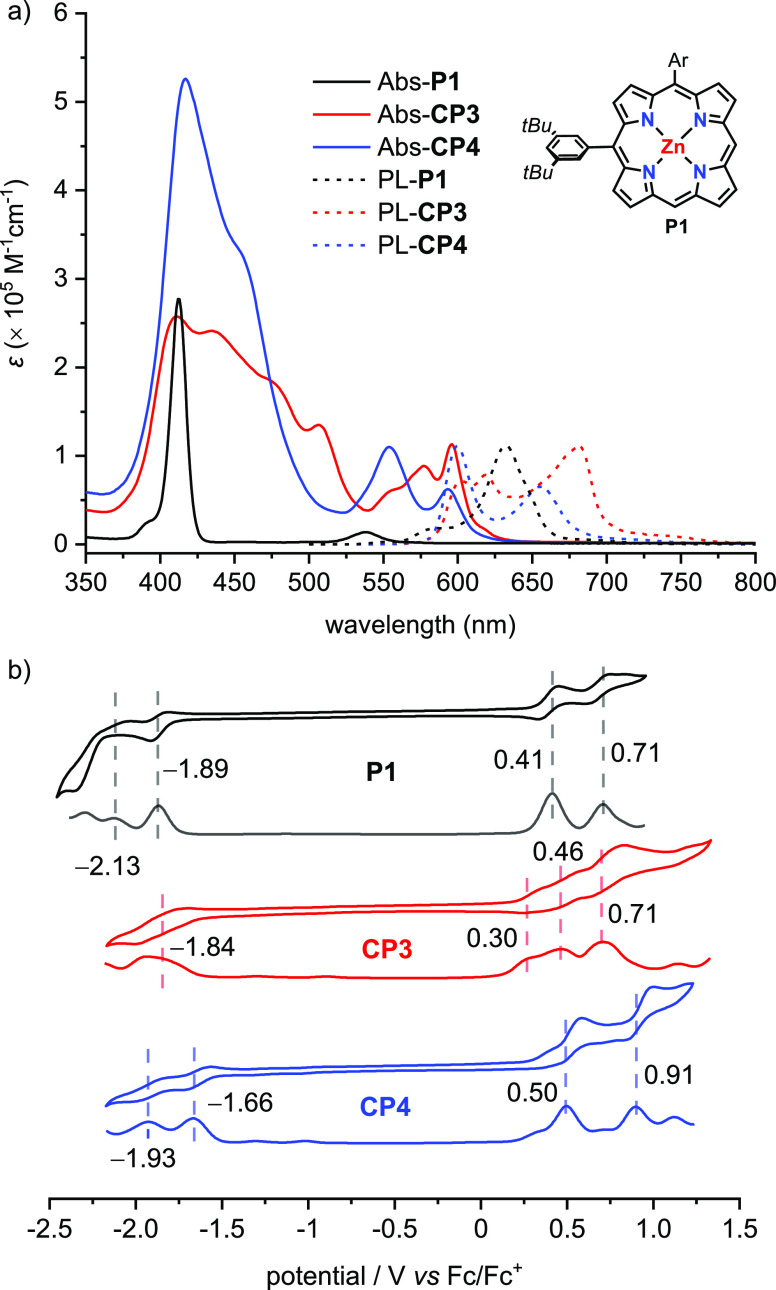
(a) UV–vis absorption and normalized fluorescence spectra of **P1**, **CP3**, and **CP4** measured in toluene (concentration 1 μM); for fluorescence measurements, excitation wavelengths are 417 nm (**P1**), 416 nm (**CP3**), and 415 nm (**CP4**); (b) cyclic and square wave voltammograms of **P1**, **CP3**, and **CP4** measured in dichloromethane with 0.1 M *n*-Bu_4_NPF_6_ at 298 K, scan rate 50 mV/s.

UV–vis absorption and fluorescence spectra of **CP3** and **CP4** are compared with those of monomer **P1**, as shown in Figure 4 (all spectra recorded in toluene). Absorption spectra of both **CP3** and **CP4** show broader Soret bands and red-shifted Q-bands, compared with **P1**. The absorption bands of **CP3** are broader and more red-shifted than those of **CP4**, reflecting stronger electronic coupling and π-conjugation between the porphyrin units in **CP3**. Fluorescence spectra of **CP3** and **CP4** also red shift with respect to **P1** and their predominant emission peaks appear at 681 and 600 nm, respectively. Fluorescence quantum yields are 0.040 and 0.068 in non-deaerated toluene using zinc tetraphenylporphyrin (Φ = 0.029 in toluene) as a standard^25^ and are slightly higher than **P1** (Φ = 0.031).

The electrochemistry of **P1**, **CP3**, and **CP4** was investigated by cyclic voltammetry (CV) and square wave voltammetry (SWV). The CV trace of **P1** exhibits two reversible oxidation peaks at half-wave potentials of 0.41 and 0.71 V and two reduction peaks with half-wave potentials of −1.89 and −2.13 V versus Fc/Fc^+^. **CP3** shows three reversible oxidation peaks at 0.30, 0.46, and 0.71 V as well as one observable reduction peak at −1.84 V. The narrower HOMO–LUMO energy gap of **CP3** reflects the strong π-conjugation between the constituted porphyrins. Two reversible oxidation and reduction peaks located at 0.50, 0.91, −1.66, and −1.93 V were observed for **CP4** within the measured potential range. The fact that the measured redox potentials of **CP4** are close to **P1** reflects the weak electronic communication between the porphyrin units. It is surprising that the first oxidation potential of **CP4** (0.50 V) is higher than that of **P1** (0.41 V); a possible explanation for this effect is that the more sterically congested environment around each porphyrin unit in **CP4** hinders solvation of the positive charge when one of the four porphyrin units becomes oxidized to the radical cation.

### Fullerene Binding

**Figure 5:**
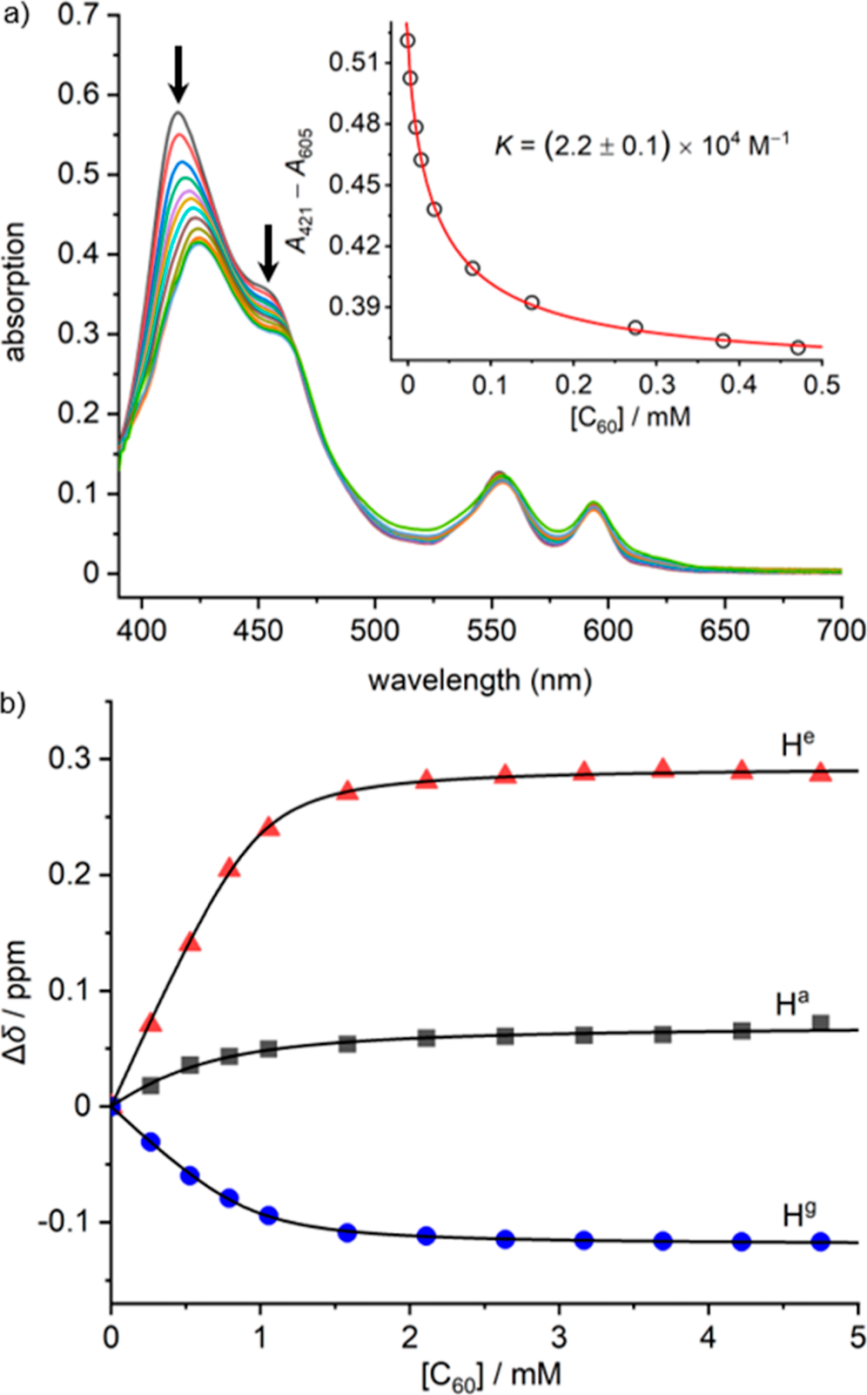
(a) UV–vis absorption spectra change of **CP4** (*c* = 1.65 μM) with the addition of C_60_ (*c* = 0–0.47 mM) in toluene at 298 K, 10 mm path length. Spectra were corrected by subtracting C_60_ absorption; the inset shows plots of *A*_421_–*A*_605_ against the concentration of C_60_, circles indicate the experimental data; (b) plots of chemical shift changes of protons (H^*a*^, H^*e*^, and H^*g*^) on **CP4** (*c* = 0.49 mM) with the addition of C_60_ (400 MHz, *d*_8_-toluene, 298 K).

The design and synthesis of molecular receptors for fullerenes has been a focus of research because of their application in the separation and regioselective functionalization of fullerenes.^4,26^ Although many porphyrin-based fullerene receptors have been reported, all of them have spacers linking the porphyrin units.^4−6^ The saddle-shaped geometry of **CP4** and the cavity between two cofacial porphyrins inspired us to investigate its application as a host for fullerenes. The formation of a **CP4**·2C_60_ complex was first detected by UV–vis titrations in toluene solution (Figure 5a). The Soret band absorption maxima of **CP4** red-shifted from 415 to 424 nm with the addition of C_60_. The binding constant was measured to be (2.2 ± 0.1) × 10^4^ M^–1^ by fitting the titration data to a 1:1 binding isotherm (Table S3). Similar behavior was observed for C_70_, which shows a higher binding constant of (1.1 ± 0.4) × 10^5^ M^–1^. Most porphyrin-based fullerene receptors bind C_70_ more strongly than C_60_ and this selectivity has been used for separating fullerenes.^4,27^ The strong binding of **CP4** with fullerenes was also confirmed by MALDI-TOF MS measurements using *trans*-2-[3-(4-*tert*-butylphenyl)-2-methyl-2-propenylidene]malononitrile (DCTB) as a matrix, which show peaks of the complexes with C_60_ (Figure S23).

The stoichiometry of binding was checked by the ^1^H NMR titration of **CP4** with C_60_. As shown in Figure 5b, the protons (H^*a*^) on the *meso*-positions of porphyrin and the ortho-position of the aryl groups (H^*e*^) all shifted to the lower field region, while the protons on the other ortho-position of the aryl groups (H^*g*^) shifted to the high-magnetic field region with the addition of C_60_. The chemical shift changes approach saturation at around [C_60_]/**CP4** = 2:1. ^1^H NMR titration data fitted well to a 1:1 binding isotherm with a concentration of receptor of 0.98 mM, which is exactly twice the actual concentration of **CP4**, implying that both sides of the receptor bind C_60_ independent of each other, i.e., with no cooperativity.

**Figure 6:**
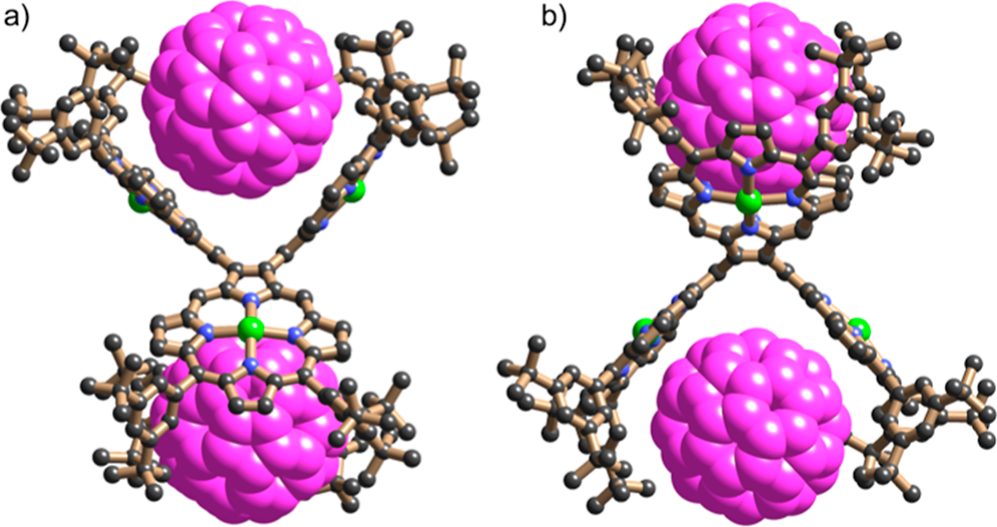
X-ray structure of **CP4**·2C_60_ (orthorhombic phase). Two orthogonal views of the molecular unit; solvent molecules, hydrogen atoms, and disorder omitted for clarity.

Single crystals of complex **CP4**·2C_60_ were obtained by slow diffusion of methanol vapor into a solution of **CP4** and excess C_60_ in *o*-dichlorobenzene. Single-crystal X-ray diffraction studies revealed the 1:2 complex (Figure 6), which crystalized in the triclinic space group *P*1̅. The asymmetric unit was found to contain two molecules of **CP4** and four molecules of C_60_. Examination of the structure suggested the presence of pseudo symmetry, which was thought to potentially be caused by a phase transition. For this reason, the sample was re-examined to explore whether data collected on a high temperature phase would give better results. Despite numerous attempts, the original triclinic phase was not seen again, instead a new orthorhombic phase was repeatedly found. This also revealed a 1:2 complex of **CP4** with C_60_. The data and refinement for this new polymorph were markedly better than the first triclinic phase so this is the one primarily discussed here (though the triclinic polymorph is included in the Supporting Information).^28^

All three crystallographically distinct **CP4**·2C_60_ complexes seen in both crystal structure determinations have essentially the same geometry with methanol molecules coordinated to the central zinc atoms from the side that does not interact with fullerenes. Two fullerene molecules are captured by the cavity between two porphyrins located above and below the central COT ring. The shortest fullerene carbon to zinc distances are in the range of 3.15–3.29 Å, which is shorter than the sum of the van der Waals radi,^29^ indicating an attractive interaction between fullerene and porphyrins. Each porphyrin skeleton adopts a saddle distortion, with a concave face towards a bound C_60_ molecule. The geometry of the **CP4** unit in this crystal of the C_60_ complex is similar to the DFT-calculated structure of **CP4** (Figure 3), which is consistent with the non-cooperative binding behavior deduced from UV–vis absorption and ^1^H NMR titration data.

## Conclusions

We have reported the synthesis of two 2,3-linked cyclic porphyrin oligomers **CP3** and **CP4**, with benzene and COT cores, via Yamamoto coupling of 2,3-dibromoporphyrin. The Yamamoto coupling of vicinal dibromides has previously been applied to link together various other conjugated π-systems, such as helicenes,^30^ acepleiadylene,^31^ and tetracenes,^32^ but in most cases, this reaction gives just the benzene-centered cyclic trimer, not the COT-centered cyclic tetramer. The different numbers of porphyrin units in **CP3** and **CP4** result in different 3D geometries, leading to distinct optoelectronic properties. Although the minimum-energy geometry of **CP3** has a *D*_3_ propeller-shaped structure, its crystal structure adopts a lower symmetry P,M,M/M,P,P configuration, whereas **CP4** exists in a stable saddle-shaped conformation with stereochemistry of P,M,P,M. π-Conjugation in **CP3** results in splitting and red shift of the absorption bands. By contrast, the saddle-shaped **CP4** shows similar optical and electrochemical properties to the monomer, due to the large dihedral angles between porphyrin units. The saddle-shaped geometry of **CP4** makes it a good receptor for fullerene and high binding constants of (1.1 ± 0.4) × 10^5^ and (2.2 ± 0.1) × 10^4^ M^–1^ were obtained for C_70_ and C_60_, respectively, in toluene solution at 298 K. The 1:2 fullerene binding mode of **CP4** could be useful for holding two paramagnetic endohedral fullerenes at a fixed distance for quantum information processing.^5b,33^ This work also provides new insights into the design and synthesis of directly linked cyclic porphyrin arrays with applications in photophysics and supramolecular chemistry.

## Supporting Information Available

Experimental details, characterization data, ^1^H NMR, ^13^C NMR, and MS spectra, and calculation details (PDF)
