# Supplementary material for: β,β-Directly Linked Porphyrin Rings: Synthesis, Photophysical Properties, and Fullerene Binding
Source: J Am Chem Soc. 2023 May 18;145(21):11859–65. doi: 10.1021/jacs.3c03549 (PMC10236496; doi:10.1021/jacs.3c03549)
Supplement: Supplementary file 1 — ja3c03549_si_001.pdf [file ja3c03549_si_001.pdf]

Supporting Information for

β,β-DIRECTLY LINKED PORPHYRIN RINGS: SYNTHESIS, PHOTOPHYSICAL PROPERTIES AND FULLERENE BINDING

Qiang Chen,[†] Amber L. Thompson,[†] Kirsten E. Christensen,[†] Peter N. Horton,[‡] Simon J. Coles,[‡] and Harry L. Anderson*[,†]

[†]Department of Chemistry, University of Oxford, Chemistry Research Laboratory, Oxford OX1 3TA, U.K.
[‡]National Crystallography Service, School of Chemistry, University of Southampton, Southampton SO17 1BJ, U.K.

Correspondence to: Harry L. Anderson (harry.anderson@chem.ox.ac.uk)

Table of Contents

General Procedures S2
Synthetic Details S3
UV-vis Absorption, Fluorescence and Excitation Spectra S6
DFT Calculations S10
HOMA Calculations S12
UV-Visible, Fluorescence and ^{1}H NMR Binding Study of **CP4** with Fullerenes S14
X-ray Crystallographic Analysis S20
NMR Spectra S26
MALDI-TOF MS Spectra S36
References S39
Calculated Molecular Cartesian Coordinates S40–83

General Procedures

All commercially available reagents were used as received. Dry solvents (dichloromethane, chloroform, *N,N*-dimethylformamide and toluene) used for reactions were purified by a MBraun MB-SPS-5 bench-top solvent purification system under nitrogen (H_2O content < 20 ppm). Unless otherwise noted, reactions were carried out in oven-dried glassware under an argon atmosphere. Thin layer chromatography (TLC) was carried out on aluminum-backed silica gel plates with 0.2 mm thick silica gel 60 F254 (Merck) and visualized by UV irradiation at either 254 nm or 366 nm. Preparative flash column chromatography was carried out using flash silica gel 60 (230–400 mesh) obtained from Sigma-Aldrich. ^1H and ^{13}C nuclear magnetic resonance (NMR) spectra were recorded on Bruker AVIII HD 400, AVIII HD 500, Bruker AVIII HD 600 (Prodigy broadband cryoprobe) NMR spectrometer, respectively at 298 K unless otherwise stated. NMR chemical shifts were expressed in ppm relative to the internal residual solvent peaks using the reported values (dimethyl sulfoxide-d_6, ^1H: 2.50 ppm, ^{13}C: 39.52 ppm; dichloromethane-d_2, ^1H: 5.32 ppm, ^{13}C: 54.00 ppm; chloroform-*d*, ^1H: 7.26 ppm, ^{13}C: 77.16 ppm). Coupling constants are reported in Hz and ^1H multiplicities are reported in accordance with the following: s = singlet; d = doublet; t = triplet; q = quartet; and m = multiplet; br = broad singlet. ^1H assignments were made using 2D NMR methods (COSY, NOESY, HSQC, HMBC). High-resolution mass (HR-MS) measurements were performed on a Thermo Exactive High-Resolution Orbitrap FTMS spectrometer. UV-vis spectra were recorded in non-deaerated toluene solution on a Perkin-Elmer Lambda 25 spectrometer at 25 °C, using quartz cuvettes with a light pathlength of 1.0 cm. Fluorescence spectra were acquired in solution in fused silica cuvettes at 25 °C using an Edinburgh Instruments FS5 spectrofluorometer operating Fluoracle® software and equipped with a xenon arc lamp (providing 230–1000 nm excitation range) and an R13456 PMT detector (200–950 nm spectral coverage, Hamamatsu). Cyclic voltammetry measurements were made using an Autolab PGSTAT 12 with a 3 mm glassy carbon working electrode, platinum wire counter electrode and $Ag/AgNO_3$ (0.01 M in acetonitrile) reference electrode at a rate of 50 mV/s with a 10 mV step potential. Tetra-*n*-butylammonium hexafluorophosphate (n-$Bu_4N{\cdot}PF_6$) at a concentration of 0.1 M was used as supporting electrolyte. Voltammograms were referenced to the Fc/Fc^+ couple (0.0 V) as an internal reference. Square wave voltammograms were acquired with a 5 mV step potential, 50 mV modulation amplitude and 5 Hz frequency at a rate of 25 mV/s.

Synthetic Details

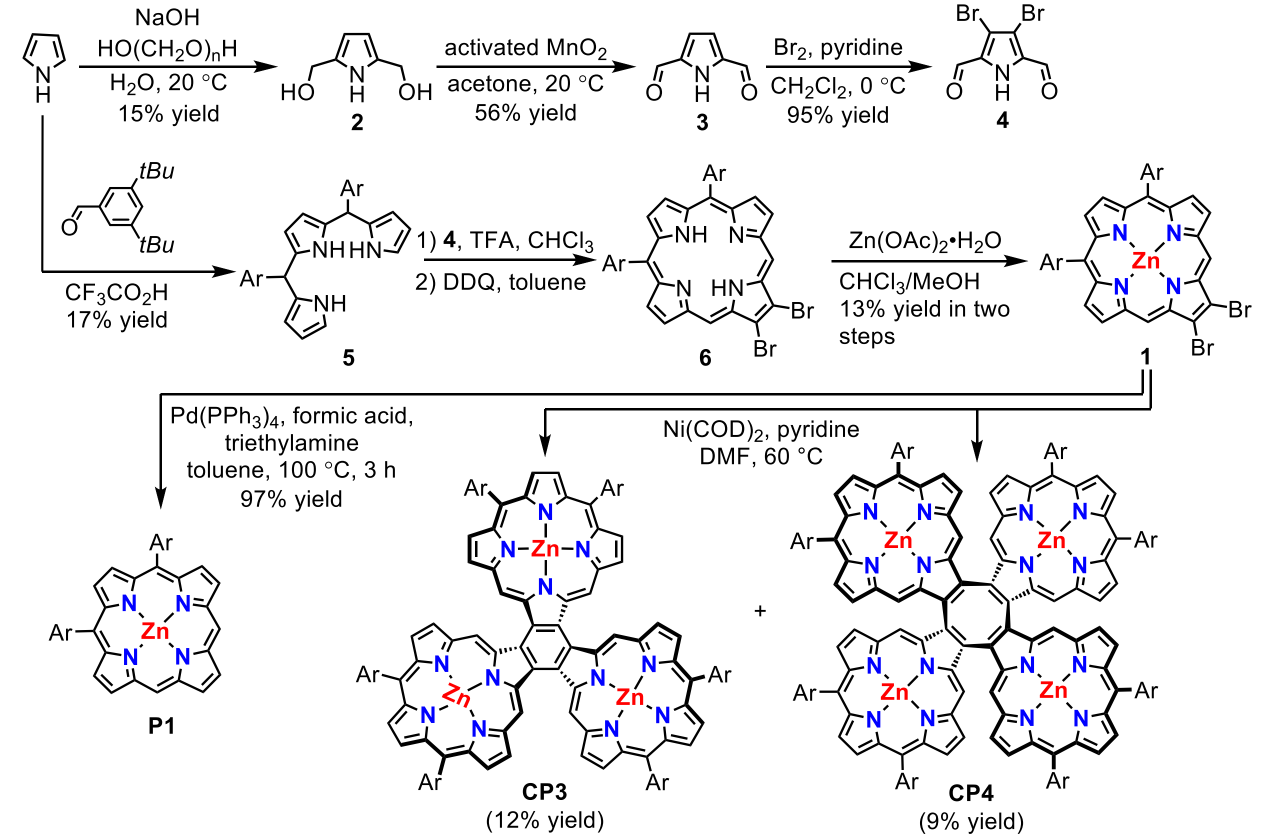

Scheme S1. Synthetic route towards **P1**, **CP3** and **CP4**.

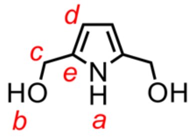

Synthesis of 2,5-bis(hydroxymethyl)pyrrole (2). A mixture of pyrrole (15.5 mL, 0.223 mol), paraformaldehyde (13.47 g) and NaOH solution (1.0 M aq., 0.2 mL) was stirred at 25 °C for 3 days in a 50 mL round-bottom flask. The transparent liquid was poured into dichloromethane (100 mL) and the resulting suspension was stirred for 30 min. The white solid was collected by filtration, washed with dichloromethane (50 mL), and dried under vacuum to give compound **2** (4.23 g, 15 % yield) as white powder. The characterization data are in accord with those reported.[1] ^{1}H NMR (400 MHz, DMSO-d_6, 298 K) δ 10.48 (s, 1H, H^a), 5.77 (d, J = 2.4 Hz, 2H, H^d), 4.73 (t, J = 5.5 Hz, 2H, H^b), 4.33 (d, J = 5.5 Hz, 4H, H^c); ^{13}C NMR (101 MHz, DMSO-d_6, 298 K) δ 132.0 (C^e), 105.4 (C^d), 56.3 (C^c); HR MS (ESI, negative): calcd for $C_6H_9NO_2$, m/z = 126.0560, found 126.0551 $[M-H]^-$.[1]

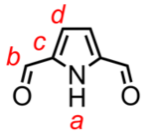

Synthesis of 2,5-diformylpyrrole (3). To a solution of 2,5-bis(hydroxymethyl)pyrrole **2** (4.11 g, 32.4 mmol) in acetone (25 mL) was added activated manganese dioxide (3.42 g, 39.3 mmol). After stirring the suspension at 25 °C for 20 h, the reaction mixture was filtered and the filter cake was washed with acetone (250 mL) four times. The filtrate was evaporated under reduced pressure and the resulting residue was purified by flash column chromatography over silica gel (ethyl acetate / petroleum ether = 1:3 *v*/*v*) to give compound **3** (2.23 g, 56% yield) as white solid. The characterization data are in accordance with those reported.[2] ^{1}H NMR (400 MHz, $CDCl_3$, 298 K) δ 10.22 (s, 1H, H^a), 9.78 (s, 2H, H^b), 7.01 (d, J = 2.4 Hz, 2H, H^d); ^{13}C NMR (101 MHz, $CDCl_3$, 298 K) δ 181.4 (C^b), 135.8 (C^c), 119.6 (C^d); HR MS (ESI, negative): calcd for $C_6H_5NO_2$, m/z = 122.0247, found 122.0239 $[M-H]^-$.[2]

Synthesis of 3,4-dibromo-2,5-diformylpyrrole (4). To a solution of 2,5-diformylpyrrole **3** (1.1 g, 8.9 mmol) in pyridine (2.16 mL, 26.8 mmol), dichloromethane (120 mL) and acetic acid (40 mL) was added a solution of bromine (1.05 mL, 20.6 mmol) in acetic acid (20 mL) at 0 °C. After stirring at 0 °C for 2 h, the mixture was diluted with dichloromethane (60 mL), washed with water (50 mL) three times, brine (50 mL), dried over Na_2SO_4 and the solvent was evaporated under reduced pressure. The residue was purified by silica gel column chromatography (ethyl acetate / petroleum ether = 1:3, *v*/ *v*) to give compound **4** (2.39 g, 95% yield) as white solid. ^{1}H NMR (400 MHz, DMSO-d_6, 298 K) δ 12.53 (s, 1H, H^a), 9.84 (s, 2H, H^b); ^{13}C NMR (101 MHz, DMSO-d_6, 298 K) δ 180.6 (C^b), 131.4 (C^c), 108.0 (C^d); HR MS (ESI, negative): calcd for $C_6H_3Br_2NO_2$, *m/z* = 277.8458, found 277.8457 $[M - H]^-$; in accord with reported data.[3]

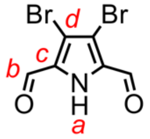

Synthesis of tripyrrane 5. To a mixture of 3,5-di-*tert*-butylbenzaldehyde (500 mg, 2.29 mmol) in pyrrole (768 mg, 11.5 mmol) was added trifluoroacetic acid (17.5 μL, 229 μmol) and the resulting mixture was stirred at 25 °C for 30 minutes. After quenching with saturated aqueous Na_2CO_3 (10 mL), the mixture was extracted with ethyl acetate (50 mL). The organic phase was separated, washed with brine (30 mL), dried over Na_2SO_4 and evaporated. The residue was purified by silica gel column chromatography (ethyl acetate / petroleum ether/ triethylamine = 33:99:1, *v*/ *v*), followed by preparative size exclusion chromatography (Bio-beads SX-3, $CHCl_3$ / pyridine = 100:1, *v*/ *v*) to give tripyrrane **5** (240 mg, 17% yield) as brown solid (mixture of diastereoisomers). ^{1}H NMR (400 MHz, $CDCl_3$, 298 K) δ 7.87 (s, 2H, H^a), 7.73 (s, 1H, H^j), 7.29 (q, *J* = 1.9 Hz, 2H, H^g), 7.02 (d, *J* = 1.8 Hz, 4H, H^f), 6.65 (ddd, *J* = 4.4, 2.6, 1.7 Hz, 2H, H^b), 6.14 – 6.08 (m, 2H, H^c), 5.89 – 5.84 (m, 2H, H^d), 5.81 (dd, *J* = 4.2, 2.7 Hz, 2H, H^i), 5.36 (s, 2H, H^e), 1.26 (s, 36H, H^h); ^{13}C NMR (101 MHz, $CDCl_3$, 298 K) δ 151.0, 141.1, 141.0, 133.21, 133.17, 132.6, 132.5, 122.8 (C^f), 122.7 (C^f), 121.0 (C^g), 120.9 (C^g), 116.98 (C^b), 116.97 (C^b), 108.5 (C^c), 107.4 (C^d/ C^i), 107.2 (C^i / C^d), 44.80 (C^e), 35.0 ($\underline{C}$-C^h), 31.7 (C^h), 31.6 (C^h); HR MS (ESI, positive): calcd for $C_{42}H_{55}N_3$, *m/z* = 602.4469, found 602.4472 $[M + H]^+$; in accord with reported data.[4]

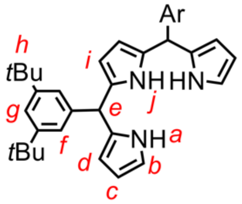

Synthesis of 2,3-dibromo-10,15-bis(3,5-di-*tert*-butylphenyl)porphyrin (6). To a vigorously stirred solution of trifluoroacetic acid (3.32 mL, 43.4 mmol) in chloroform (43 mL), solutions of 3,4-dibromo-2,5-diformylpyrrole **4** (122 mg, 434 μmol) in chloroform (6 mL) and tripyrrane **5** (261 mg, 434 μmol) in chloroform (6 mL) were simultaneously added during 5 min. The reaction mixture was stirred at room temperature for 15 min before a solution of 2,3-dichloro-5,6-dicyano-1,4-benzoquinone (DDQ) (98.6 mg, 434 μmol) in toluene (8 mL) was added. After an additional 10 min of stirring, the reaction mixture was quenched using triethylamine (3 mL). The solvent was evaporated and the residue was purified by column chromatography (dichloromethane/petroleum ether = 1/5, *v*/ *v*) to give 2,3-dibromo-10,15-bis(3,5-di-*tert*-butylphenyl)porphyrin **6** (54.5 mg, 15% yield) as a purple solid. ^{1}H NMR (400 MHz, $CDCl_3$, 298 K) δ 10.31 (s, 2H, H^a), 9.46 (d, *J* = 4.9 Hz, 2H, H^b), 9.15 (d, *J* = 4.8 Hz, 2H, H^c), 8.87 (s, 2H, H^d), 8.09 (d, *J* = 1.8 Hz, 4H, H^e), 7.84 (t, *J* = 1.8 Hz, 2H, H^f), 1.55 (s, 38H, H^g), –3.31 (s, 2H, H^h); ^{13}C NMR (101 MHz, $CDCl_3$, 298 K) δ 155.8, 149.1, 140.9, 138.9, 138.0, 135.1 (C^d), 130.0 (C^e), 129.4 (C^c), 128.2 (C^b), 124.9, 122.6, 121.5 (C^f), 101.9, 35.2($\underline{C}$-C^g), 31.9 (C^g); MALDI-TOF MS (positive): calcd for $C_{48}H_{52}Br_2N_4$, *m/z* = 842.26, found 842.31 $[M^+]$; UV-vis (toluene): λ (ε) = 418 nm (3.97×10^5 $M^{-1}cm^{-1}$), 510 nm (1.92×10^4 $M^{-1}cm^{-1}$), 548 nm (1.13×10^4 $M^{-1}cm^{-1}$), 584 nm (7.00×10^3 $M^{-1}cm^{-1}$) and 644 nm (6.93×10^3 $M^{-1}cm^{-1}$).

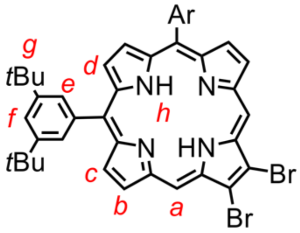

Synthesis of 2,3-dibromo-10,15-bis(3,5-di-*tert*-butylphenyl)porphyrin (Zn) (1). To a solution of 2,3-dibromo-10,15-bis(3,5-di-*tert*-butylphenyl)porphyrin **6** (54 mg, 64 μmol) in chloroform (20 mL) was added a solution of $Zn(OAc)_2 \cdot 2H_2O$ (46 mg, 0.32 mmol) in MeOH (1 mL). After heating at 65 °C under argon for 1 h, the reaction mixture was cooled to room temperature. The mixture was diluted with petroleum ether (30 mL) and passed through a short silica gel column, eluting with petroleum ether/dichloromethane = 3:1 (*v*/*v*). After evaporating the solvent, the residue was washed with MeOH and collected by filtration to give porphyrin **1** (50 mg, 86% yield) as red solid. ^{1}H NMR (600 MHz, $CDCl_3$, 298 K) δ 9.96 (s, 2H, H^a), 9.29 (d, J = 4.4 Hz, 2H, H^b), 9.11 (d, J = 4.4 Hz, 2H, H^c), 9.06 (s, 2H, H^d), 8.13 (d, J = 1.7 Hz, 4H, H^e), 7.84 (t, J = 1.6 Hz, 2H, H^f), 1.57 (s, 38H, H^g); ^{13}C NMR (151 MHz, $CDCl_3$, 298 K) δ 150.7(4), 150.6(7), 149.8, 148.9, 142.3, 141.6, 133.4 (C^c), 132.8 (C^d), 132.1 (C^b), 130.0 (C^e), 129.1 (C^f), 123.6, 121.2, 101.0 (C^a), 35.2 ($\underline{C}$-C^g), 31.9 (C^g); MALDI-TOF MS (positive): calcd for $C_{48}H_{50}Br_2N_4Zn$, *m/z* = 904.17, found 904.30 [M^+]; UV-vis (toluene): λ (ε) = 419 nm (4.38×10^5 $M^{-1}cm^{-1}$), 544 nm (2.05×10^4 $M^{-1}cm^{-1}$) and 580 nm (4.09×10^3 $M^{-1}cm^{-1}$).

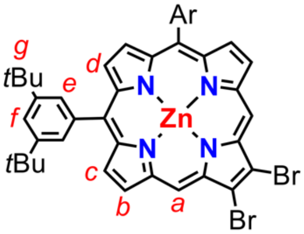

Synthesis of 5,10-bis(3,5-di-*tert*-butylphenyl)porphyrin (Zn) (P1). To a 10 mL Schlenk tube was added 2,3-dibromo-10,15-bis(3,5-di-*tert*-butylphenyl)porphyrin (Zn) **1** (5.0 mg, 5.5 μmol), $Pd(PPh_3)_4$ (0.6 mg, 0.6 μmol), triethylamine (50 μL, 0.4 mmol), formic acid (50 μL, 1 mmol) and degassed toluene (1.5 mL). The mixture was heated at 100 °C for 3 h under argon atmosphere, and then cooled to room temperature. After evaporation of the solvent, the residue was purified by silica gel column chromatography (petroleum ether/dichloromethane = 3:1, *v*/*v*) to give **P1** (4.0 mg, 97% yield) as red solid. ^{1}H NMR (600 MHz, $CDCl_3$, 298 K) δ 10.24 (s, 2H, H^b), 9.48 (s, 2H, H^a), 9.42 (d, J = 4.4 Hz, 2H, H^c), 9.17 (d, J = 4.4 Hz, 2H, H^d), 9.09 (s, 2H, H^e), 8.12 (d, J = 1.7 Hz, 4H, H^f), 7.82 (d, J = 1.7 Hz, 2H, H^g), 1.55 (s, 36H, H^h); ^{13}C NMR (151 MHz, $CDCl_3$, 298 K) δ 150.18, 150.17, 150.0, 149.9, 148.7, 141.9, 133.1 (C^d), 132.3 (C^a/ C^e), 132.2 (C^e/ C^a), 131.6 (C^c), 129.9 (C^f), 122.6, 121.0 (C^g), 105.2 (C^b), 35.2 ($\underline{C}$-C^h), 31.9 (C^h); MALDI-TOF MS (positive): calcd for $C_{48}H_{52}N_4Zn$, *m/z* = 748.35, found 748.38 [M^+]; UV-vis (toluene): λ (ε) = 412 nm (2.77×10^5 $M^{-1}cm^{-1}$) and 539 nm (1.20×10^4 $M^{-1}cm^{-1}$); in accord with reported data.[5]

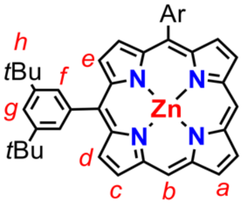

Cyclic porphyrin trimer CP3 and tetramer CP4. To a 25-mL Schlenk tube was added 2,3-dibromo-10,15-bis(3,5-di-*tert*-butylphenyl)porphyrin (Zn) **1** (40.0 mg, 44.1 μmol) and $Ni(COD)_2$ (48.5 mg, 176 μmol). The tube was evacuated and backfilled with argon for three times before DMF (8.8 mL) and pyridine (88 μL) were added. After degassing by three freeze-pump-thaw cycles, the mixture was heated at 60 °C for 18 h. After cooling to room temperature, ethyl acetate (50 mL) was added and the mixture was washed with water (50 mL), brine (30 mL) and dried over Na_2SO_4. The solvent was evaporated and the residue was passed through a short silica plug, eluting with dichloromethane. After evaporation of solvent, the residue was purified by recycling gel permeation chromatography (toluene/pyridine = 100:1, *v*/*v*) to give **CP3** (4.1 mg, 12% yield) and **CP4** (3.0 mg, 9% yield) as brown solids.

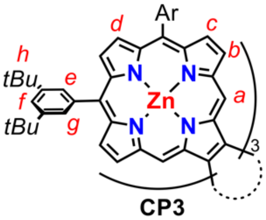

CP3

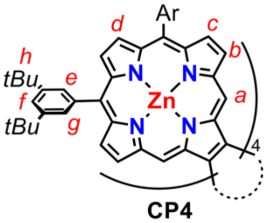

CP4

CP3: ^{1}H NMR (500 MHz, CD_2Cl_2, 298 K) δ 12.96 (s, 6H, H^a), 9.83 (d, J = 4.2 Hz, 6H, H^b), 9.35 (d, J = 4.2 Hz, 6H, H^c), 9.09 (s, 6H, H^d), 8.30 (d, J = 1.8 Hz, 12H, $H^{e,g}$), 7.93 (t, J = 1.8 Hz, 6H, H^f), 1.64 (s, 108H, H^h); ^{13}C NMR (126 MHz, CD_2Cl_2) δ 151.0, 150.4, 150.1, 149.7, 149.4, 149.2, 149.1, 147.2, 142.8, 136.2, 135.9, 135.7, 135.5, 133.5 (C^c), 132.0 (C^b/C^d), 130.4 (C^e), 123.5, 121.3 (C^f), 105.9 (C^a), 35.4 ($\underline{C}$-C^h), 32.0 (C^h); MALDI-TOF MS (positive): calcd for $C_{144}H_{150}N_{12}Zn_3$, *m/z* = 2239.00, found 2238.92 [M^+]; UV-vis (toluene): λ (ε) =

411 nm (2.57 × 10^5 $M^{-1}cm^{-1}$), 435 nm (2.41 × 10^5 $M^{-1}cm^{-1}$), 506 nm (1.35 × 10^5 $M^{-1}cm^{-1}$), 577 nm (8.86 × 10^4 $M^{-1}cm^{-1}$) and 596 nm (1.13 × 10^5 $M^{-1}cm^{-1}$).

CP4: ^{1}H NMR (600 MHz, CD_2Cl_2, 298 K) δ 11.20 (s, 8H, H^a), 9.11 (d, J = 4.7 Hz, 8H, H^b), 9.05 (d, J = 3.9 Hz, 16H, $H^{c,d}$), 8.21 (s, 8H, H^e/H^g), 8.00 (s, 8H, H^g/H^e), 7.81 (t, J = 1.8 Hz, 8H, H^f), 1.58 (s, 72H, H^h), 1.42 (s, 72H, H^h); ^{13}C NMR (151 MHz, CD_2Cl_2) δ 151.0, 150.8, 150.4, 149.2, 143.8, 142.0, 133.2 (C^c), 132.7 (C^b), 132.6 (C^b), 130.2 (C^g/C^e), 130.1 (C^e/C^g), 123.0, 121.5 (C^f), 114.2, 107.6 (C^a), 107.5 (C^a), 31.9 ($\underline{C}$-C^h), 31.8 ($\underline{C}$-C^h), 30.1 (C^h); MALDI-TOF MS (positive): calcd for $C_{192}H_{200}N_{16}Zn_4$, *m/z* = 2985.33, found 2985.88 [M^+]; UV-vis (toluene): λ (ε) = 417 nm (5.25 × 10^5 $M^{-1}cm^{-1}$), 554 nm (1.09 × 10^5 $M^{-1}cm^{-1}$) and 593 nm (5.92 × 10^4 $M^{-1}cm^{-1}$).

UV-vis Absorption, Fluorescence and Excitation Spectra

All measurements were carried out in non-deaerated toluene solution at 298 K. UV-vis absorption spectra of **P1**, **CP3** and **CP4** were measured at concentration of 10^{-6} M; for fluorescence measurement, excitation wavelengths are 417 nm (**P1**), 424 nm (**CP3**) and 420 nm (**CP4**). Fluorescence lifetimes were measured in time-correlated single photon counting (TCSPC) mode using a picosecond pulsed diode laser (EPL-475, λ = 473.5 nm) as the excitation source and detection at 631 nm, 680 nm and 656 nm for **P1**, **CP3** and **CP4**, respectively. Fluorescence quantum yields were measured using tetraphenylporphyrin(Zn) (**ZnTPP**) (Φ = 0.029 in non-deaerated toluene) as standard.[6] Fluorescence quantum yields were calculated using the formula:

$$\Phi_s = \Phi_r \cdot \frac{F_s}{F_r} \cdot \frac{\left[1 - 10^{-A(\lambda_{Ex})}\right]_r}{\left[1 - 10^{-A(\lambda_{Ex})}\right]_s}$$

where subscripts (s) and (r) refer to sample and reference, Φ is the fluorescence quantum yield, F is the integral photon flux, $1 - 10^{-A(\lambda_{Ex})}$ is the absorption factor at the wavelength of excitation.[7]

Table S1. Summary of photophysical properties of **P1**, **CP3** and **CP4**.

compound	λ_{abs} (nm), ε ($M^{-1}cm^{-1}$)	λ_{em} (nm)	τ_f (ns)	Φ_f	k_{rad} (μs^{-1})
**P1**	412 (2.77 × 10^5); 539 (1.20 × 10^4)	584, 632	2.94	0.031	10.5
**CP3**	411 (2.57 × 10^5); 435 (2.41 × 10^5); 506 (1.35 × 10^5); 577 (8.86 × 10^4); 596 (1.13 × 10^5)	603, 618, 681	1.49	0.040	26.8
**CP4**	415 (5.25 × 10^5); 554 (1.09 × 10^5); 593 (5.92 × 10^4)	600, 656	2.42	0.068	28.1

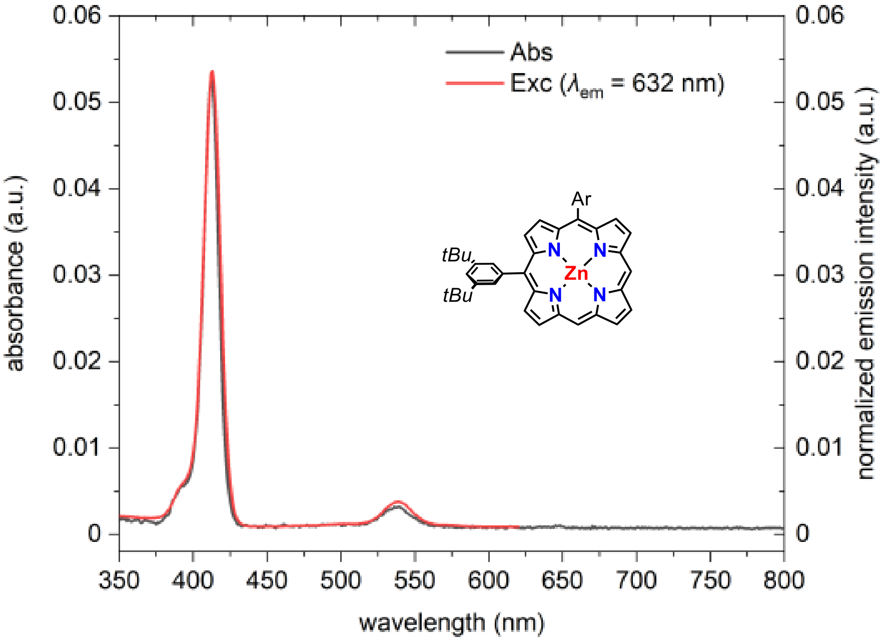

Figure S1. Comparison of UV-vis absorption and fluorescence excitation spectra of **P1** measured in toluene solution at 25 °C.

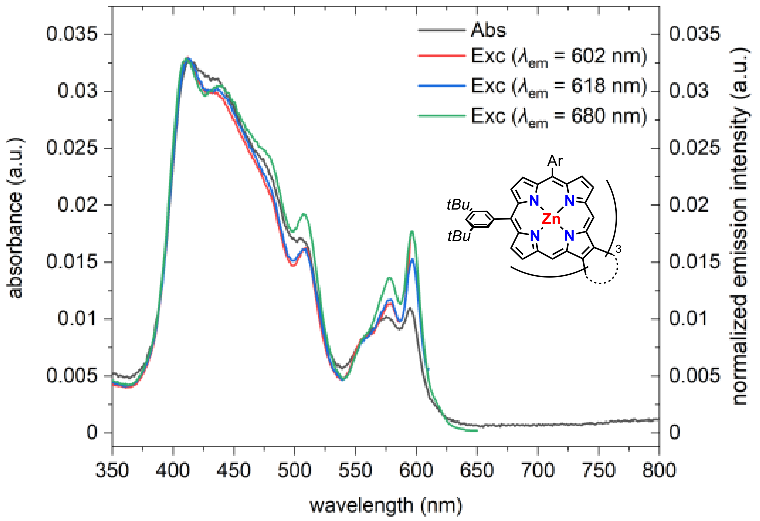

Figure S2. Comparison of UV-vis absorption and fluorescence excitation spectra of **CP3** measured in toluene solution at 25 °C.

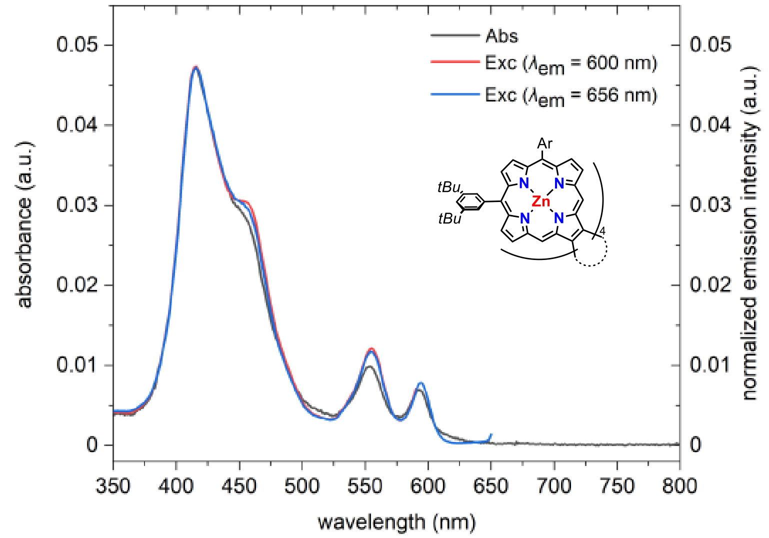

Figure S3. Comparison of UV-vis absorption and fluorescence excitation spectra of **CP4** measured in toluene solution at 25 °C.

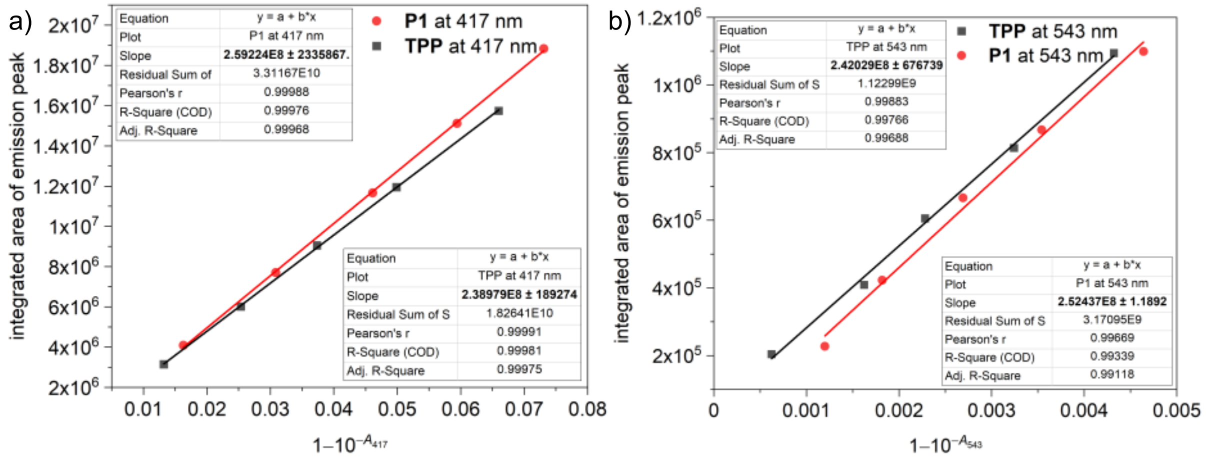

Figure S4. Plots of integrated emission peak (560–750 nm) against $1\text{–}10^{-A(\lambda Ex)}$ value of reference compound **TPP(Zn)** and **P1** in toluene at 298 K. Two independent measurements were conducted with the excitation wavelength at a) 417 nm and b) 543 nm, respectively. The average fluorescence quantum yield of **P1** is calculated to be 0.031.

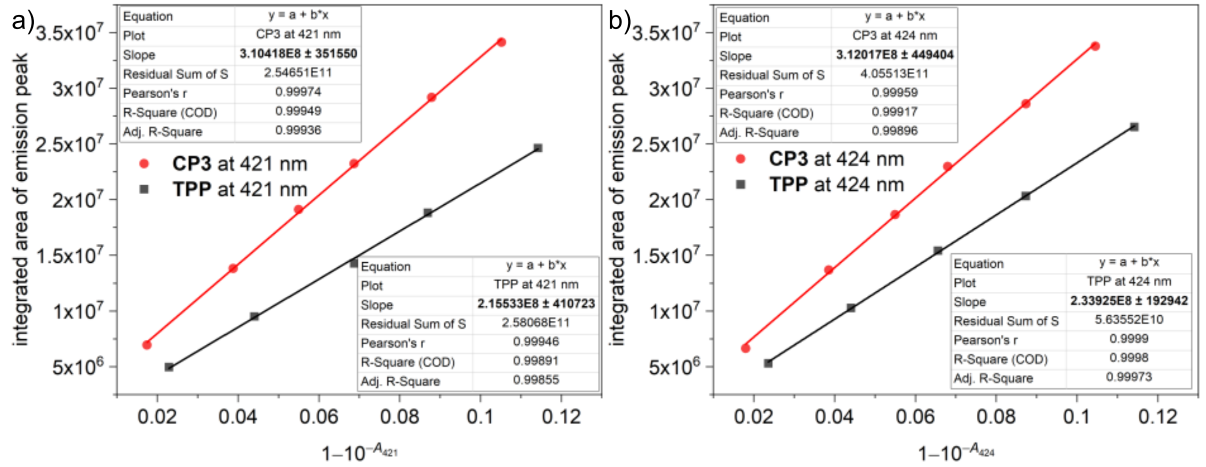

Figure S5. Plots of integrated emission peak (560–750 nm) against $1\text{–}10^{-A(\lambda Ex)}$ value of reference compound **TPP(Zn)** and **CP3** in toluene at 298 K. Two independent measurements were conducted with the excitation wavelength at a) 421 nm and b) 424 nm, respectively. The average fluorescence quantum yield of **CP3** is calculated to be 0.040.

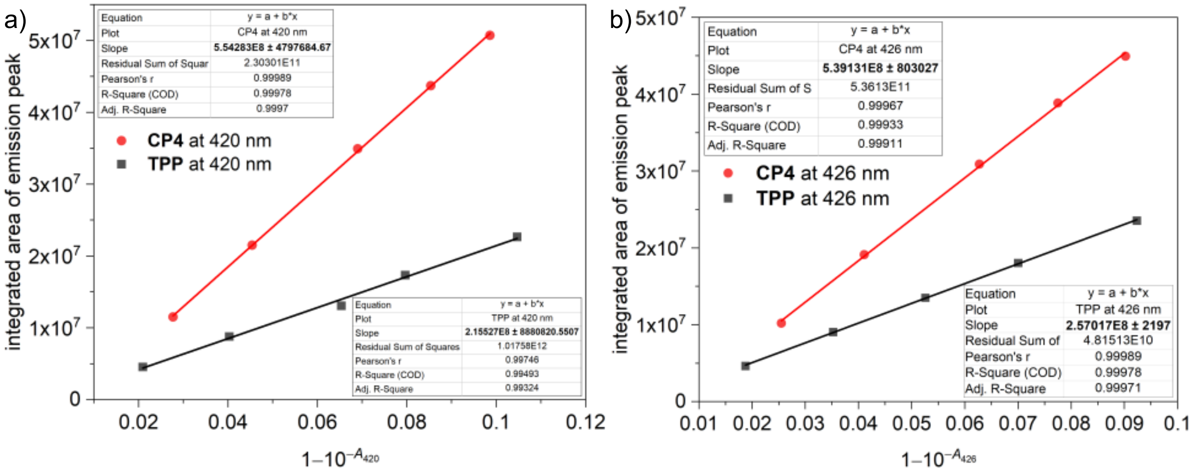

Figure S6. Plots of integrated emission peak (560–750 nm) against $1-10^{-A(\lambda Ex)}$ value of reference compound **TPP(Zn)** and **CP4** in toluene at 298 K. Two independent measurements were conducted with the excitation wavelength at a) 420 nm and b) 426 nm, respectively. The average fluorescence quantum yield of **CP4** is calculated to be 0.068.

DFT Calculations

DFT calculations were performed using Gaussian 16/A.03 software package.[8] Geometries were optimized for each conformation of **CP3** and **CP4** using B3LYP level of theory and 6-31G(d,p) basis set. Nucleus independent chemical shifts (NICS) were calculated using the gauge invariant atomic orbital (GIAO) approach, as implemented in Gaussian 16/A.03, at the GIAO-B3LYP/6-31G(d,p) level.[9]

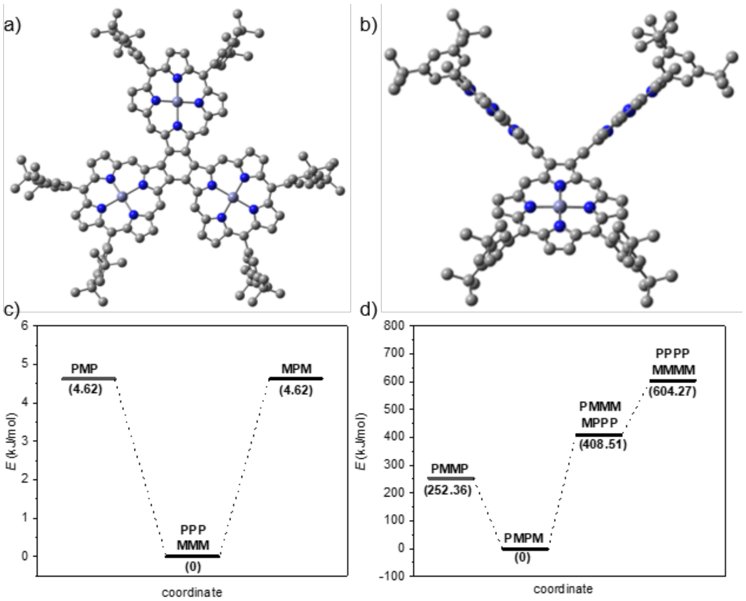

Figure S7. The lowest-energy geometries of a) **CP3** (only PPP conformer was shown) and b) **CP4** calculated at the B3LYP/6-31G(d,p) level of theory and the relative energy of the other conformation of c) **CP3** and d) **CP4** calculated using the same method.

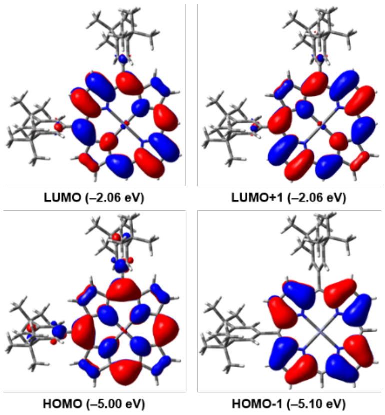

Figure S8. Frontier molecular orbitals and energy levels of **P1** calculated by DFT at the B3LYP/6-31G(d,p) level and it has doubly degenerate LUMO and nearly degenerate HOMO.

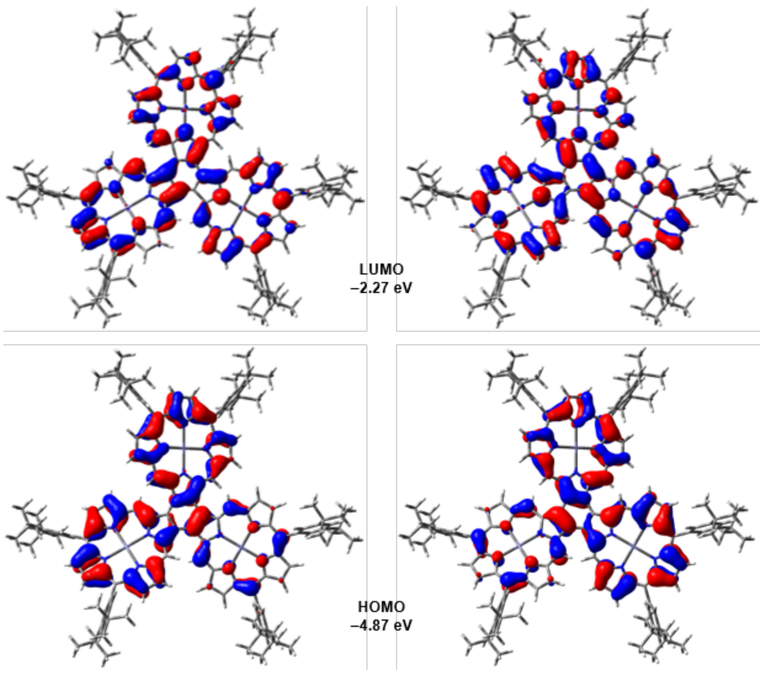

Figure S9. Frontier molecular orbitals and energy levels of **CP3** calculated by DFT at the B3LYP/6-31G(d,p) level and it has doubly degenerate HOMO and LUMO.

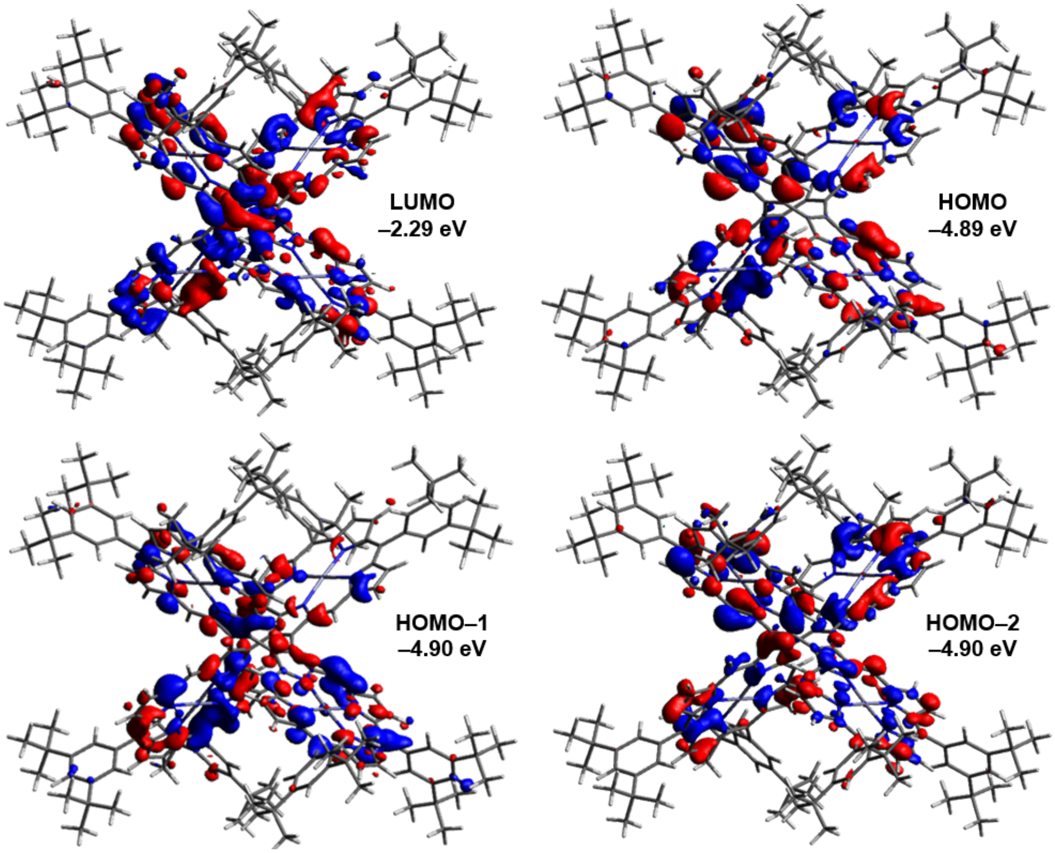

Figure S10. Frontier molecular orbitals and energy levels of **CP4** calculated by DFT at the B3LYP/6-31G(d,p) level and it has degenerate HOMO.

HOMA Calculations

HOMA (Harmonic Oscillator Model of Aromaticity) is one of the simplest and most successful indices for describing aromaticity based on molecular geometry. It uses the C-C bond length in benzene as a standard of perfect aromaticity. The HOMA index can be calculated using the following equation:[10]

$$\mathrm{HOMA} = 1 - \frac{\alpha}{n}\sum_{i=1}^{n}(R_i - R_{\mathrm{opt}})^2$$

where R_i and R_{opt} are the ith bond length of the C-C bond in the analyzed ring and the bond length of benzene ring (R_{opt} = 1.388 Å), respectively. n is the number of C-C bonds in the analyzed ring and α = 257.7 Å^{-2} is a normalization factor that gives HOMA value of 1 for perfect aromatic benzene ring and a HOMA value of 0 for an alternating nonaromatic Kekulé cyclohexatriene ring.

The uncertainty (standard deviation) in the HOMA value (σ_{H}) was calculated from the uncertainty of the bond lengths ($\sigma_{\mathrm{R},i}$) using the equation:

$$\sigma_{\mathrm{H}} = \frac{\alpha}{n}\sqrt{\sum_{i=1}^{n}(2\sigma_{\mathrm{R},i}(R_i - R_{\mathrm{opt}})) + \sigma_{\mathrm{R},i}{}^2)^2}$$

The bond length data and calculated HOMA indexes of the central six- and eight-membered rings for **CP3**, **CP4** and **CP4**·2C_{60} are shown in the following table:

Table S2. Bond length data used for calculating HOMA values of central six- and eight-membered rings in **CP3**, **CP4** and **CP4**·2C_{60}.

molecule	bond lengths[a] (Å)	HOMA
**CP3** (crystal structure)	1.437(10), 1.437(10), 1.431(12), 1.407(14), 1.453(10), 1.388(10)[b]	0.52(11)
**CP3** (DFT, D_3 symmetry)	1.417, 1.413, 1.417, 1.414, 1.417, 1.414	0.81
**CP4** (DFT, D_{2d} symmetry)	1.466, 1.380, 1.466, 1.380, 1.466, 1.380, 1.466, 1.380	0.21
**CP4**·2C_{60} (crystal structure)	1.381(15), 1.467(15), 1.510(15), 1.359(14), 1.438(14), 1.372(14), 1.465(14), 1.356(14)	–0.02(18)
COT (crystal structure, ref 11)	1.465(3), 1.332(3), 1.472(2), 1.332(3), 1.456(3), 1.332(2), 1.469(2), 1.332(2)	–0.18(3)

[a]Bond length data of **CP3** and **CP4·2C60** are from their single crystal structures and the bond length data of **CP4** are from DFT optimized geometry; bond lengths for COT come from ref. 11.
[b]Bond lengths listed here for the **CP3** crystal structure are for the major component. The corresponding values for the minor component are: 1.339(15), 1.438(19), 1.41(2), 1.418(17), 1.43(2), 1.497(19).

UV-vis, Fluorescence and ^{1}H NMR Binding Study of CP4 with Fullerenes

Typical procedure for UV-vis titrations: Fullerene ($c = 1.65 \times 10^{-3}$ M for C_{60} and 3.34×10^{-4} M for C_{70}) dissolved in a toluene solution containing **CP4** ($c = 1.65 \times 10^{-6}$ M) was added to the toluene solution of **CP4** ($c = 1.65 \times 10^{-6}$ M) and the UV-vis absorption spectra were recorded at 298 K. The change in the absorbance of **CP4** caused by addition of C_{60} was calculated by subtracting the absorption intensity at 421 nm to 605 nm, at which wavelengths C_{60} has the same absorbance. For C_{70}, the absorption intensity at 421 nm was subtracted to that at 518 nm to calculate the change of UV-vis absorption caused by adding C_{70}. Binding curves were obtained by plotting $y = A_{421\,\mathrm{nm}} - A_{605\,\mathrm{nm}}$ or $A_{421\,\mathrm{nm}} - A_{518\,\mathrm{nm}}$ against the concentration of C_{60} and C_{70}. Association constants K_a were evaluated by applying a nonlinear curve fitting of y observed for **CP4** upon titration with C_{60} or C_{70} using the following equation:

$$y = y_{max} \times ((1 + K_a \times x + K_a \times H) - ((1 + K_a \times x + K_a \times H)^2 - 4 \times K_a^2 \times H \times x)^{0.5})/(2 \times K_a \times H) + A$$

where, y_{max} indicates the maximum change of UV-vis absorption intensity at complete complexation of **CP4**, K_a, x and H indicate the binding constant, concentration of fullerenes and concentration of binding sites of host **CP4**, respectively. A is a constant.

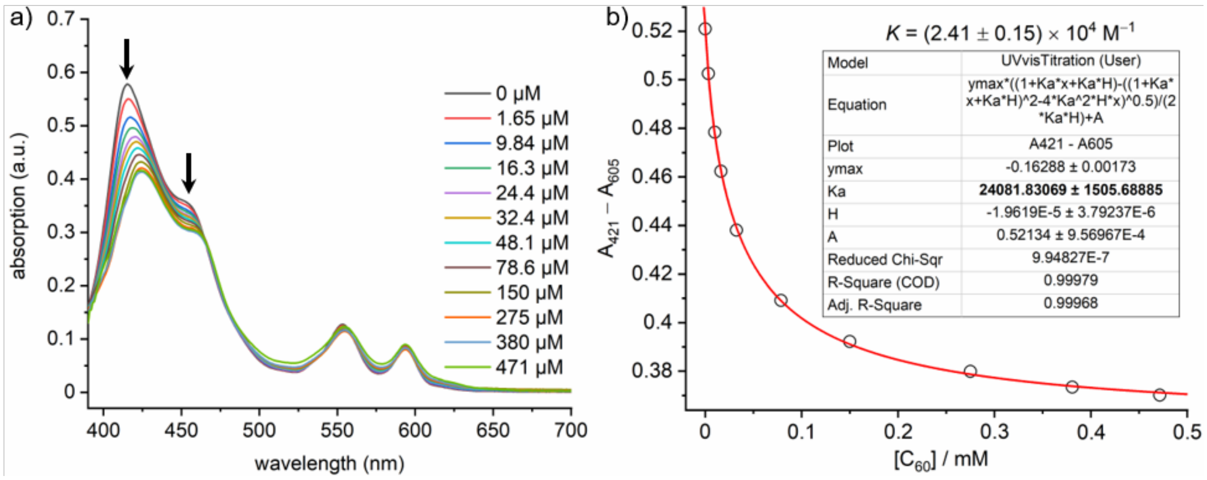

Figure S11. UV-vis absorption spectra change of the solution of **CP4** upon addition of C_{60} at room temperature. The spectra were corrected by subtracting C_{60} absorption background. Inset shows change of ($A_{421\,\mathrm{nm}} - A_{605\,\mathrm{nm}}$) with the addition of C_{60} and the red line is the fitting curve using the 1:1 binding equation.

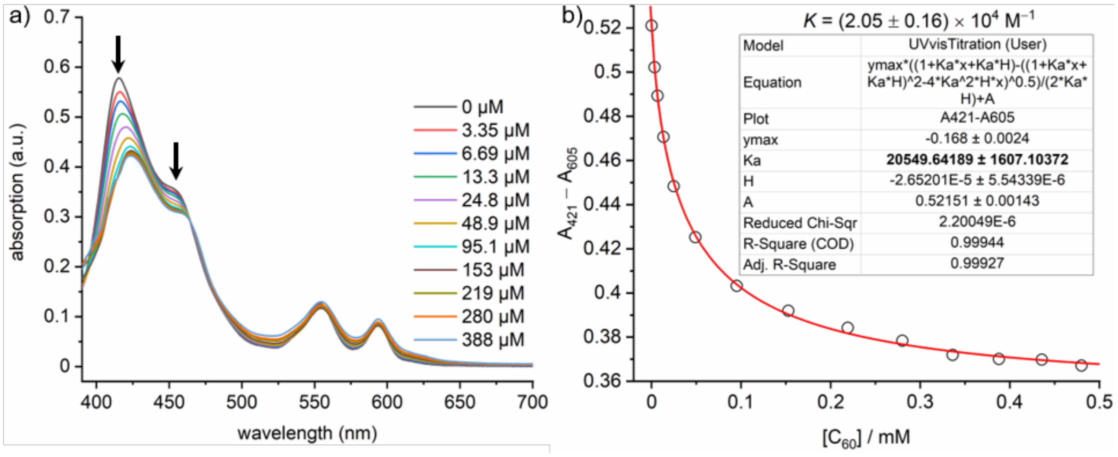

Figure S12. UV-vis absorption spectra change of the solution of **CP4** upon addition of C_{60} at room temperature. The spectra were corrected by subtracting C_{60} absorption background. Inset shows change of ($A_{421\,\mathrm{nm}} - A_{605\,\mathrm{nm}}$) with the addition of equivalents of C_{60} and the red line is the fitting curve using the 1:1 binding equation.

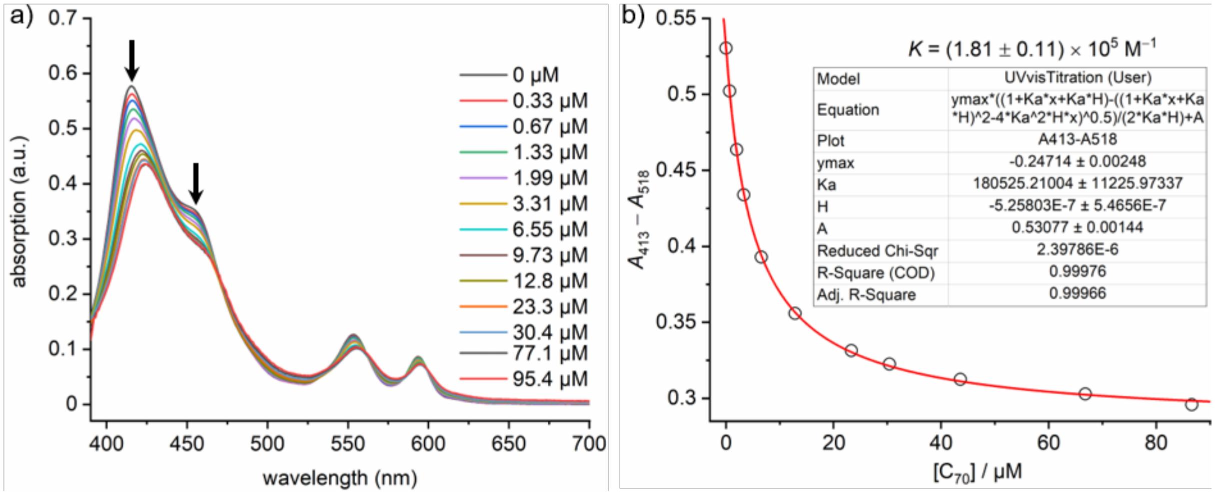

Figure S13. UV-vis absorption spectra change of the solution of **CP4** upon addition of C_{70} at room temperature. The spectra were corrected by subtracting C_{70} absorption background. Inset shows change of ($A_{413\ nm} - A_{518\ nm}$) with the addition of C_{70} and the red line is the fitting curve using the 1:1 binding equation.

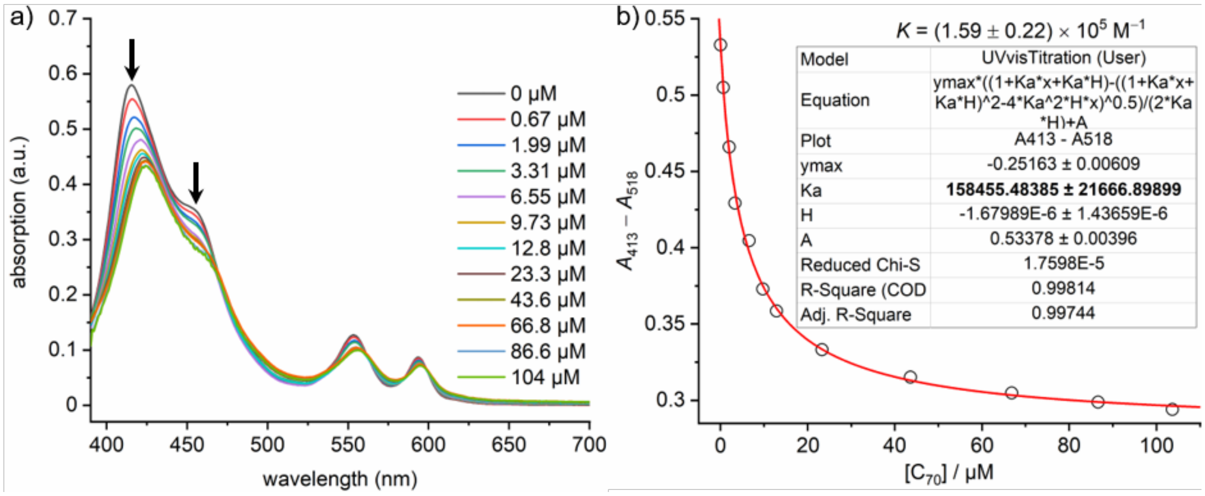

Figure S14. UV-vis absorption spectra change of the solution of **CP4** upon addition of C_{70} at room temperature. The spectra were corrected by subtracting C_{70} absorption background. Inset shows change of ($A_{413\ nm} - A_{518\ nm}$) with the addition of C_{70} and the red line is the fitting curve using the 1:1 binding equation.

Typical procedure for fluorescence titrations: Fullerene ($c = 1.68 \times 10^{-3}$ M for C_{60} and 3.34×10^{-4} M for C_{70}) dissolved in a toluene solution containing **CP4** ($c = 1.65 \times 10^{-7}$ M) was added to the toluene solution of **CP4** ($c = 1.65 \times 10^{-7}$ M) and the fluorescence spectra were recorded at 298 K with excitation wavelength of 415 nm. Binding curves were obtained by plotting the fluorescence intensity at 600 nm against the concentration of fullerene. Association constants K_a were evaluated by applying a nonlinear curve fitting of y observed for **CP4** upon titration with C_{60} or C_{70} using the flowing equation:

$$y = y_{max} \times ((1 + K_a \times x + K_a \times H) - ((1 + K_a \times x + K_a \times H)^2 - 4 \times K_a^2 \times H \times x)^{0.5})/(2 \times K_a \times H) + A$$

where, y_{max} indicates the maximum change of fluorescence intensity at complete complexation of **CP4** with fullerene, K_a, x and H indicates the binding constant, concentration of fullerenes and concentration of binding sites of **CP4**, respectively. A is a constant. The average binding constants of two independent measurements are $(1.13 \pm 0.02) \times 10^5$ M^{-1} and $(7.83 \pm 0.06) \times 10^5$ M^{-1} for C_{60} and C_{70}, respectively.

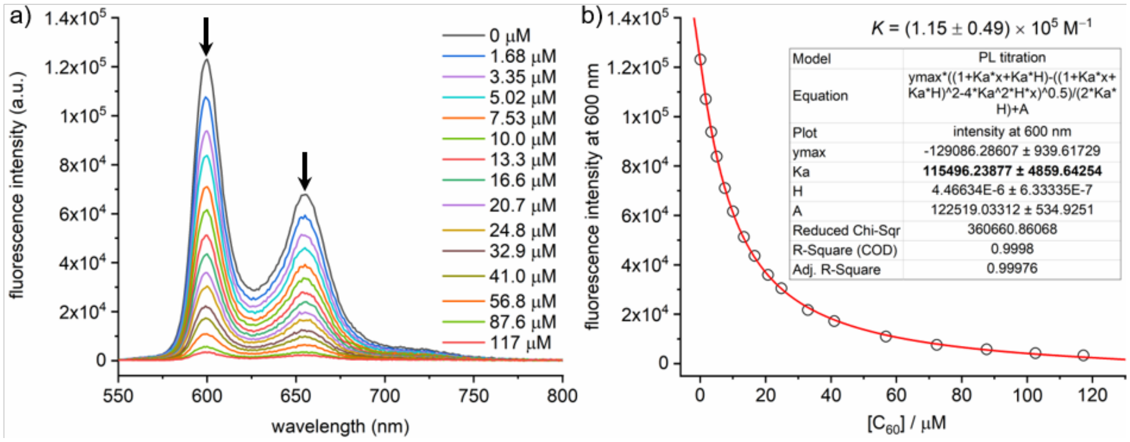

Figure S15. a) Fluorescence spectra change of **CP4** (c = 1.65 × 10^{-7} M) upon addition of C_{60} solution in toluene at 298 K (excitation wavelength = 415 nm); b) the change of fluorescence intensity at 600 nm with adding C_{60} and the red line is the fitting curve using the 1:1 binding equation.

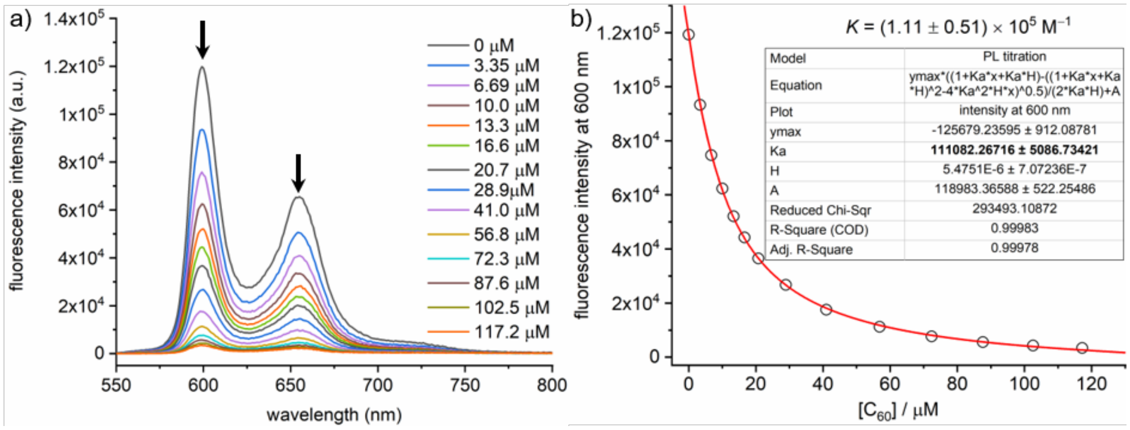

Figure S16. The second independent fluorescence titration of **CP4** with C_{60}. a) Fluorescence spectra change of **CP4** (c = 1.65 × 10^{-7} M) upon addition of C_{60} solution in toluene at room temperature (excitation wavelength = 415 nm); b) the change of fluorescence intensity at 600 nm with adding C_{60} and the red line is the fitting curve using the 1:1 binding equation.

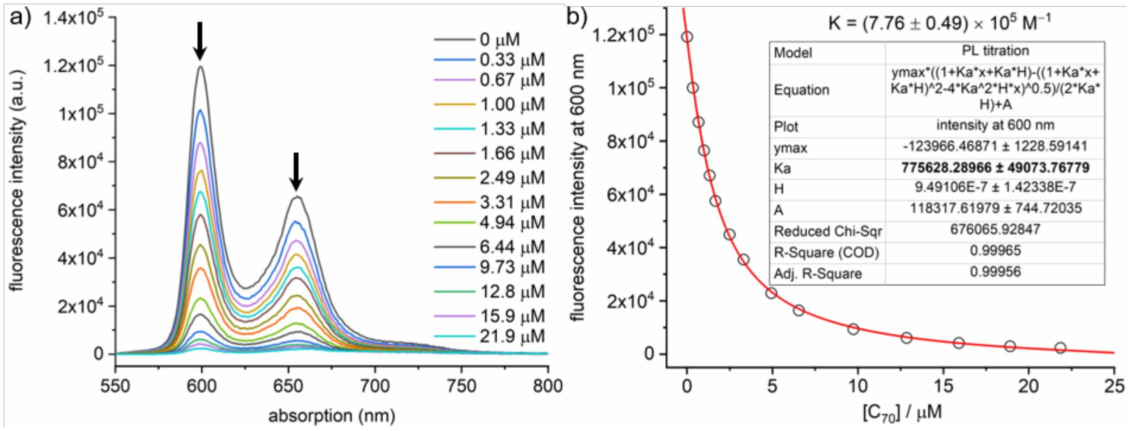

Figure S17. a) Fluorescence spectra change of **CP4** (c = 1.65 × 10^{-7} M) upon addition of C_{70} solution in toluene at room temperature (excitation wavelength = 415 nm); b) the change of fluorescence intensity at 600 nm with adding C_{70} and the red line is the fitting curve using the 1:1 binding equation.

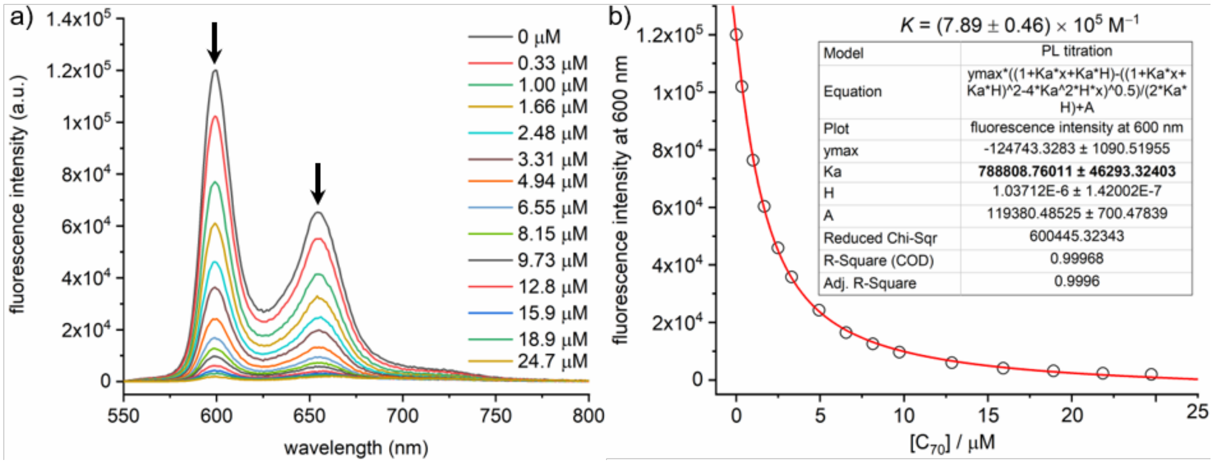

Figure S18. The second independent fluorescence titration of **CP4** with C_{70}. a) Fluorescence spectra change of **CP4** (c = 1.65 × 10^{-7} M) upon addition of C_{70} solution in toluene at room temperature (excitation wavelength = 415 nm); b) the change of fluorescence intensity at 600 nm with adding C_{70} and the red line is the fitting curve using the 1:1 binding equation.

Table S3. Summary of binding constants of **CP$_4$** with **C_{60}** and **C_{70}** measured in toluene at 25 °C (c = 1.65 × 10^{-6} M for UV-vis titration and c = 1.65 × 10^{-7} M for PL titration).

	UV-vis titration (K)		PL titration (K)	
with **C_{60}**	run 1	(2.41 ± 0.15) × 10^4 M^{-1}	run 1	(1.81 ± 0.11) × 10^5 M^{-1}
	run 2	(2.05 ± 0.16) × 10^4 M^{-1}	run 2	(1.59 ± 0.22) × 10^5 M^{-1}
	Average	(2.23 ± 0.11) × 10^4 M^{-1}	Average	(1.70 ± 0.12) × 10^5 M^{-1}
with **C_{70}**	run 1	(1.15 ± 0.49) × 10^5 M^{-1}	run 1	(7.76 ± 0.49) × 10^5 M^{-1}
	run 2	(1.11 ± 0.51) × 10^5 M^{-1}	run 2	(7.89 ± 0.46) × 10^5 M^{-1}
	Average	(1.13 ± 0.35) × 10^5 M^{-1}	Average	(7.83 ± 0.34) × 10^5 M^{-1}

Typical procedure for ^{1}H NMR titrations: Equivalents of fullerene dissolved in toluene-d_8 was added to a solution of **CP4** in toluene-d_8 (0.7 mL) and the excess solvent was evaporated to sustain the whole solvent volume of 0.7 mL. The resulting solution was subjected to ^{1}H NMR spectroscopy (400 MHz) measurement at 298 K. Binding curves were obtained by plotting the chemical shift of protons (y) against the concentration of fullerene. Association constants K_a were evaluated by applying a nonlinear curve fitting of y observed for **CP4** upon titration with C_{60} or C_{70} using the flowing equation:

$$y = y_{max} \times ((1 + K_a \times x + K_a \times H) - ((1 + K_a \times x + K_a \times H)^2 - 4 \times K_a^2 \times H \times x)^{0.5})/(2 \times K_a \times H) + A$$

where, y_{max} indicates the maximum change of proton chemical shift at complete complexation of **CP4** with fullerene, K_a, x and H indicates the binding constant, concentration of fullerenes and concentration of binding sites of **CP4**, respectively. A is a constant.

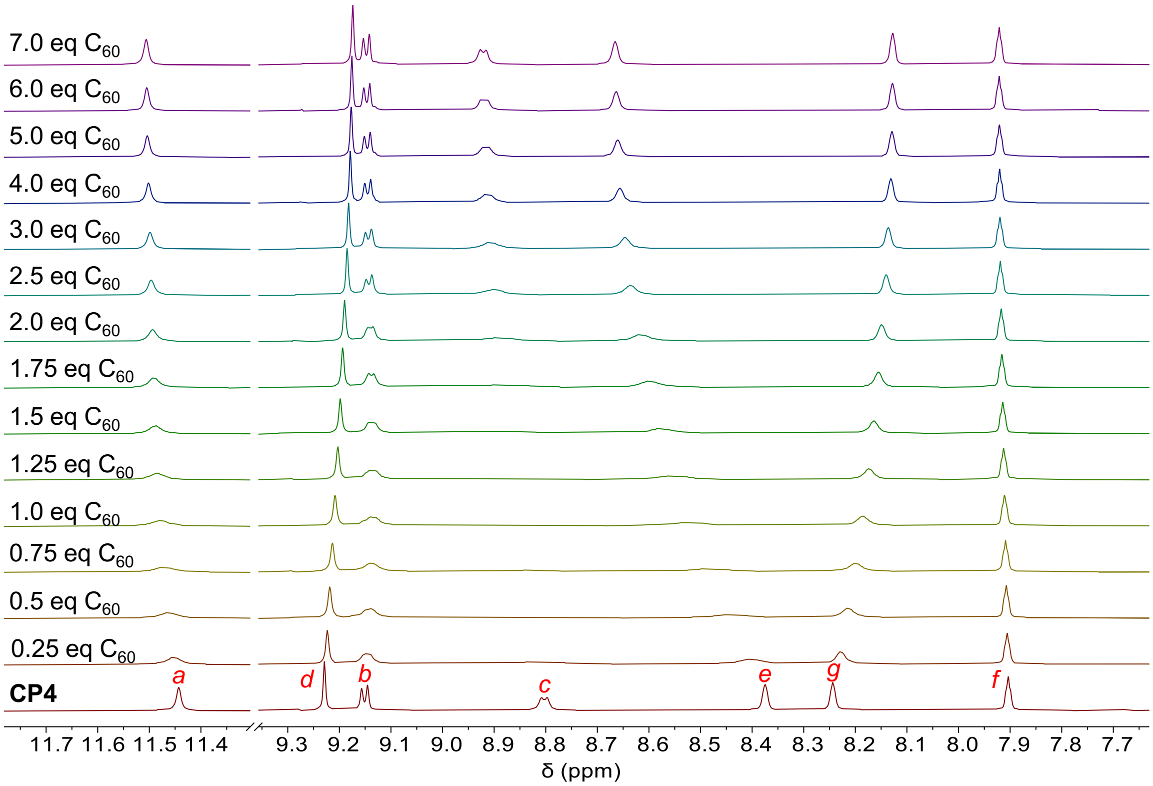

Figure S19. ^{1}H NMR spectra change of **CP4** upon addition of C_{60} in toluene-d_8 at 298 K (400 MHz).

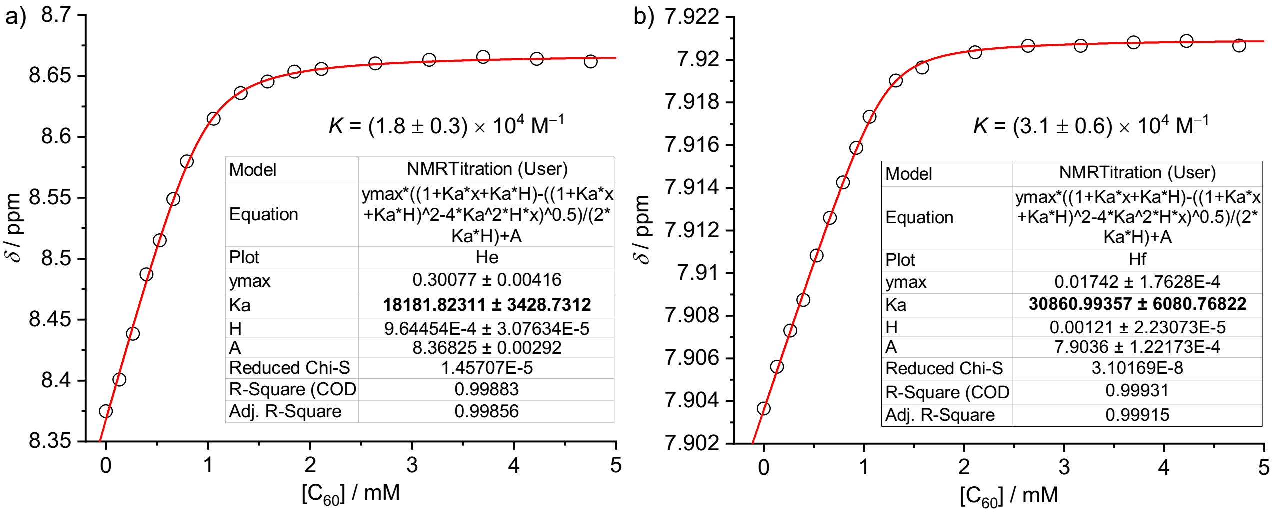

Figure S20. Plots of chemical shift of a) H^e and b) H^f on **CP4** ($c = 0.527$ mM) upon addition of C_{60} measured in toluene-d_8 at 298 K (400 MHz). The red line indicates corresponding fitted curve using the 1:1 equation. The average binding constant measured by ^{1}H NMR is $(2.5 \pm 0.7) \times 10^4$ M^{-1}. The average host concentration is 1.09 ± 0.02 mM, which is right twice the concentration of **CP4**, indicating every **CP4** binds two C_{60} molecules in the complex.

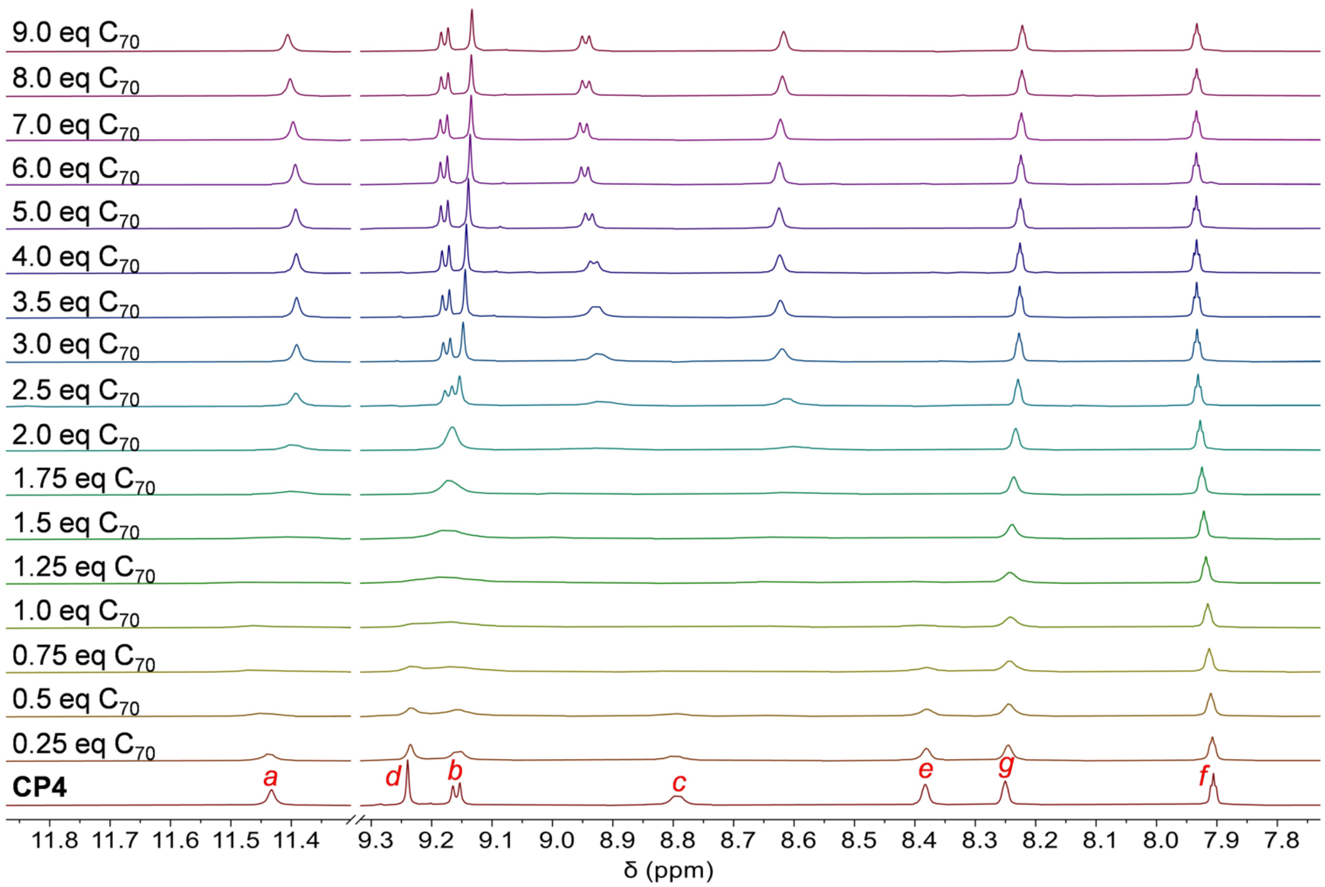

Figure S21. ^{1}H NMR spectra change of **CP4** upon addition of C_{70} in toluene-d_8 at 298 K (400 MHz).

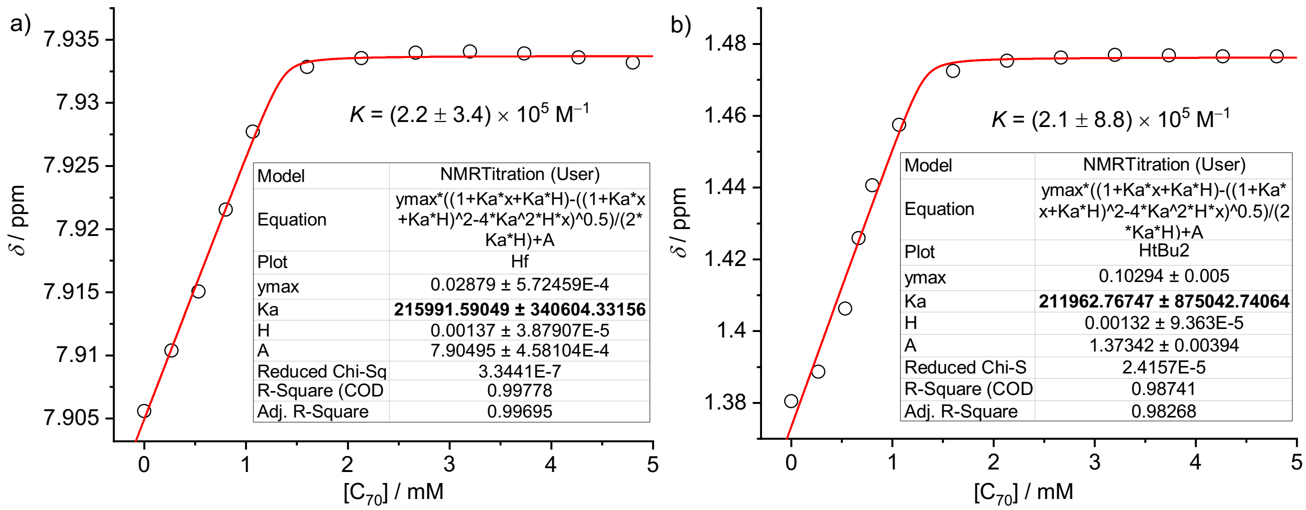

Figure S22. Plots of chemical shift of a) H^f and b) H^{Bu} on **CP4** (c = 0.616 mM) upon addition of C_{70} measured in toluene-d_8 at 298 K (400 MHz). The red line indicates corresponding fitted curve using the 1:1 equation. The average binding constant measured by ^{1}H NMR is (2.1 ± 0.1) × 10^5 M^{-1}. The average concentration of host determined by ^{1}H NMR is 1.35 ± 0.06 mM, which is right twice the concentration of **CP4**, indicating each **CP4** binds two C_{70} molecules in the complex.

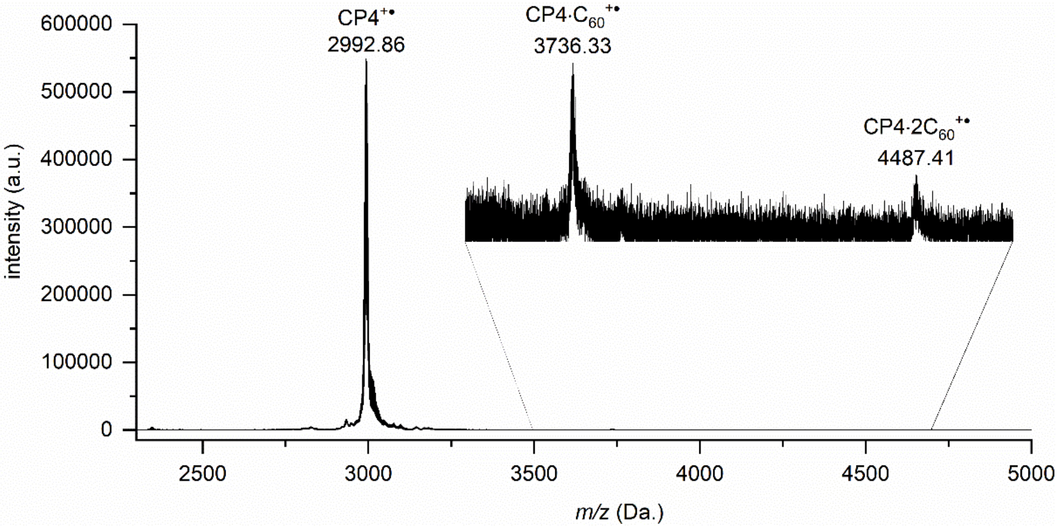

Figure S23. MALDI-TOF MS spectrum of the mixture of **CP4** and C_{60} (DCTB in tetrahydrofuran was used as matrix).

X-ray Crystallographic Analysis

Single crystal X-ray diffraction data for structure **1** were collected using a (Rigaku) Oxford Diffraction SuperNova A diffractometer and reduced using CrysAlisPro. The structure was solved using SuperFlip[12] and refined using CRYSTALS[13,14] as detailed in the CIF.

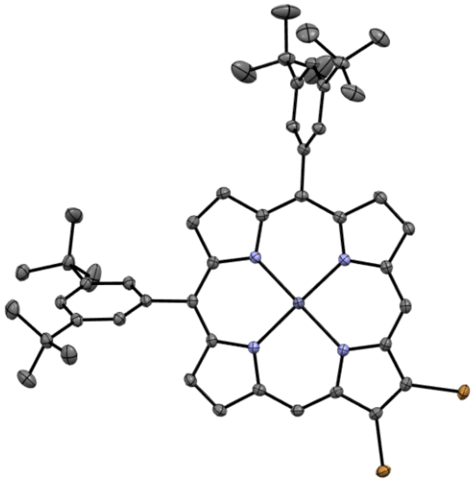

Figure S24. X-ray Single-crystal structure of **1** (Hydrogen atoms and solvent molecules were omitted for clarity). The thermal ellipsoids are 50% probability level.

Table S4. Crystal data and structural refinement for **1**.

Compound	2253581
Formula	$C_{48}H_{50}Br_2N_4Zn$, C_6H_6
$D_{calc.}$/ g cm^{-3}	1.402
μ/mm^{-1}	3.037
Formula Weight	986.25
Color	clear intense red
Shape	plate
Size/mm^3	0.03×0.08×0.10
T/K	150(2)
Crystal System	triclinic
Space Group	$P\bar{1}$
a/Å	10.6547(2)
b/Å	15.0715(3)
c/Å	15.1256(2)
α/°	95.5583(14)
β/°	94.9031(14)
γ/°	103.5286(15)
V/Å^3	2335.69(7)
Z	2
Z'	1
Wavelength/Å	1.54184

Radiation type	Cu K$_{\alpha}$
Θ_{min}/°	3.038
Θ_{max}/°	76.261
Measured Reflections.	57124
Independent Reflections	9690
Reflections $I \geq 2\ \sigma(I)$	8698
R_{int}	0.035
Parameters	606
Restraints	96
Largest Peak	0.58
Deepest Hole	-0.37
GooF	0.9903
wR_2 (all data)	0.0630
wR_2	0.0657
R_1 (all data)	0.0244
R_1	0.0280

Single black block-shaped crystals of **CP3** (2223433) were grown from a mixture of chlorobenzene and methanol. A suitable crystal with dimensions 0.190 × 0.160 × 0.100 mm^3 was selected and mounted on a MITIGEN holder in oil on a Rigaku 007HF diffractometer equipped with HF Varimax confocal mirrors, an AFC11 goniometer and HyPix 6000HE detector. The crystal was kept at a steady T = 100(2) K during data collection. The structure was solved with the **ShelXT** 2018/2 (ref. 15) solution program using dual methods and by using **Olex2** 1.5 (ref. 16) as the graphical interface. The model was refined with **ShelXL** 2018/3 (ref. 17) using full matrix least squares minimization on F^2.

The crystal used was modulated, but sufficient information could be obtained from using the standard cell and a disordered structure along with various restraints (DFIX, DANG, FLAT, SADI, BUMP, SIMU, RIGU). Solvent masking was employed, suggesting nine solvent methanol molecules per asymmetric unit.

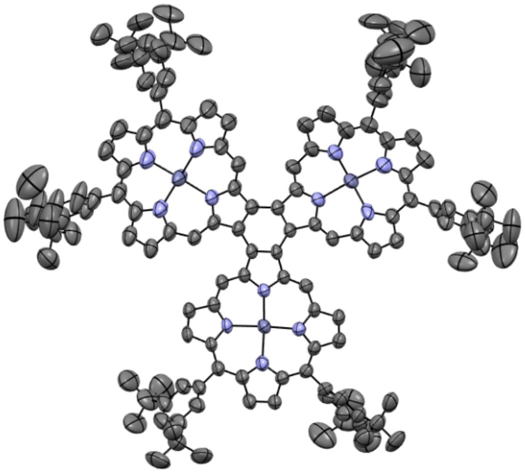

Figure S25. X-ray Single-crystal structure of **CP3** (hydrogen atoms and solvent molecules omitted for clarity). The thermal ellipsoids are 50% probability level.

Table S5. Crystal data and structural refinement for **CP3**.

Compound	CCDC 2223433
Formula	$C_{156}H_{198}N_{12}O_{12}Zn_3$
$D_{calc.}$/ g cm^{-3}	1.101
μ/mm^{-1}	0.977
Formula Weight	2629.36
Color	black
Shape	block-shaped
Size/mm^3	0.190×0.160×0.100
T/K	100(2)
Crystal System	monoclinic
Space Group	$I2/a$
a/Å	24.7570(2)
b/Å	36.7666(2)
c/Å	34.8419(2)
α/°	90
β/°	90.6050(10)
γ/°	90
V/Å^3	31712.4(4)
Z	8
Z'	1
Wavelength/Å	1.54178
Radiation type	Cu K$_\alpha$
Θ_{min}/°	3.245
Θ_{max}/°	68.244
Measured Reflections.	318676
Independent Reflections	28915
Reflections $I \geq 2\ \sigma(I)$	21506
R_{int}	0.0289
Parameters	2840
Restraints	9426
Largest Peak	0.888
Deepest Hole	-0.320
GooF	1.748
wR_2 (all data)	0.3981
wR_2	0.3765
R_1 (all data)	0.1292
R_1	0.1158

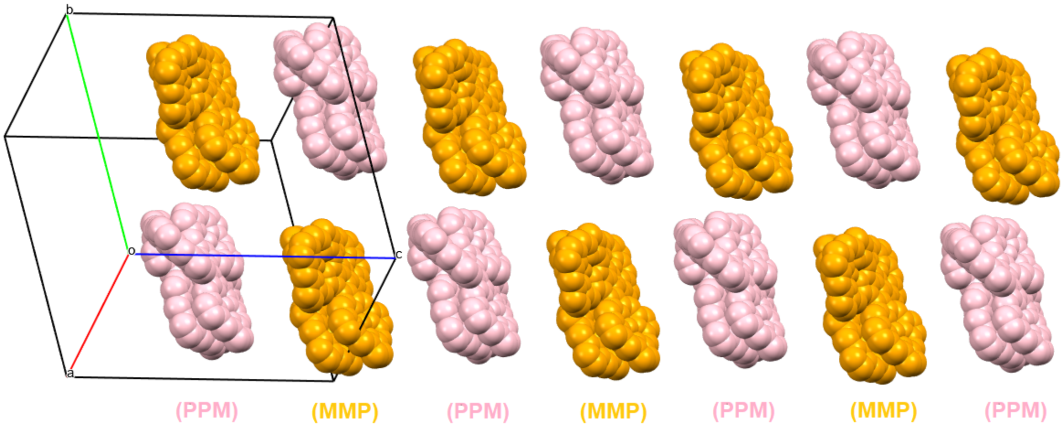

Figure S26. Packing structure of **CP3** in space-filling view, PPM and MMP enantiomers are shown in pink and orange color, solvent molecules and 3,5-di-*tert*-butylphenyl groups are omitted for clarity.

Data for crystals of **CP4**·2C_{60} were initially collected using synchrotron radiation at Diamond Light Source[18] and processed using the XIA2 software.[19] The data for this structure (included herein as **CP4**·2C_{60}-II) were of poor quality, and exhibited a structure with two **CP4** molecules and four fullerenes in the asymmetric unit. The structure solved well using SuperFlip,[12] but the refinement, carried out on F^2 within the CRYSTALS suite[13,14,20] was poor due to the quality of the data, which was suboptimal partly as a result of radiation damage, but also because of the complexity of the problem.

Examination of the structure suggested the presence of pseudo symmetry which was thought to potentially be caused by a phase transition. For this reason, the sample was re-examined to see if the presence of a phase transition could be confirmed and, whether data collected on a high temperature phase would be better.

Despite numerous attempts, the original triclinic phase (29.7891(4) Å, 30.5207(4) Å, 34.2632(5) Å, 108.632(1)°, 114.753(1)°, 96.225(1)°, V = 25723.0(7) Å^3, 2253583) was not seen again, instead a new orthorhombic phase was repeatedly found. Data were collected on this new phase using a high intensity rotating anode instrument and the best data and results are presented here.

In all cases, the structure solved readily, however in the main cyclic porphyrin oligomer there were prolate ellipsoids associated with the tertiary butyl groups, indicative of disorder. This was modelled using a split-site model with same-distance, thermal similarity and vibrational restraints to ensure the distances, angles and displacements remained sensible. This made a relatively marginal improvement to the refinement statistics so attention turned to the void which was found to include diffuse residual electron density believed to be due to disordered solvent. The application of SQUEEZE[21,22] to leave a void from which the electron density was removed improved the refinement again. However, there remained a considerable amount of residual electron density around the fullerenes. This coupled with the need for extensive restraints to maintain the geometry and large displacement ellipsoids was strongly suggestive of disorder. Initial attempts to model this suggested that more than two positions would be required and the complexity of this model was less than ideal given the already low data to parameter ratio. For this reason, a hollow sphere[23] was used coupled with the atomic model, and this improved the refinement considerably.

This hollow sphere is represented by a single atom in the center of each fullerene with an occupancy of approximately thirty. The fullerene is then completed by the atomic model where each atom has an

occupancy of approximately half. These occupancies were then appropriately weighted and refined competitively. This was implemented for both fullerenes in the orthorhombic phase, but also all four in the triclinic polymorph. In both cases, although it improved the result. Once the refinement had been stabilized, the hollow sphere was then removed and SQUEEZE used iteratively to remove the electron density from the void before reinstating the hollow spheres.

Although final structure for **CP4**·2C_{60}-I is one of poor resolution, this is likely the best currently achievable result for this material and confirms the gross structure and the 2:1 ratio of fullerene to each cyclic porphyrin oligomer, which was seen in both phases.

The result for the triclinic polymorph, **CP4**·2C_{60}-II is of even lower resolution, so are not discussed in detail though the structure is included as supplementary material for completeness. However, the results are in keeping with those seen for the better orthorhombic polymorph (**CP4**·2C_{60}-I). Together, the structures make a compelling case for the conclusions reported in the manuscript, namely the gross structure, conformation and connectivity of the **CP4** species and the 2:1 ratio of fullerene to each cyclic porphyrin oligomer.

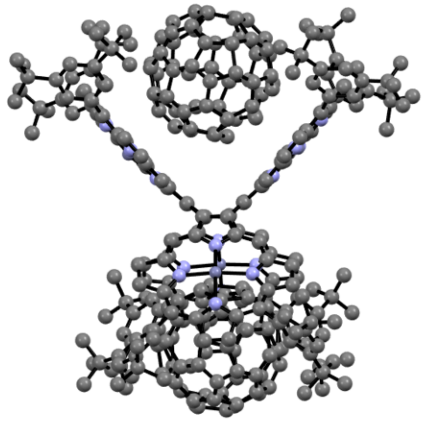

Figure S27. X-ray single crystal structure of **CP4**·2C_{60} (hydrogen atoms and solvent molecules were omitted for clarity).

Table S6. Crystal data and structural refinement for **CP4**·2C_{60}.

Compound	2253582
Formula	$C_{196}H_{218}N_{16}O_4Zn_4$, 2($C_{60}$)
$D_{calc.}$/ g cm^{-3}	1.029
μ/mm^{-1}	0.783
Formula Weight	4572.31
Color	metallic dark grey
Shape	plate
Size/mm^3	0.190×0.160×0.100
T/K	100 K
Crystal System	orthorhombic
Space Group	*Pnna*
a/Å	46.4827(1)
b/Å	62.5073(2)

c/Å	20.3205(1)
α/°	90
β/°	90
γ/°	90
V/Å^3	59041.4(4)
Z	8
Z'	1
Wavelength/Å	1.54180
Radiation type	Cu K_α
Θ_{min}/°	3.216
Θ_{max}/°	44.490
Measured Reflections.	2118059
Independent Reflections	23231
Reflections I≥2 $\sigma(I)$	17888
R_{int}	0.076
Parameters	2729
Restraints	9305
Largest Peak	1.33
Deepest Hole	-0.78
GooF	0.9879
wR_2 (all data)	0.4454
wR_2	0.4229
R_1 (all data)	0.1594
R_1	0.1478

NMR Spectra

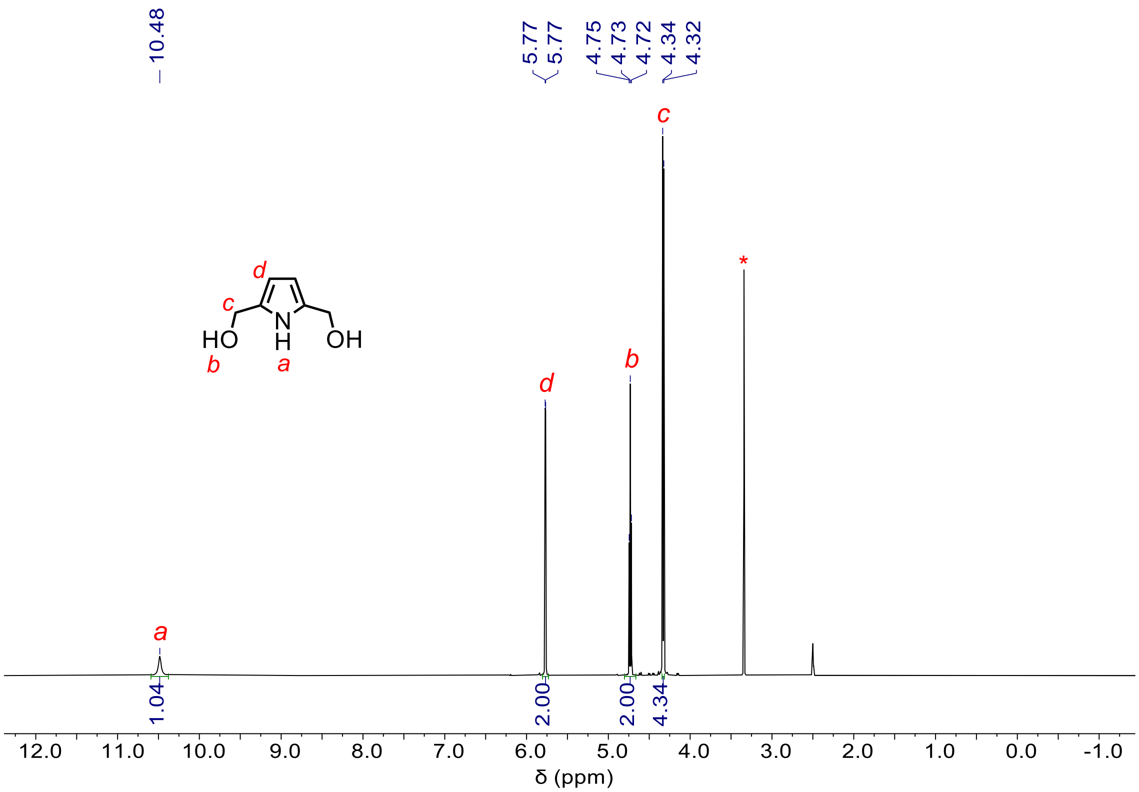

Figure S28. ^{1}H NMR spectrum of 2,5-bis(hydroxymethyl)pyrrole **2** in DMSO-d_6 (400 MHz, 298 K).
* indicates water peak from the solvent.

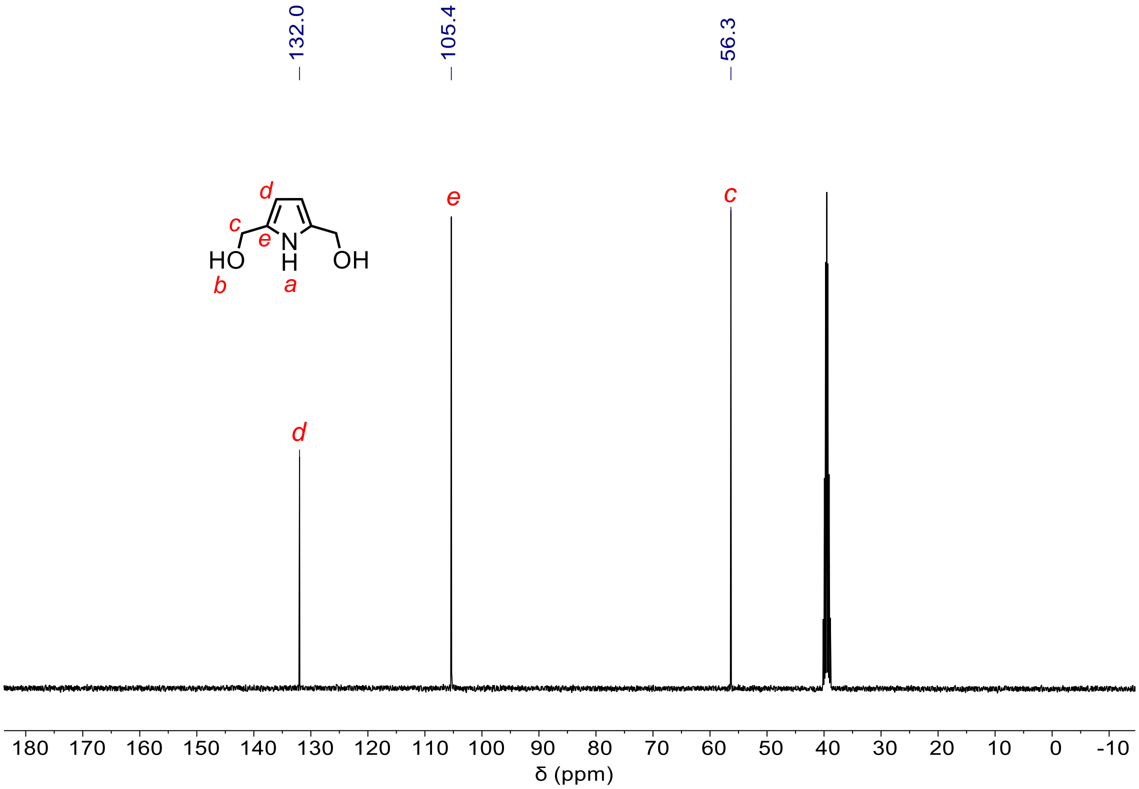

Figure S29. ^{13}C NMR spectrum of 2,5-bis(hydroxymethyl)pyrrole **2** in DMSO-d_6 (101 MHz, 298 K).

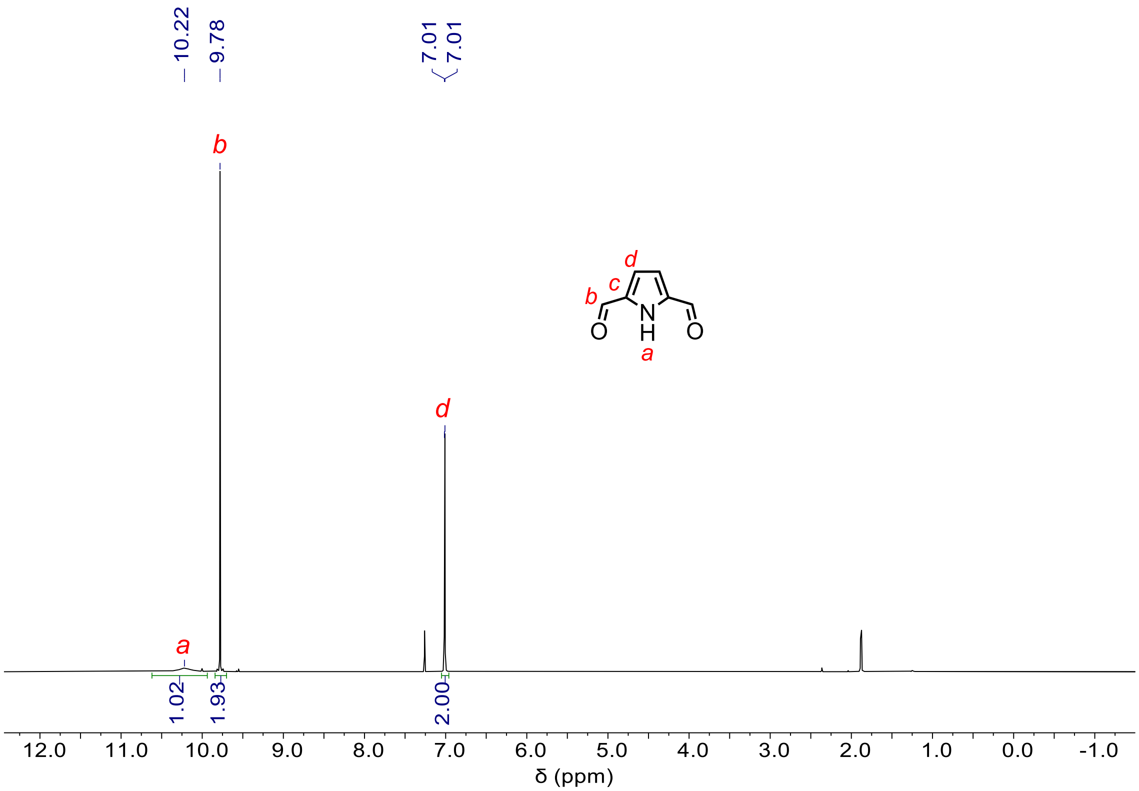

Figure S30. ^{1}H NMR spectrum of 2,5-diformylpyrrole (**3**) in $CDCl_3$ (400 MHz, 298 K).

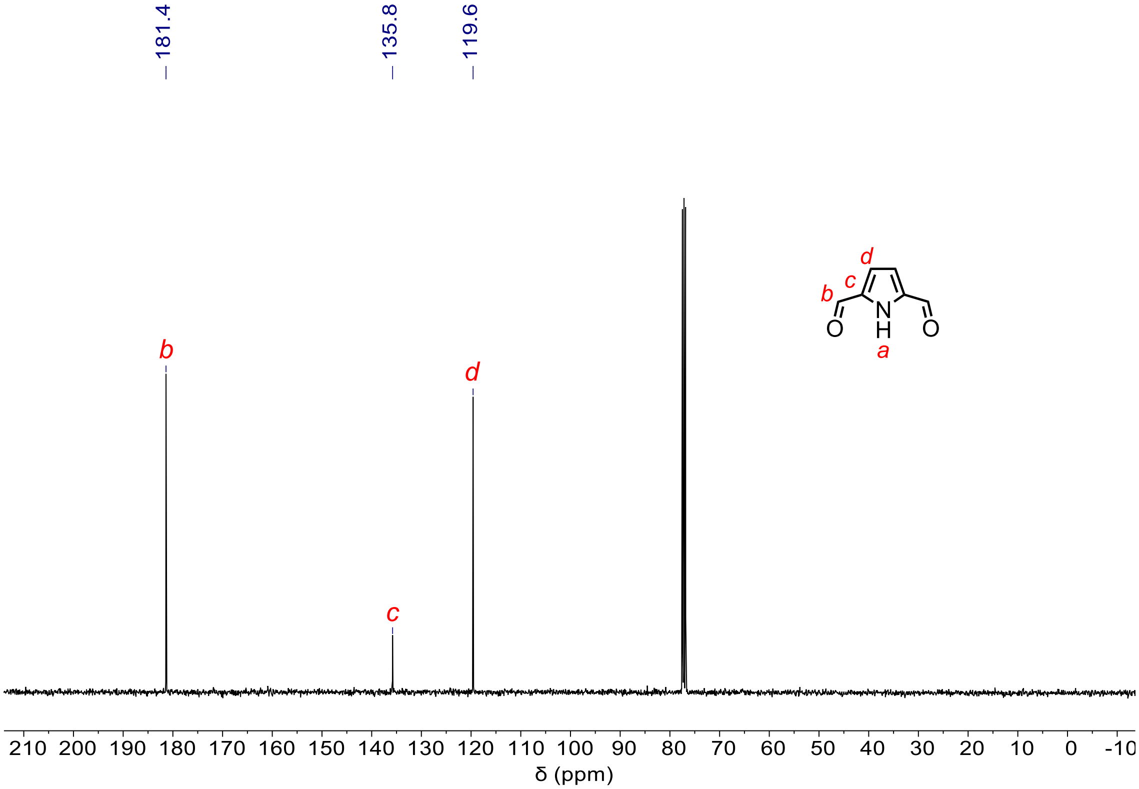

Figure 31. ^{13}C NMR spectrum of 2,5-diformylpyrrole **3** in $CDCl_3$ (101 MHz, 298 K).

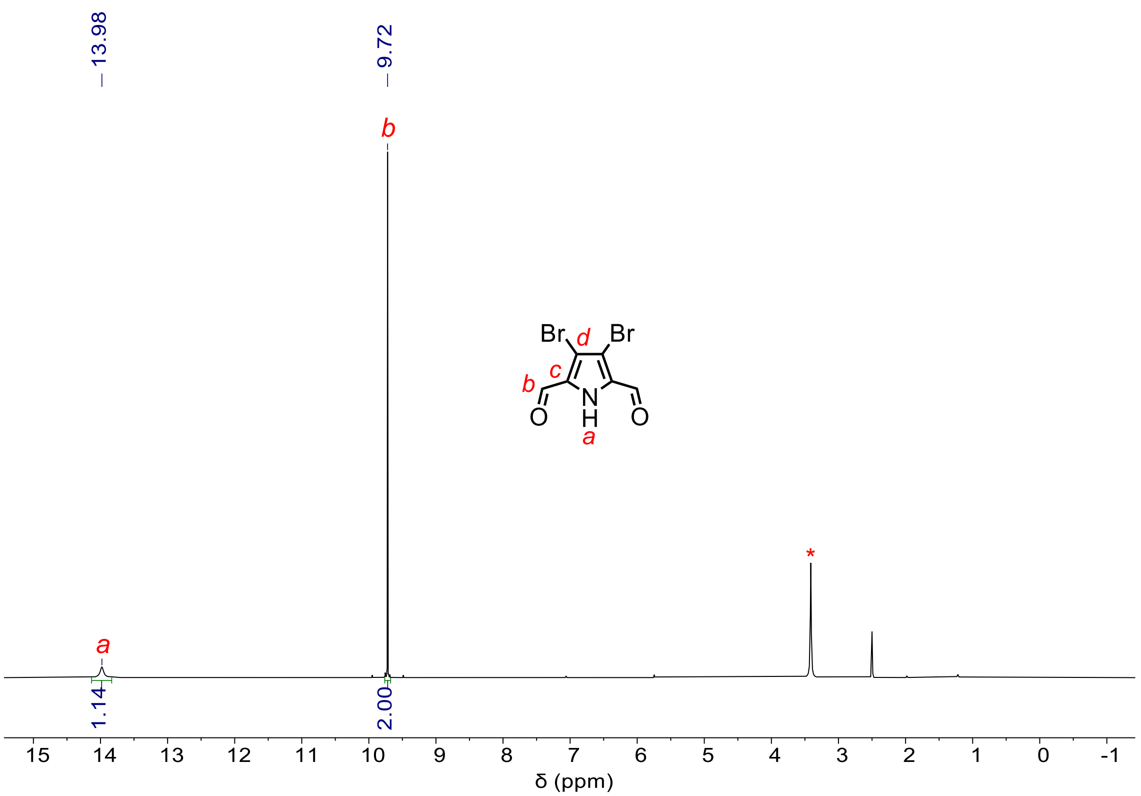

Figure S32. ^{1}H NMR spectrum of 3,4-dibromo-2,5-diformylpyrrole (**4**) in DMSO-d_6 (400 MHz, 298 K). * indicates water peak from the solvent.

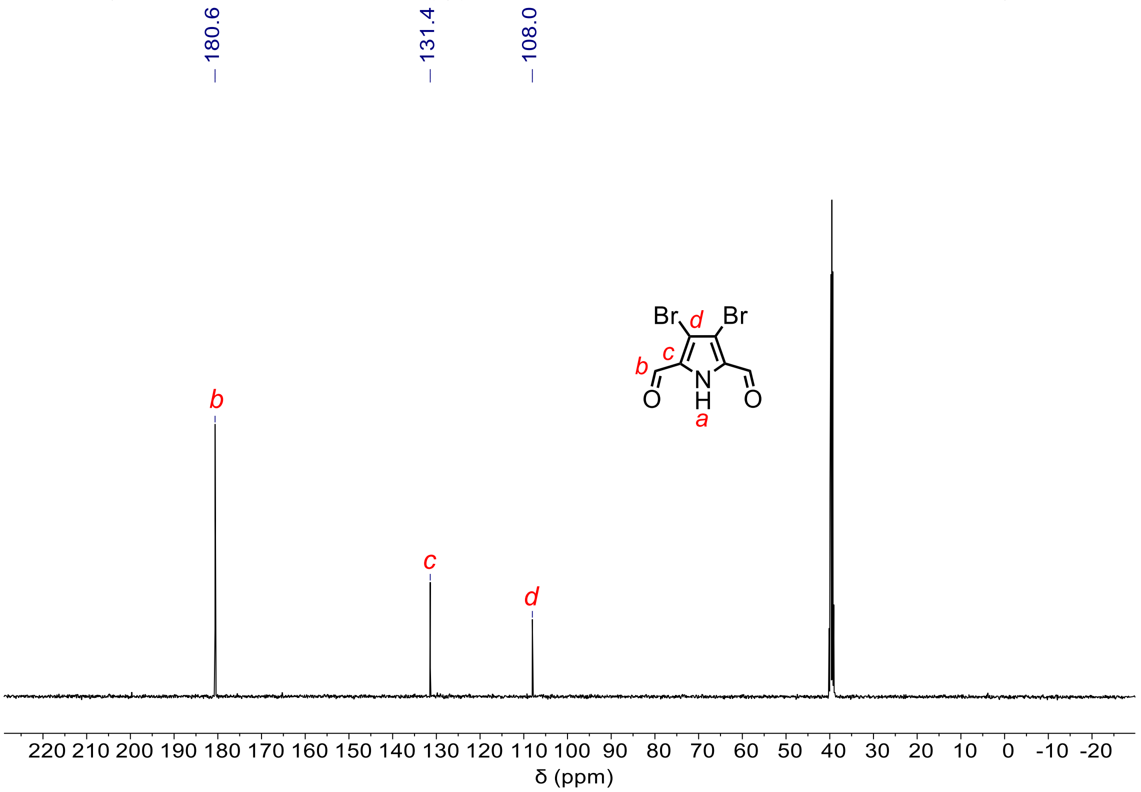

Figure S33. ^{13}C NMR spectrum of 3,4-dibromo-2,5-diformylpyrrole **4** in DMSO-d_6 (101 MHz, 298 K).

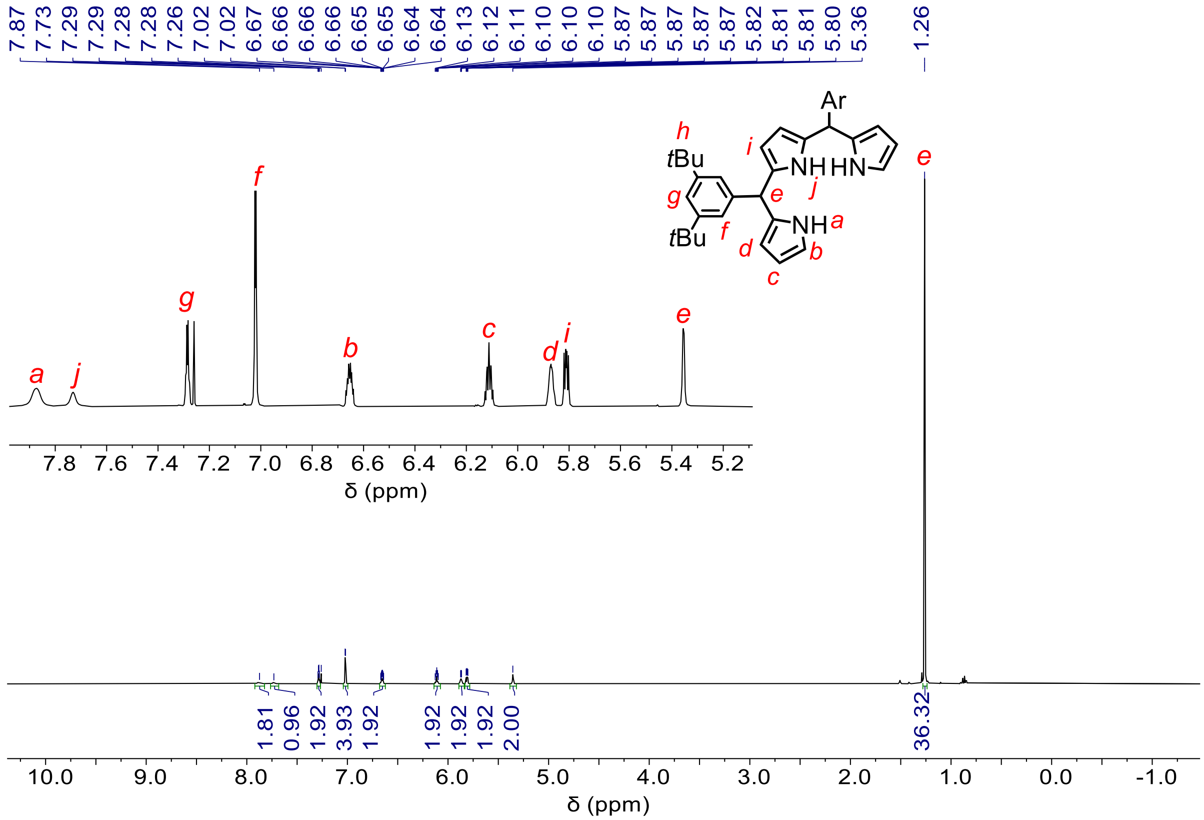

Figure S34. ^{1}H NMR spectrum of tripyrrane **5** in $CDCl_3$ (400 MHz, 298 K).

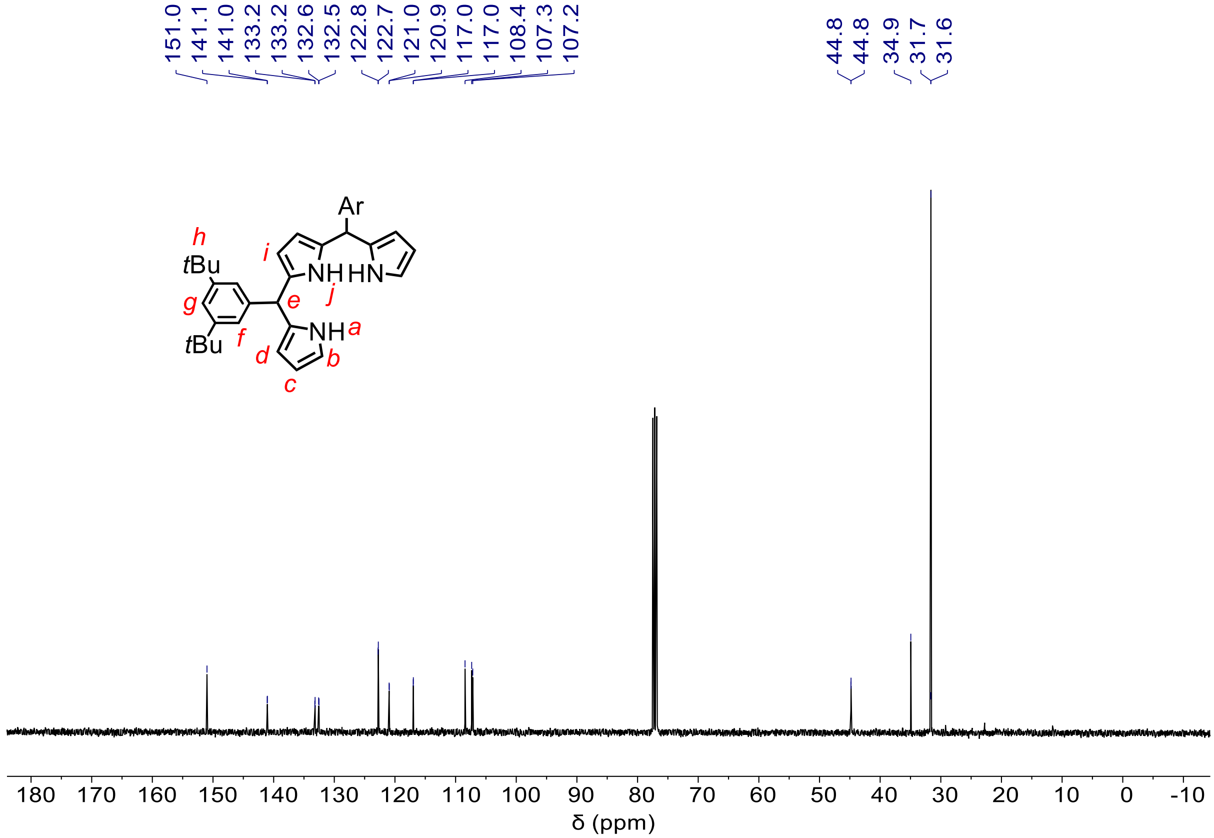

Figure 35. ^{13}C NMR spectrum of tripyrrane **5** in $CDCl_3$ (101 MHz, 298 K).

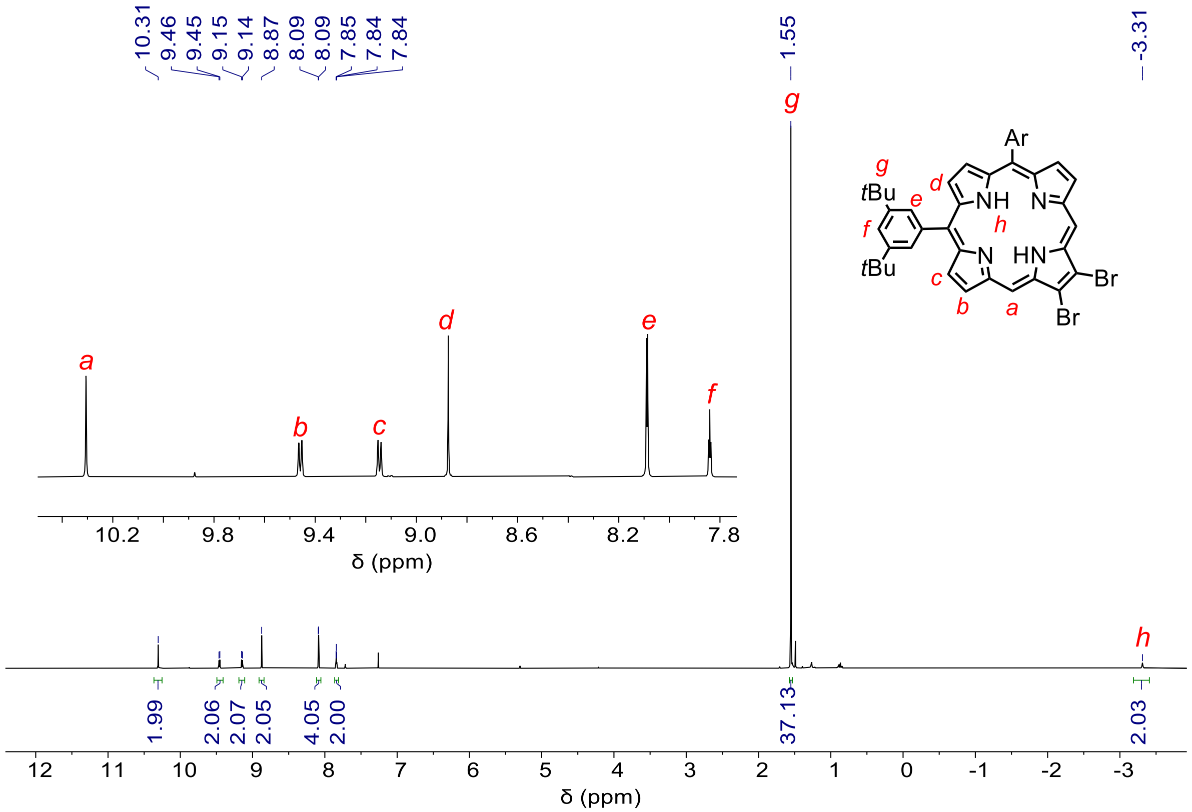

Figure 36. ^{1}H NMR spectrum of 2,3-dibromo-10,15-bis(3,5-di-*tert*-butylphenyl)porphyrin **6** in $CDCl_3$ (400 MHz, 298 K).

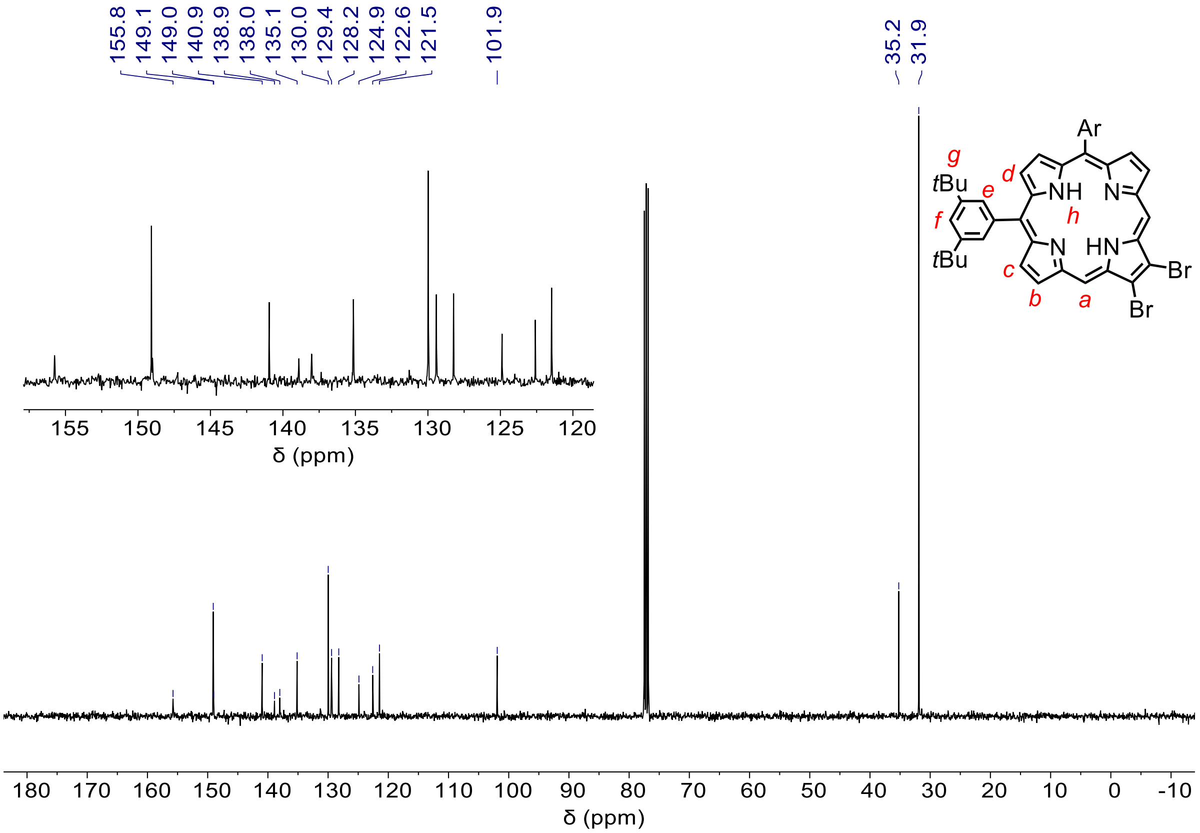

Figure S37. ^{13}C NMR spectrum of 2,3-dibromo-10,15-bis(3,5-di-*tert*-butylphenyl)porphyrin **6** in $CDCl_3$ (101 MHz, 298 K).

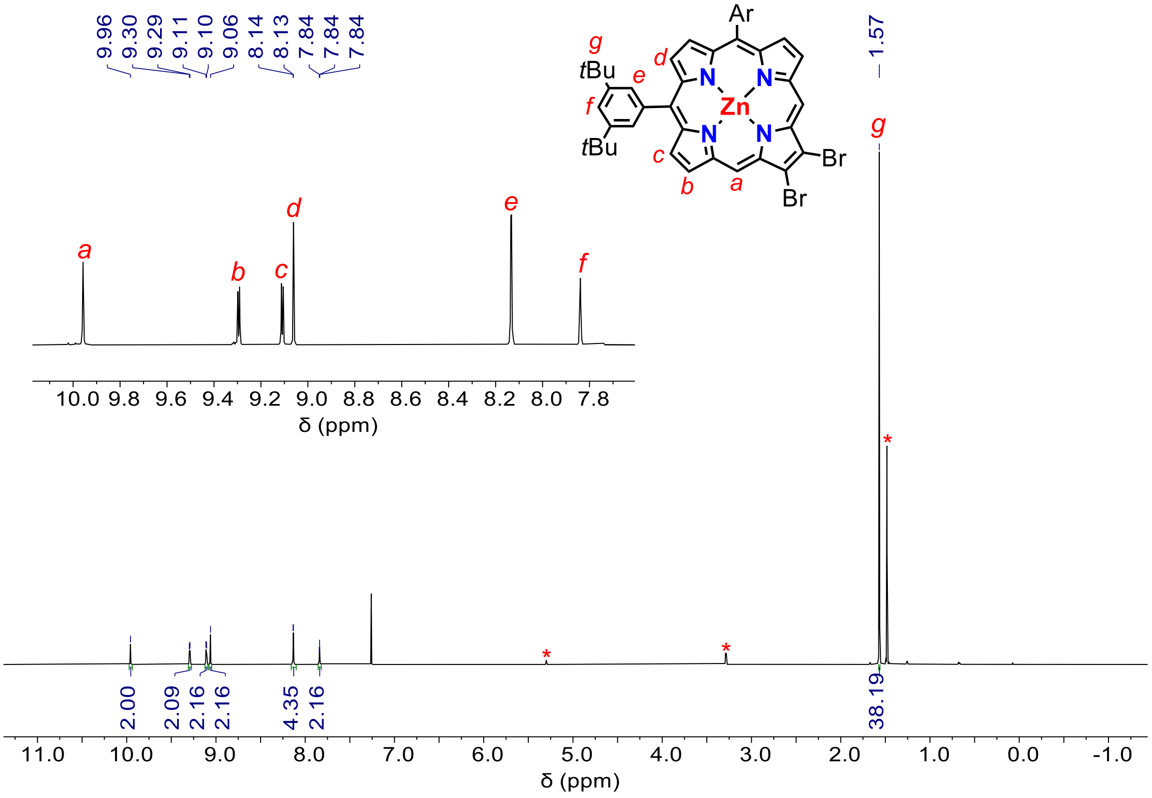

Figure S38. ^{1}H NMR spectrum of 2,3-dibromo-10,15-bis(3,5-di-*tert*-butylphenyl)porphyrin (Zn) **1** in $CDCl_3$ (600 MHz, 298 K). * indicates dichloromethane, methanol and water peak from the deuterated solvent.

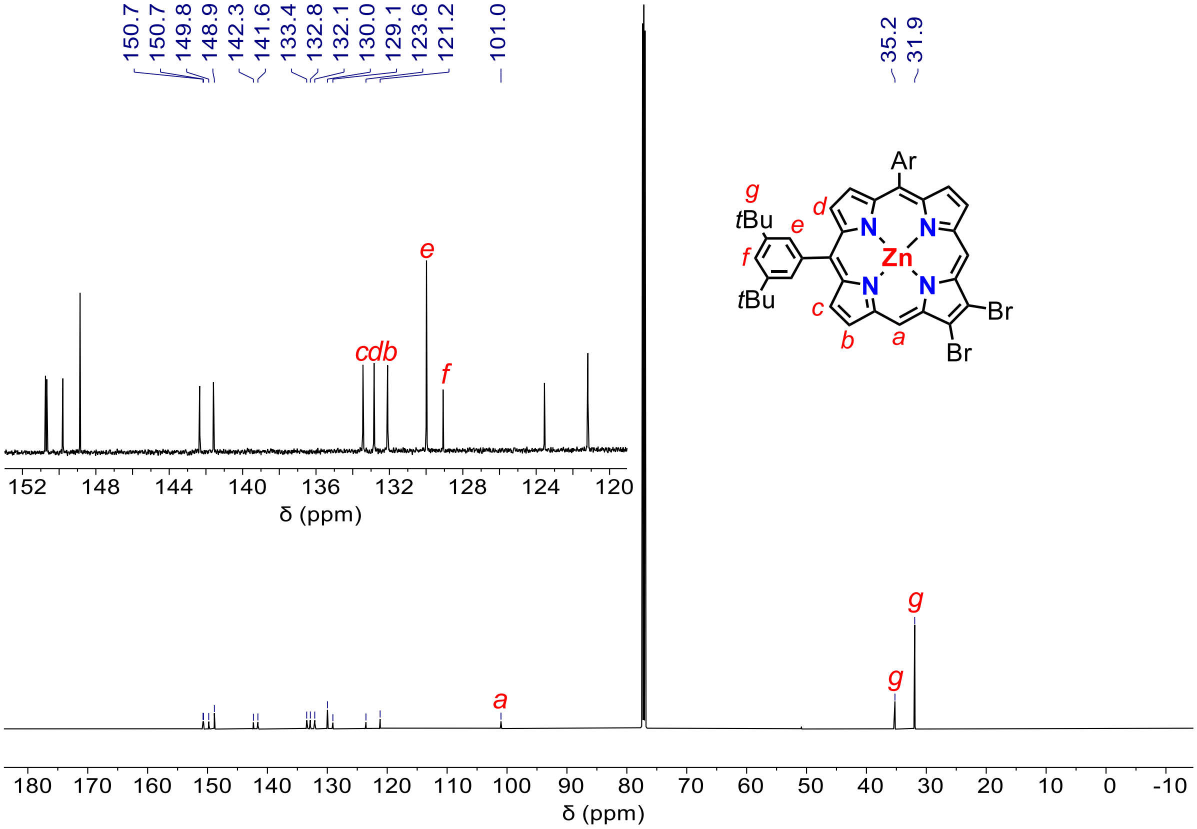

Figure S39. ^{13}C NMR spectrum of 2,3-dibromo-10,15-bis(3,5-di-*tert*-butylphenyl)porphyrin (Zn) **1** in $CDCl_3$ (151 MHz, 298 K).

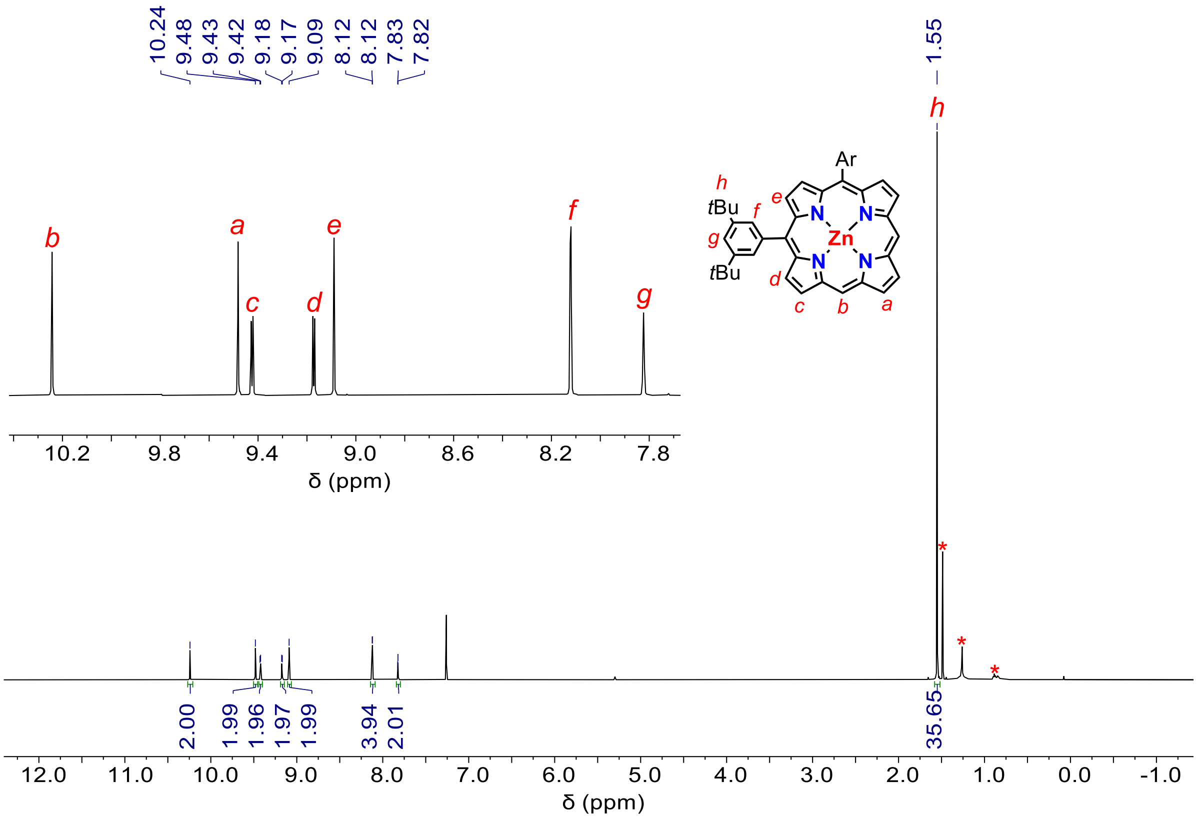

Figure S40. ^{1}H NMR spectrum of 5,10-bis(3,5-di-*tert*-butylphenyl)porphyrin (Zn) **P1** in $CDCl_3$ (600 MHz, 298 K). * indicates peak from the deuterated solvent and trace amount of grease.

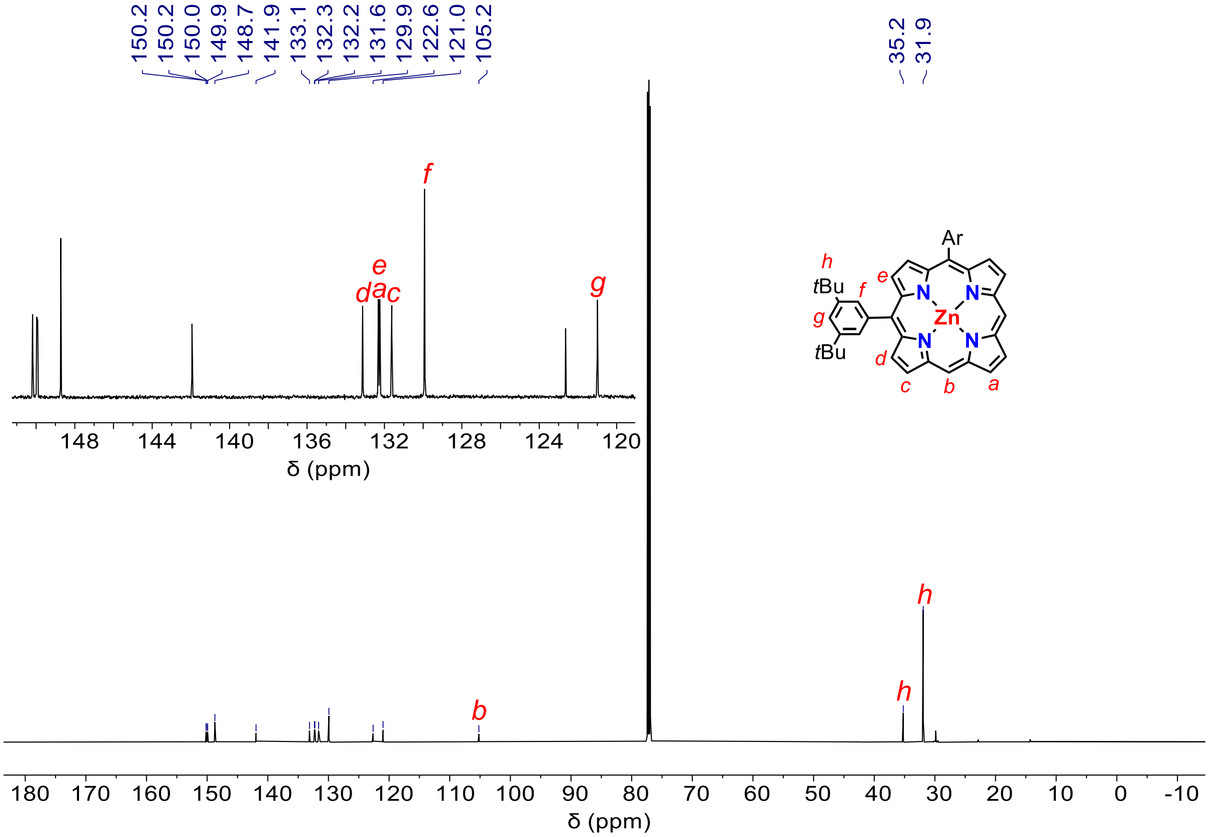

Figure S41. ^{13}C NMR spectrum of 5,10-bis(3,5-di-*tert*-butylphenyl)porphyrin (Zn) **P1** in $CDCl_3$ (151 MHz, 298 K).

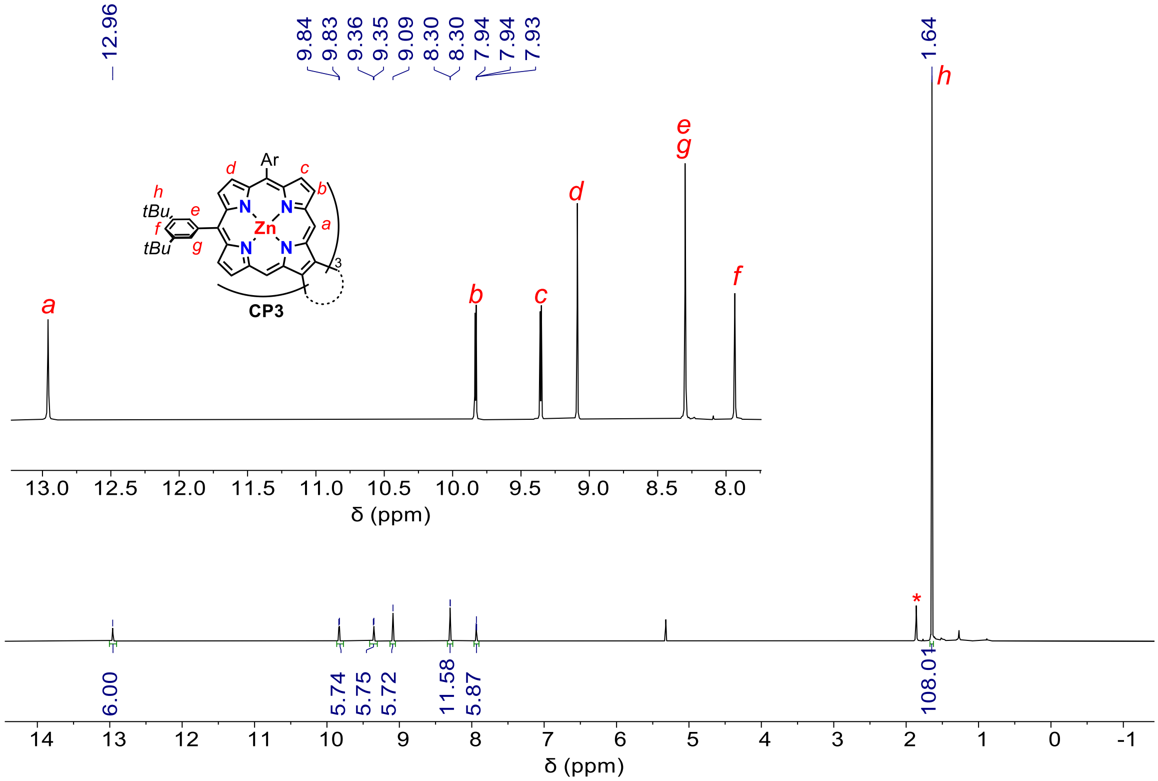

Figure S42. ^{1}H NMR spectrum of **CP3** in CD_2Cl_2 (500 MHz, 298 K). * indicates water peak from the deuterated solvent.

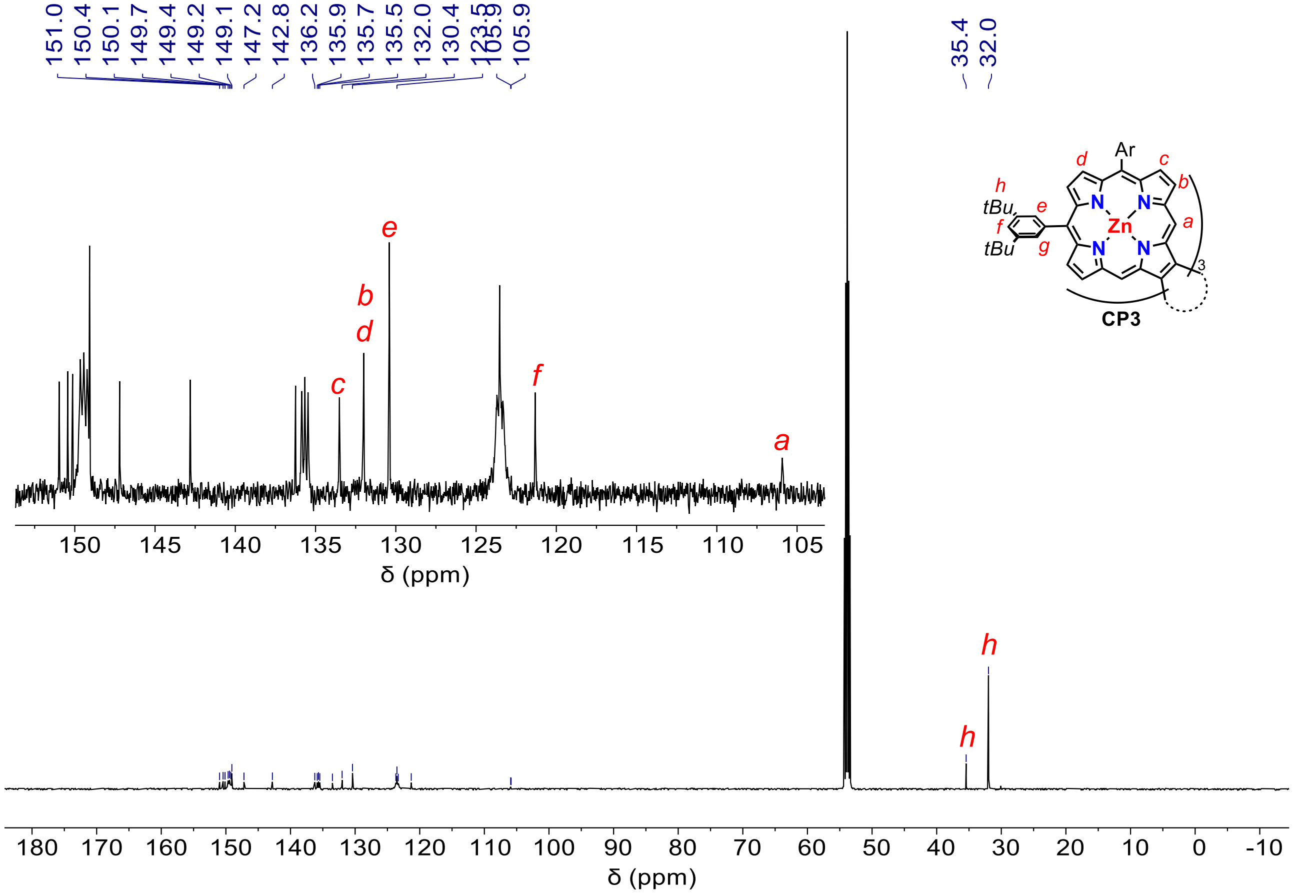

Figure S43. ^{13}C NMR spectrum of **CP3** in CD_2Cl_2 (126 MHz, 298 K).

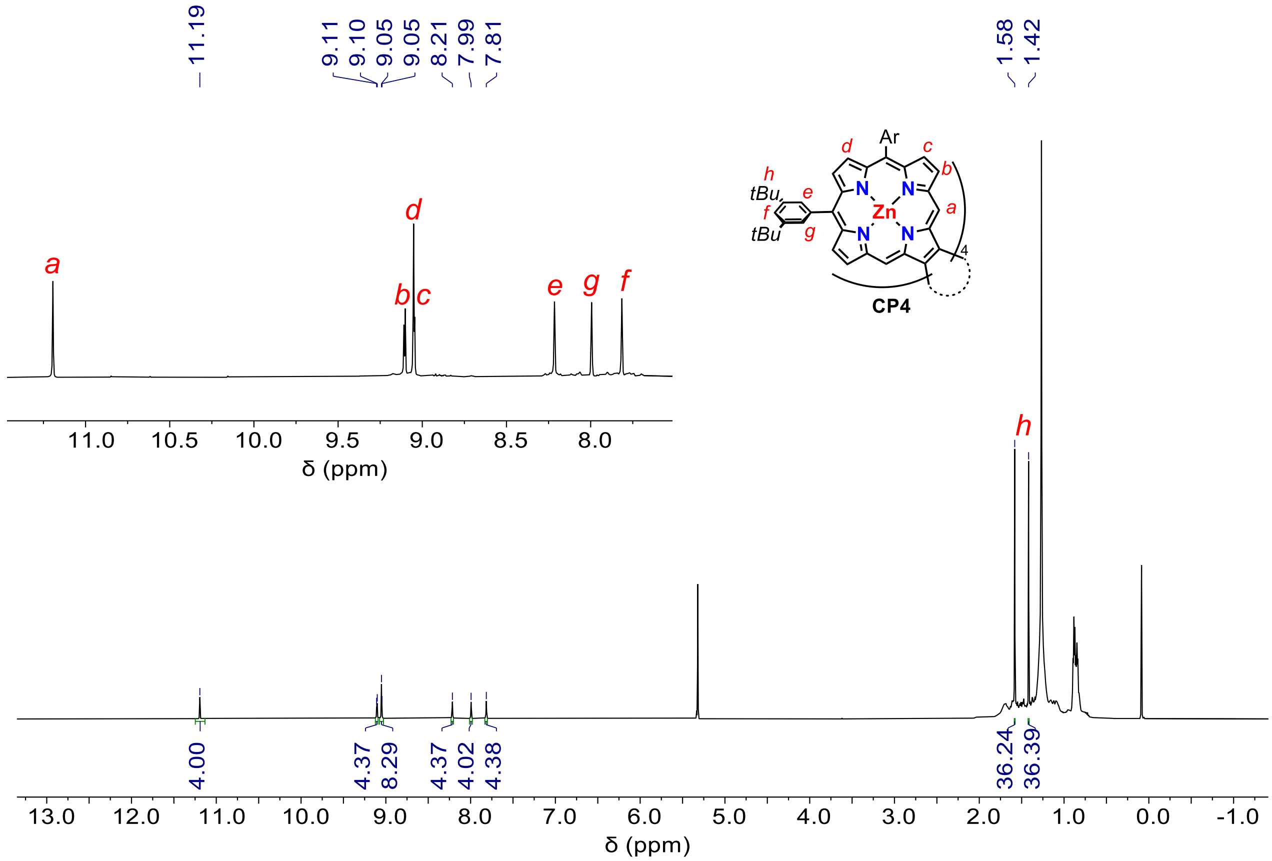

Figure S44. ^{1}H NMR spectrum of **CP4** in CD_2Cl_2 (500 MHz, 298 K).

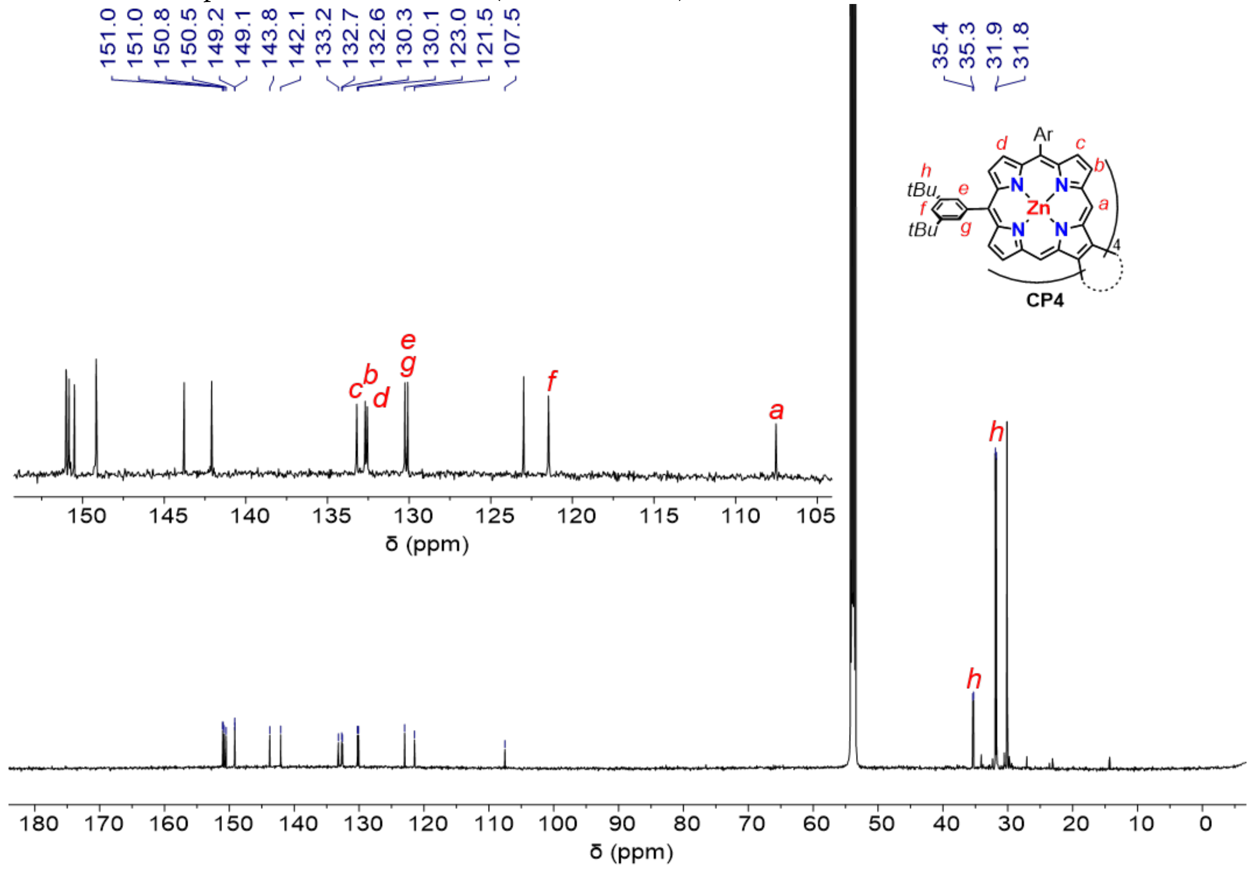

Figure S45. ^{13}C NMR spectrum of **CP4** in CD_2Cl_2 (126 MHz, 298 K).

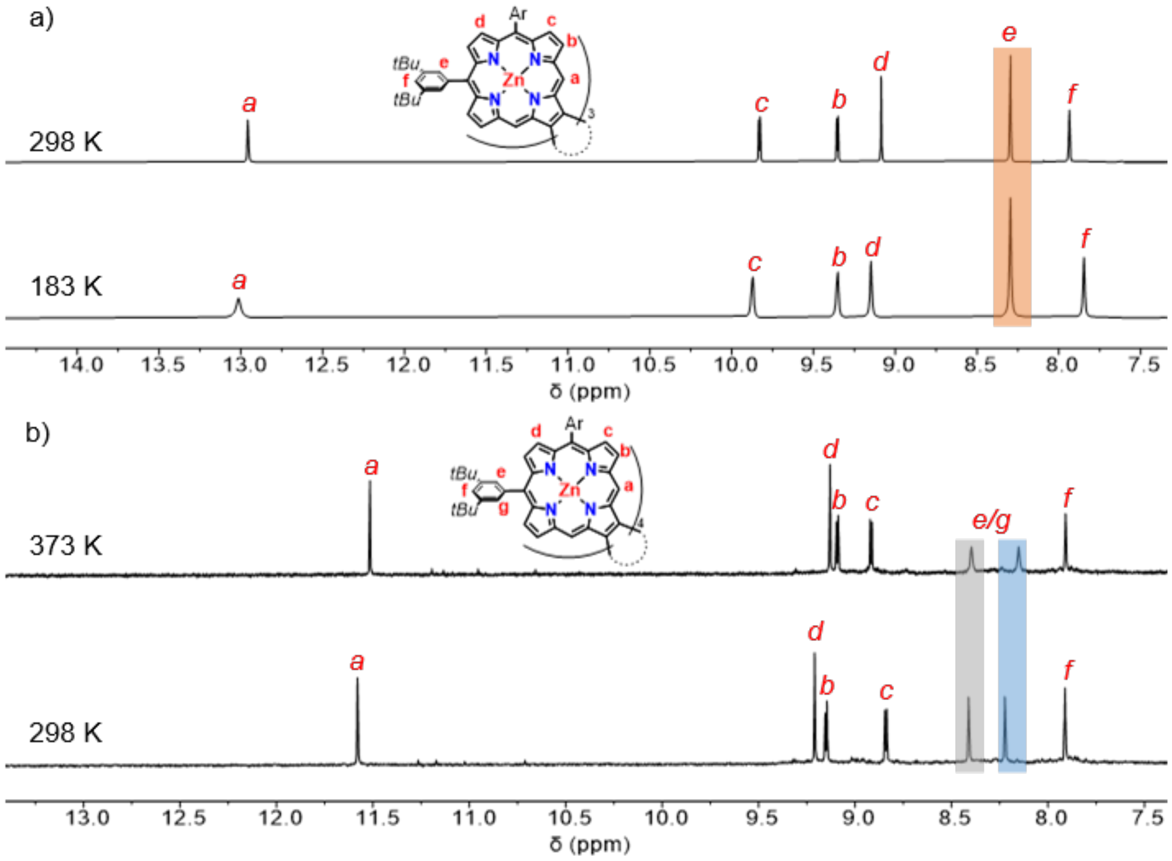

Figure S46. Comparison of aromatic region of ^{1}H NMR spectra of a) **CP3** measured at 298 K and 183 K in CD_2Cl_2 (500 MHz) and b) **CP4** measured at 373 K and 298 K in toluene-d_8 (500 MHz).

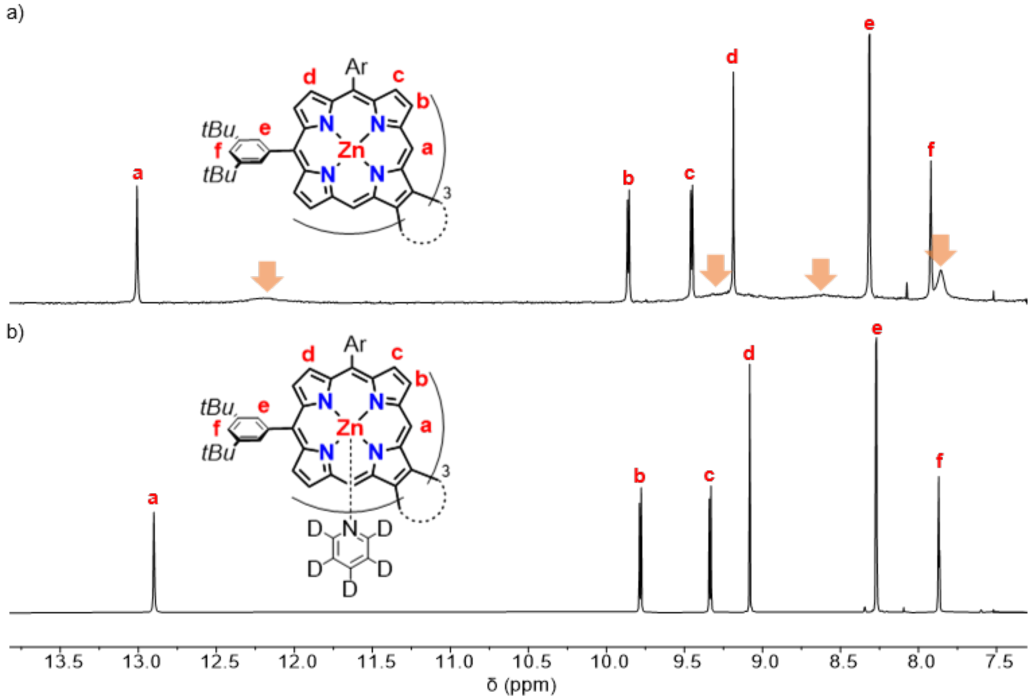

Figure S47. Comparison of aromatic region of ^{1}H NMR spectra of **CP3** measured in $CDCl_3$ (400 MHz) a) without and b) with 1% (*v*/*v*) pyridine-d_5.

MALDI-TOF MS Spectra

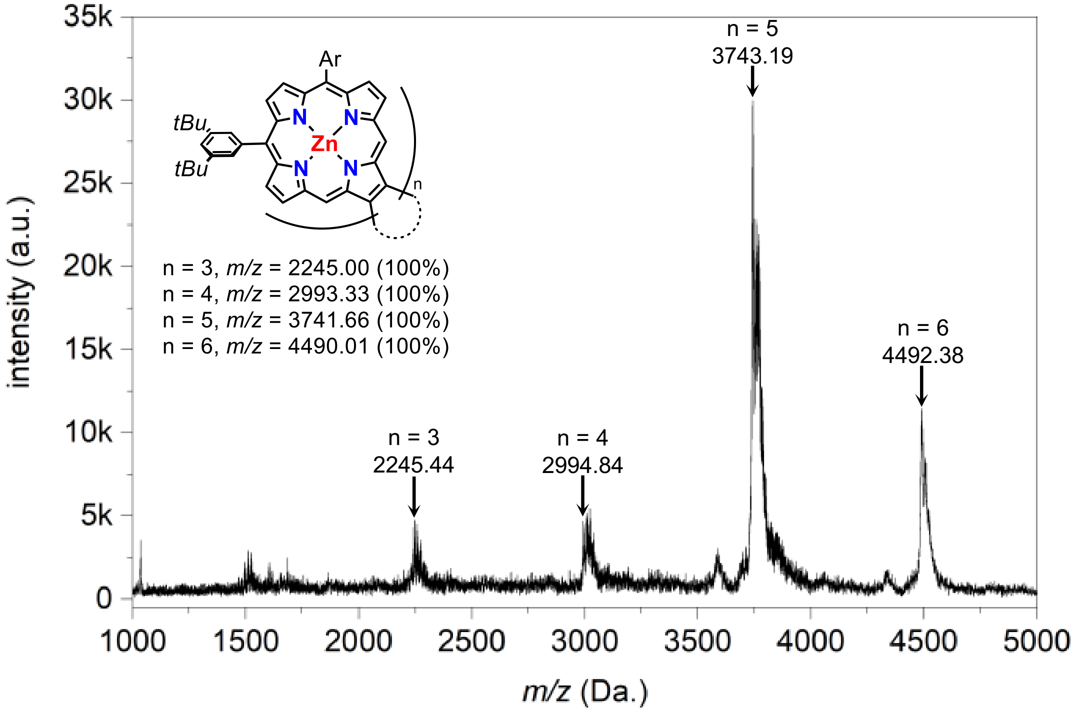

Figure S48. MALDI-TOF MS spectrum of the reaction mixture after Yamamoto coupling of 2,3-dibromo-10,15-bis(3,5-di-*tert*-butylphenyl)porphyrin (Zn) **1**, showing the formation of pentamer (*m/z* = 3743.19) and hexamer (*m/z* = 4492.38).

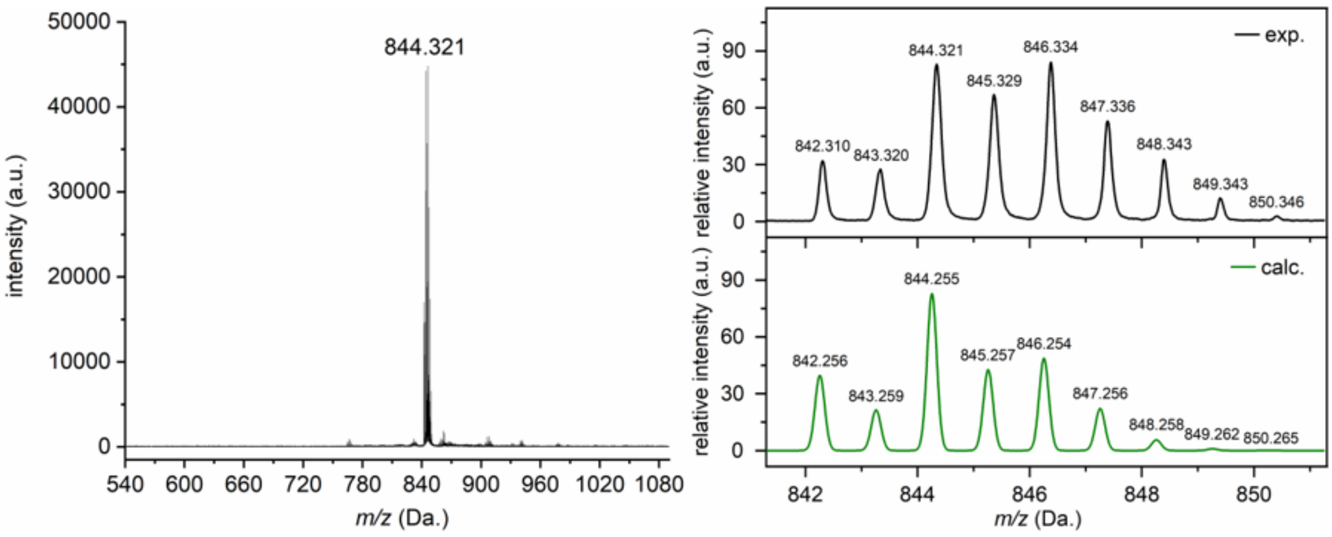

Figure S49. MALDI-TOF MS (DCTB in tetrahydrofuran as matrix) spectrum of 2,3-dibromo-10,15-bis(3,5-di-*tert*-butylphenyl)porphyrin (**6**), right: comparison of experimental isotropic distribution pattern with simulation.

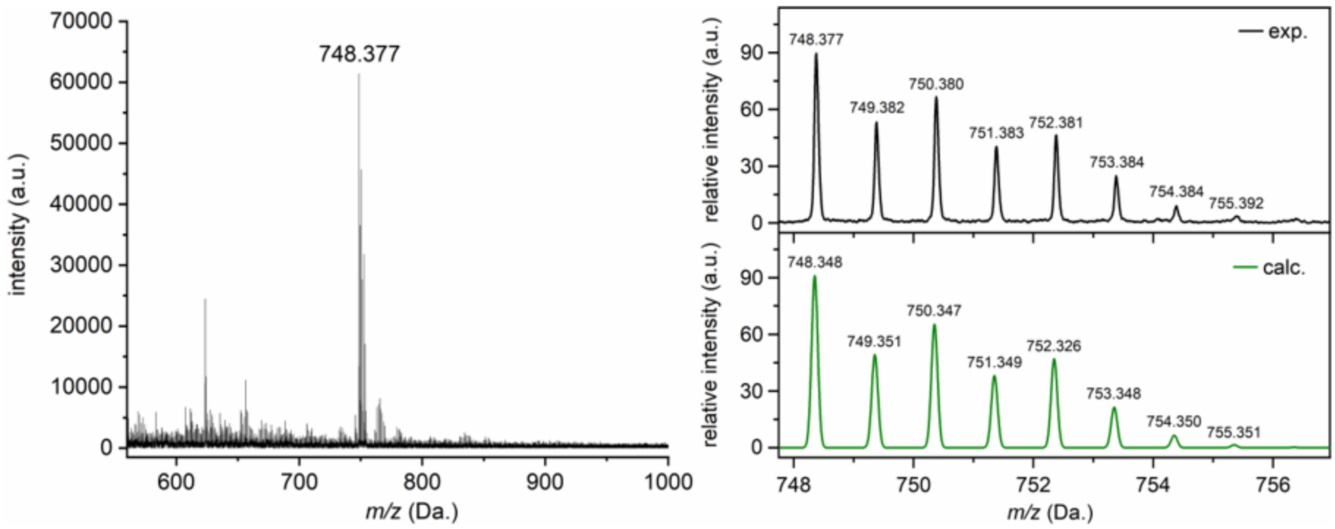

Figure S50. MALDI-TOF MS (DCTB in tetrahydrofuran as matrix) spectrum of 5,10-bis(3,5-di-*tert*-butylphenyl)porphyrin (**P1**), right: comparison of experimental isotropic distribution pattern with simulation.

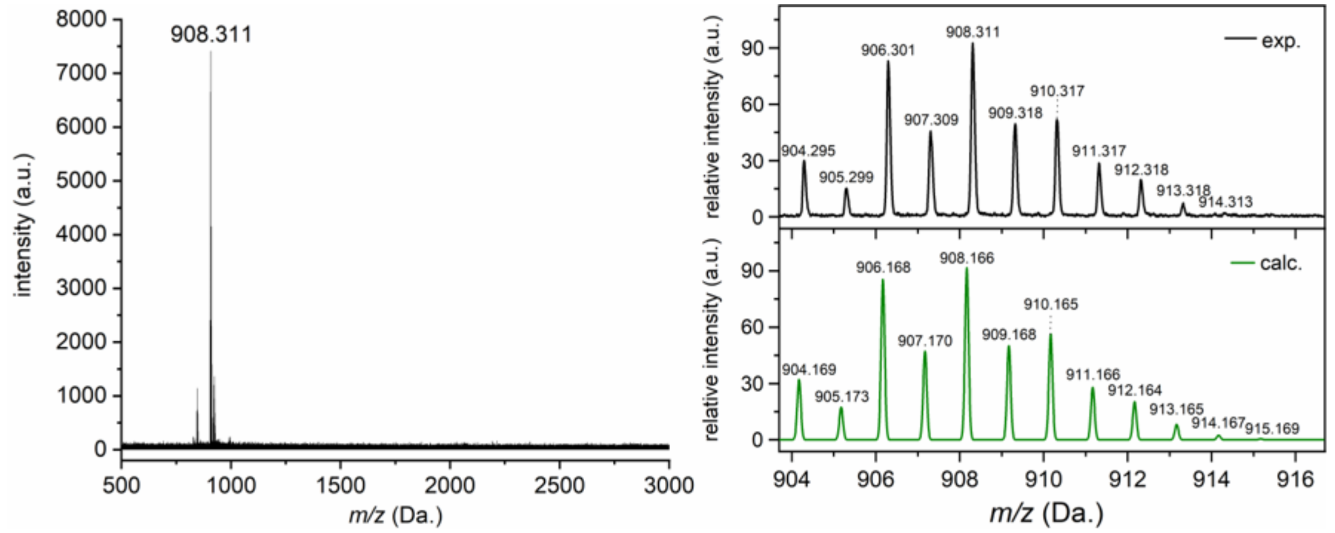

Figure S51. MALDI-TOF MS (DCTB in tetrahydrofuran as matrix) spectrum of compound **1**, right: comparison of experimental isotropic distribution pattern with simulation.

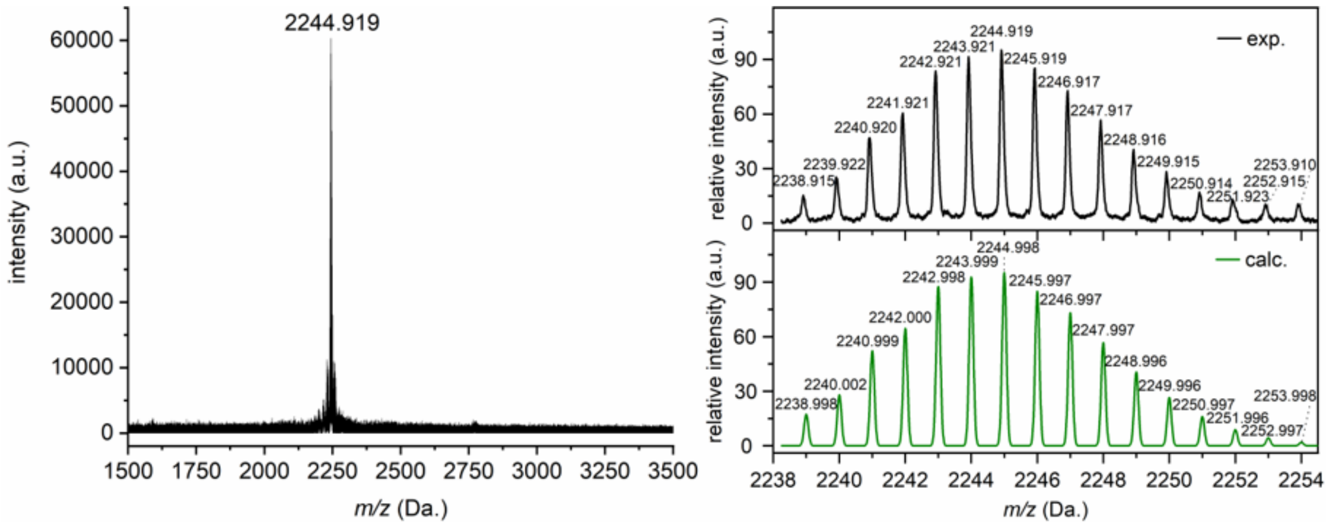

Figure S52. MALDI-TOF MS (DCTB in tetrahydrofuran as matrix) spectrum of **CP3**, right: comparison of experimental isotropic distribution pattern with simulation.

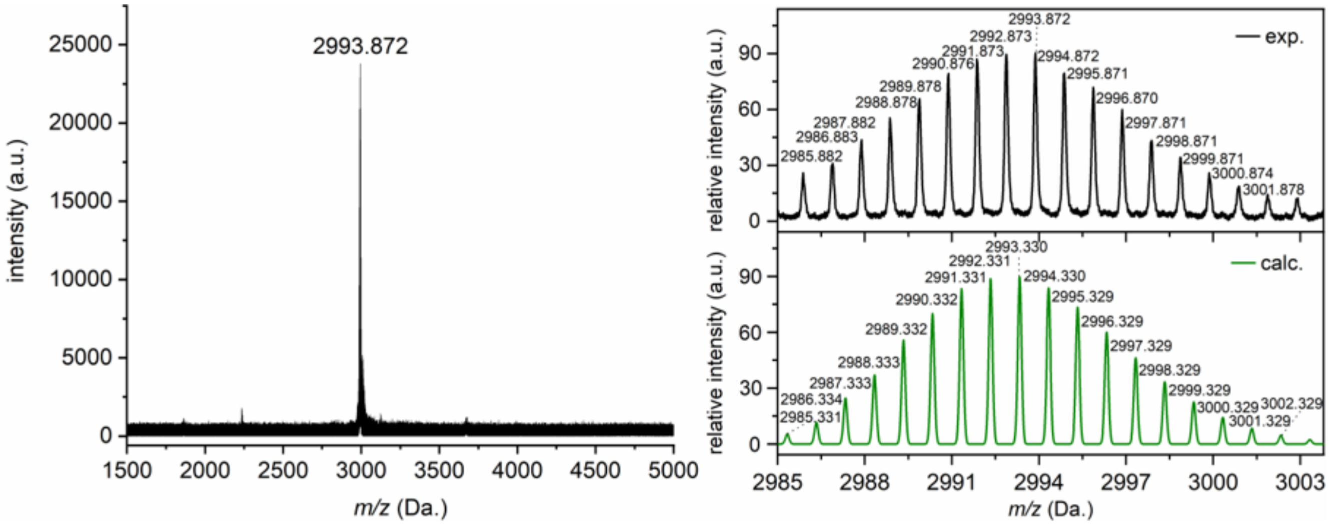

Figure S53. MALDI-TOF MS (DCTB in tetrahydrofuran as matrix) spectrum of **CP4**, right: comparison of experimental isotropic distribution pattern with simulation.

References

(1) Katritzky, A. R.; Law, K. W. A ^{13}C study of hydroxymethyl derivatives of five-membered ring heterocycles. *Magn. Reson. Chem.* **1988**, *26*, 129–133.

(2) Sudhakar, G.; Kadam, V. D.; Bayya, S.; Pranitha, G.; Jagadeesh, B. Total synthesis and stereochemical revision of acortatarins A and B. *Org. Lett.* **2011**, *13*, 5452–5455.

(3) Cadamuro, S.; Degani, I.; Fochi, R.; Gatti, A.; Piscopo, L. Convenient route for the synthesis of 3-substituted and 3,4-disubstituted pyrrole-2,5-dicarbaldehydes. *J. Chem. Soc., Perkin Trans. 1* **1996**, 2365–2369.

(4) Copley, G.; Hwang, D.; Kim, D.; Osuka, A. First-generation subporphyrinatoboron(III) sensitizers surpass the 10% power conversion efficiency threshold. *Angew. Chem. Int. Ed.* **2016**, *55*, 10287–10291.

(5) Nakamura, Y.; Hwang, I. W.; Aratani, N.; Ahn, T. K.; Ko, D. M.; Takagi, A.; Kawai, T.; Matsumoto, T.; Kim, D.; Osuka, A. Directly *meso-meso* linked porphyrin rings: synthesis, characterization, and efficient excitation energy hopping. *J. Am. Chem. Soc.* **2005**, *127*, 236–246.

(6) Taniguchi, M.; Lindsey, J. S.; Bocian, D. F.; Holten, D. Comprehensive review of photophysical parameters (ε, Φ_f, τ_s) of tetraphenylporphyrin (H_2TPP) and zinc tetraphenylporphyrin (ZnTPP) – Critical benchmark molecules in photochemistry and photosynthesis. *J. Photochem. Photobiol. C: Photochem. Rev.* **2021**, *46*, 100401.

(7) Wurth, C.; Grabolle, M.; Pauli, J.; Spieles, M.; Resch-Genger, U. Relative and absolute determination of fluorescence quantum yields of transparent samples. *Nat. Protoc.* **2013**, *8*, 1535-1550.

(8) Gaussian 16, Revision A.03. Frisch, M. J.; Trucks, G. W.; Schlegel, H. B.; Scuseria, G. E.; Robb, M. A.; Cheeseman, J. R.; Scalmani, G.; Barone, V.; Petersson, G. A.; Nakatsuji, H.; Li, X.; Caricato, M.; Marenich, A. V.; Bloino, J.; Janesko, B. G.; Gomperts, R.; Mennucci, B.; Hratchian, H. P.; Ortiz, J. V.; Izmaylov, A. F.; Sonnenberg, J. L.; Williams- Young, D.; Ding, F.; Lipparini, F.; Egidi, F.; Rega, N.; Zheng, G.; Liang, W.; Hada, M.; Ehara, M.; Toyota, K.; Fukuda, R.; Hasegawa, J.; Ishida, M.; Nakajima, T.; Honda, Y.; Kitao, O.; Nakai, H.; Vreven, T.; Throssell, K.; Montgomery Jr., J. A.; Peralta, J. E.; Ogliaro, F.; Bearpark, M. J.; Heyd, J. J.; Brothers, E. N.; Kudin, K. N.; Staroverov, V. N.; Keith, T. A.; Kobayashi, R.; Normand, J.; Raghavachari, K.; Rendell, A. P.; Burant, J. C.; Iyengar, S. S.; Tomasi, J.; Cossi, M.; Millam, J. M.; Klene, M.; Adamo, C.; Cammi, R.; Ochterski, J. W.; Martin, R. L.; Morokuma, K.; Farkas, O.; Foresman, J. B.; Fox, D. J. Gaussian 16, Revision A.03. Gaussian Inc.: Wallingford CT 2016.

(9) (a) Chen, Z.; Wannere, C. S.; Corminboeuf, C.; Puchta, R.; Schleyer, P. Nucleus-independent chemical shifts (NICS) as an aromaticity criterion. *Chem. Rev.* **2005**, *105*, 3842-3888. (b) Schleyer, P. V. R.; Maerker, C.; Dransfeld, A.; Jiao, H.; van Eikema Hommes, N. J. R. Nucleus-Independent Chemical Shifts: A Simple and Efficient Aromaticity Probe. *J. Am. Chem. Soc.* **1996**, *118*, 6317-6318.

(10) (a) Kruszewski, J.; Krygowski, T. M. Definition of aromaticity basing on the harmonic oscillator model. *Tetrahedron Lett.* **1972**, *13*, 3839–3842. (b) Krygowski, T. M.; Cyrański, M. K. Structural aspects of aromaticity. *Chem. Rev.* **2001**, *101*, 1385–1419. (c) Cyrański, M. K. Energetic aspects of cyclic pi-electron delocalization: Evaluation of the methods of estimating aromatic stabilization energies. *Chem. Rev.* **2005**, *105*, 3773–3811. (d) Krygowski, T. M.; Szatylowicz, H.; Stasyuk, O. A.; Dominikowska, J.; Palusiak, M. Aromaticity from the viewpoint of molecular geometry: application to planar systems. *Chem. Rev.* **2014**, *114*, 6383–6422. (e) Makino, M.; Nishina, N.; Aihara, J. Critical evaluation of HOMA and MBL as local aromaticity indices. *J. Phys. Org. Chem.* **2018**, *31*, e3783.

(11) Claus, K. H.; Krüger, C. Structure of cyclooctatetraene at 129 K. *Acta Crystallogr. C* **1988**, *44*, 1632–1634.

(12) Palatinus, L.; Chapuis, G. SUPERFLIP – a computer program for the solution of crystal structures by charge flipping in arbitrary dimensions. *J. Appl. Cryst.* **2007**, *40*, 786–790.

(13) Betteridge, P. W.; Carruthers, J. R.; Cooper, R. I.; Prout, K.; Watkin, D. J. CRYSTALS version 12: software for guided crystal structure analysis. *J. Appl. Cryst.* **2003**, *36*, 1487.

(14) Cooper, R. I.; Thompson, A. L. Watkin, D. J. CRYSTALS enhancements: dealing with hydrogen atoms in refinement. *J. Appl. Cryst.* **2010**, *43*, 1100–1107.

(15) Sheldrick, G. M. SHELXT – Integrated space-group and crystal-structure determination. *Acta Cryst.* **2015**, *A71*, 3–8.

(16) Dolomanov, O. V.; Bourhis, L. J.; Gildea, R.J.; Howard, J. A. K.; Puschmann, H. OLEX2: a complete structure solution, refinement and analysis program. *J. Appl. Cryst.* **2009**, *42*, 339–341.

(17) Sheldrick, G. M. Crystal structure refinement with SHELXL. *Acta Cryst.* **2015**, *C71*, 3–8.

(18) Allan, D. R. et al. A novel dual air-bearing fixed-χ diffractometer for small-molecule single-crystal X-ray diffraction on beamline I19 at Diamond Light Source. *Crystals* **2017**, *7*, 336.

(19) Winter, G. xia2: An expert system for macromolecular crystallography data reduction. *J. Appl. Cryst.* **2010**, *43*, 186–190

(20) Parois, P.; Cooper, R. I.; Thompson, A. L. Crystal structures of increasingly large molecules: meeting the challenges with CRYSTALS software. *Chem. Cent. J.* **2015**, *9*, 30.

(21) van der Sluis, P.; Spek, A. L. BYPASS: an effective method for the refinement of crystal structures containing disordered solvent regions. *Acta Cryst.* **1990**, *A46*, 194–201.

(22) Spek, A. L. Single-crystal structure validation with the program *PLATON. Appl. Cryst.* **2003**, 36, 7–13.

(23) Schröder, L.; Watkin, D. J.; Cousson, A.; Cooper, R. I.; Paulus, W. *CRYSTALS* enhancements: refinement of atoms continuously disordered along a line, on a ring or on the surface of a sphere. *J. Appl. Cryst.* **2004**, *37*, 545–550

Calculated Molecular Cartesian Coordinates

Cartesian coordinates of optimized geometry of **P1** (xyz format; number of atoms: 105)

C	4.252872	2.079803	-0.26014	C	3.922997	-3.50163	-3.20677
C	4.237923	3.436428	-0.37928	C	6.338144	-3.56256	-2.56692
C	2.85498	3.843222	-0.37516	C	4.688148	-5.12437	-1.46706
N	2.042157	2.741846	-0.24932	C	-4.89436	-3.69675	-2.02747
C	2.873551	1.647968	-0.1718	C	-3.92305	-3.50137	-3.20694
C	-0.6805	-1.46698	0.208054	C	-4.6882	-5.12422	-1.46734
C	0.680471	-1.46698	0.208059	C	-6.33819	-3.56232	-2.56707
C	1.105336	-0.10278	-0.00383	C	-6.2028	-1.84853	2.571154
N	-4E-06	0.711488	-0.12332	C	-7.23541	-2.9907	2.533603
C	-1.10535	-0.10277	-0.00382	C	-5.30255	-2.05561	3.812368
C	2.451674	0.312142	-0.04481	C	-6.96951	-0.51209	2.712805
C	-4.23789	3.436536	-0.3788	H	5.110591	1.424626	-0.24092
C	-4.25286	2.079898	-0.25982	H	5.081557	4.107839	-0.47199
C	-2.87354	1.648023	-0.17161	H	-1.34133	-2.30775	0.35521
N	-2.04213	2.741889	-0.2491	H	1.341285	-2.30776	0.35522
C	-2.85494	3.843295	-0.37479	H	-5.08152	4.107977	-0.47137
C	-2.45169	0.31218	-0.04474	H	-5.11059	1.42474	-0.2406
C	0.681468	6.979764	-0.6568	H	1.351166	7.826121	-0.73586
C	-0.68138	6.979792	-0.6566	H	-1.35107	7.826175	-0.73547
C	-1.09958	5.602518	-0.5291	H	3.192279	5.923305	-0.57547
N	0.000029	4.783977	-0.45315	H	-3.19221	5.9234	-0.5749
C	1.099644	5.602485	-0.5293	H	-3.04503	-1.5959	-1.84827
C	2.420845	5.163544	-0.49468	H	-6.23437	-3.47517	0.316311
C	-2.42078	5.163613	-0.49428	H	-4.17627	-0.1005	1.983841
C	-3.50907	-0.74543	0.059246	H	3.044994	-1.59606	-1.84823
C	3.509053	-0.74547	0.059228	H	6.234346	-3.4752	0.316453
Zn	0.000014	2.746153	-0.26892	H	4.176271	-0.10041	1.983773
C	-3.68725	-1.67427	-0.97956	H	7.829902	-2.97919	3.452866
C	-4.6673	-2.66617	-0.90413	H	6.755803	-3.9724	2.464127
C	-5.47245	-2.70827	0.249077	H	7.928549	-2.88737	1.692374
C	-5.32647	-1.80342	1.304157	H	7.59065	-0.52208	3.615516
C	-4.32995	-0.82036	1.186225	H	6.290115	0.342081	2.786264
C	3.687222	-1.67438	-0.97952	H	7.625588	-0.34453	1.852086
C	4.667273	-2.66628	-0.90403	H	4.751388	-2.99894	3.743011
C	5.472422	-2.7083	0.249173	H	5.910564	-2.08335	4.723702
C	5.326463	-1.80338	1.304194	H	4.571407	-1.24989	3.925109
C	4.329942	-0.82033	1.186205	H	4.120704	-4.25586	-3.97527
C	6.202801	-1.84842	2.571188	H	2.878471	-3.61135	-2.89779
C	7.235418	-2.99058	2.533697	H	4.039691	-2.51791	-3.67272
C	6.969506	-0.51196	2.712756	H	6.516638	-4.29258	-3.3645
C	5.302552	-2.05543	3.812419	H	7.083813	-3.73531	-1.78539
C	4.894314	-3.69694	-2.0273	H	6.510388	-2.56236	-2.9776

H	5.380848	-5.34691	-0.65023
H	3.669879	-5.25137	-1.08507
H	4.850933	-5.86976	-2.25369
H	-4.12076	-4.25555	-3.97548
H	-4.03974	-2.51762	-3.67282
H	-2.87852	-3.61111	-2.89797
H	-4.85101	-5.86955	-2.25401
H	-5.38089	-5.34682	-0.65051
H	-3.66993	-5.25127	-1.08537
H	-6.51043	-2.56209	-2.97767
H	-6.5167	-4.29228	-3.36471
H	-7.08386	-3.73513	-1.78555
H	-7.8299	-2.97936	3.452769
H	-7.92854	-2.88746	1.692281
H	-6.75579	-3.97252	2.463987
H	-5.91055	-2.08359	4.723653
H	-4.5714	-1.25009	3.925096
H	-4.75138	-2.99912	3.742905
H	-7.62559	-0.34461	1.852143
H	-7.59066	-0.52227	3.615563
H	-6.29012	0.341948	2.786371

Cartesian coordinates of optimized geometry of **CP3** (xyz format; number of atoms: 309)

PMP conformer, E(RB3LYP) = -11572.7628298 a.u.

C	6.591913	3.350544	1.248731
C	5.9003	2.176904	1.230783
C	4.529479	2.5029	0.92732
N	4.385857	3.861268	0.808882
C	5.640863	4.40808	0.986342
C	4.1487	8.87104	0.4782
C	5.326567	8.206386	0.639715
C	5.019433	6.795544	0.637069
N	3.667151	6.616362	0.454242
C	3.110719	7.87253	0.376493
C	5.958007	5.772989	0.886322
C	-1.34774	6.268364	0.287546
C	-0.70317	7.468395	0.281565
C	0.715213	7.188649	0.328002
N	0.911539	5.822423	0.354451
C	-0.33148	5.246179	0.304826
C	1.731109	8.158507	0.31289
C	1.219912	0.732544	0.036212
C	-0.01203	1.41606	-0.07652
C	0.30148	2.837149	0.069348
N	1.654934	3.00177	0.220438
C	2.229489	1.757714	0.28186
C	3.541417	1.543315	0.694879
C	-0.60954	3.881942	0.19078
Zn	2.658471	4.827361	0.441525
C	-9.74128	-0.73646	0.385513
C	-9.76241	0.399009	-0.36747
C	-8.38951	0.782858	-0.59191
N	-7.55037	-0.131	0.004166
C	-8.35502	-1.07315	0.603583
C	-4.72964	-3.73254	2.100005
C	-6.08959	-3.73607	2.179671
C	-6.55713	-2.54637	1.503023
N	-5.4744	-1.83689	1.022225
C	-4.35486	-2.5477	1.369876
C	-7.90586	-2.20028	1.323047
C	-1.22095	0.673153	-0.14555
C	-1.19626	-0.71162	0.135175
C	-2.58902	-1.08731	0.373968
N	-3.41471	-0.0582	-0.00248
C	-2.62675	0.99939	-0.38124
C	-3.03437	-2.21995	1.047328
C	-6.22404	3.52031	-2.17986
C	-4.86455	3.565085	-2.10432
C	-4.44591	2.395439	-1.37343
N	-5.53857	1.646087	-1.02135
C	-6.6471	2.31626	-1.4996
C	-7.98233	1.924039	-1.31408
C	-3.1137	2.115025	-1.05416
Zn	-5.50026	-0.09514	0.000602
C	-0.44172	-7.48394	-0.29764
C	-1.12788	-6.3072	-0.30339
C	-0.14789	-5.25015	-0.31939
N	1.114508	-5.78261	-0.36852
C	0.965978	-7.15481	-0.34278
C	5.610226	-8.01144	-0.6485
C	4.456002	-8.71678	-0.48892
C	3.383708	-7.75511	-0.38833
N	3.896181	-6.48025	-0.46501
C	5.254146	-6.61212	-0.64577

C	2.014841	-8.08892	-0.32639
C	5.974229	-1.96546	-1.23841
C	6.706535	-3.11417	-1.25465
C	5.792566	-4.20434	-0.9939
N	4.518882	-3.7017	-0.81886
C	4.615087	-2.33913	-0.93734
C	6.156991	-5.55736	-0.89312
C	0.037828	-1.4116	0.064499
C	1.244949	-0.68531	-0.04869
C	2.289836	-1.67454	-0.2943
N	1.759104	-2.9379	-0.23312
C	0.400712	-2.82075	-0.08201
C	-0.47337	-3.89653	-0.20446
C	3.593847	-1.41457	-0.70637
Zn	2.825787	-4.72743	-0.45396
H	7.648901	3.495327	1.413441
H	6.280272	1.174153	1.376648
H	3.988894	9.938566	0.459485
H	6.310596	8.62842	0.777626
H	-2.41299	6.081111	0.251996
H	-1.13877	8.455215	0.241012
H	3.841487	0.519085	0.866257
H	-1.65815	3.623261	0.20325
H	-10.5845	-1.29972	0.755738
H	-10.626	0.933224	-0.73369
H	-4.03539	-4.46722	2.486459
H	-6.72567	-4.47568	2.641992
H	-2.28118	-2.90558	1.411922
H	-6.88755	4.235898	-2.64156
H	-4.19782	4.323103	-2.49417
H	-2.38629	2.826543	-1.42151
H	-0.84237	-8.48554	-0.25805
H	-2.19904	-6.1572	-0.26843
H	6.608524	-8.39892	-0.78523
H	4.333329	-9.78921	-0.47073
H	6.319178	-0.95006	-1.38379
H	7.768284	-3.22185	-1.41714
H	-1.53037	-3.67454	-0.21701
H	3.858203	-0.38052	-0.87762
C	7.602339	-5.90901	-1.07962
C	8.206907	-5.78491	-2.33727
C	8.360859	-6.36837	0.00493
C	9.555269	-6.11007	-2.52976
H	7.599751	-5.43635	-3.16383
C	9.712284	-6.70294	-0.14416
H	7.874324	-6.45258	0.969081
C	10.27992	-6.56398	-1.41866
H	11.3265	-6.81975	-1.55119
C	1.656812	-9.54335	-0.26151
C	1.933685	-10.2854	0.893977
C	1.046813	-10.1742	-1.35349
C	1.608807	-11.6447	0.978954
H	2.402497	-9.7766	1.727482
C	0.707124	-11.532	-1.3105
H	0.850283	-9.58389	-2.24016
C	0.998238	-12.2384	-0.13489
H	0.740325	-13.2916	-0.08513
C	-8.9413	-3.10419	1.923124
C	-9.11204	-3.16149	3.307327
C	-9.74728	-3.90725	1.099483
C	-10.0729	-4.00241	3.89329
H	-8.48487	-2.53168	3.930346
C	-10.7137	-4.75779	1.640629
H	-9.59448	-3.8539	0.028451
C	-10.8557	-4.78519	3.040311
H	-11.603	-5.44188	3.468533
C	-9.05064	2.790089	-1.91273
C	-9.87407	3.570922	-1.09144
C	-9.23112	2.82656	-3.30124
C	-10.8753	4.386196	-1.63315
H	-9.71229	3.532661	-0.02106
C	-10.2216	3.62713	-3.88358
H	-8.58577	2.211555	-3.9166
C	-11.0253	4.392812	-3.02725
H	-11.7979	5.018724	-3.46276
C	1.323613	9.599786	0.2476
C	1.579826	10.35348	-0.90967
C	0.68989	10.21073	1.331148
C	1.210106	11.69748	-0.99483
H	2.068761	9.860256	-1.74097
C	0.302647	11.56047	1.288384
H	0.506638	9.618153	2.221824
C	0.573182	12.27578	0.11861
H	0.283431	13.31787	0.061326
C	7.389821	6.174689	1.076397
C	7.994074	6.073806	2.33626
C	8.135742	6.657539	-0.00666
C	9.329876	6.44523	2.532492
H	7.396489	5.706244	3.161557
C	9.474566	7.038085	0.14611

H	7.649781	6.723263	-0.97254
C	10.04238	6.921126	1.422737
H	11.07927	7.212481	1.558006
C	10.5778	-7.20519	1.0284
C	10.25061	-5.99119	-3.90044
C	11.12158	-8.61601	0.700422
H	11.73493	-8.61707	-0.20535
H	11.74314	-8.98602	1.523618
H	10.30181	-9.32586	0.548883
C	11.76463	-6.23674	1.245436
H	12.39858	-6.16242	0.357027
H	11.40859	-5.22986	1.486497
H	12.39245	-6.58261	2.07438
C	9.785128	-7.29287	2.346288
H	9.396698	-6.31688	2.654475
H	8.942278	-7.98739	2.270309
H	10.43951	-7.65462	3.145936
C	10.77538	-7.38034	-4.33453
H	11.27639	-7.31208	-5.30676
H	11.49477	-7.78705	-3.61774
H	9.953369	-8.09771	-4.42592
C	9.299837	-5.475	-4.99702
H	8.448915	-6.14679	-5.14875
H	8.910856	-4.47847	-4.76436
H	9.838126	-5.4051	-5.94772
C	11.43859	-5.00599	-3.79231
H	12.17846	-5.33702	-3.05767
H	11.94715	-4.91437	-4.75866
H	11.09517	-4.00994	-3.49428
C	1.892657	-12.4893	2.236922
C	2.829549	-13.6642	1.868922
H	3.036859	-14.2778	2.752888
H	2.388904	-14.3155	1.108465
H	3.784629	-13.2961	1.480057
C	2.569157	-11.67	3.352249
H	3.534206	-11.2641	3.032248
H	1.941546	-10.8369	3.684653
H	2.752526	-12.3113	4.220345
C	0.562062	-13.0502	2.792315
H	0.049237	-13.6825	2.061617
H	0.747223	-13.657	3.685898
H	-0.11939	-12.2389	3.068121
C	0.040362	-12.259	-2.4951
C	0.946186	-13.423	-2.96225
H	1.118099	-14.152	-2.16491
H	0.483625	-13.9523	-3.80296
H	1.922199	-13.0514	-3.29082
C	-1.32901	-12.8236	-2.04826
H	-1.81451	-13.3485	-2.87874
H	-1.22832	-13.5321	-1.22086
H	-1.99549	-12.0195	-1.71955
C	-0.1964	-11.3256	-3.69741
H	-0.85702	-10.4906	-3.44263
H	0.740937	-10.9135	-4.08471
H	-0.67105	-11.8852	-4.50982
C	-10.2269	-4.02737	5.426566
C	-11.6116	-5.65659	0.767926
C	-13.096	-5.29335	1.010008
H	-13.7481	-5.92795	0.399347
H	-13.3849	-5.42973	2.05619
H	-13.2919	-4.24968	0.742982
C	-11.3794	-7.13916	1.144726
H	-11.6134	-7.33494	2.195231
H	-12.0147	-7.79154	0.535007
H	-10.3366	-7.4267	0.975679
C	-11.3191	-5.49386	-0.73565
H	-11.4908	-4.46766	-1.07618
H	-10.2884	-5.76771	-0.98288
H	-11.9811	-6.1478	-1.31233
C	-11.3249	-5.00154	5.893167
H	-11.1097	-6.03291	5.595013
H	-11.3933	-4.9825	6.985573
H	-12.3084	-4.72829	5.4971
C	-8.88995	-4.46662	6.069712
H	-8.60896	-5.47221	5.739552
H	-8.07099	-3.78919	5.811506
H	-8.97845	-4.48083	7.161866
C	-10.5956	-2.61136	5.929417
H	-9.83113	-1.87401	5.668465
H	-11.5437	-2.27684	5.495863
H	-10.7013	-2.60987	7.020142
C	-11.7964	5.261252	-0.7601
C	-10.4529	3.68577	-5.40652
C	-11.482	5.126011	0.741822
H	-11.6135	4.098426	1.09578
H	-10.4591	5.439025	0.97431
H	-12.1606	5.762673	1.318581
C	-13.2686	4.84061	-0.98233
H	-13.9378	5.4572	-0.37164
H	-13.5728	4.954547	-2.02691

H	-13.4229	3.793379	-0.70234
C	-11.6233	6.747033	-1.15504
H	-11.8757	6.922477	-2.20487
H	-12.2757	7.382179	-0.54526
H	-10.5901	7.074893	-1.0009
C	-11.893	3.219859	-5.72711
H	-12.6435	3.849806	-5.24058
H	-12.0751	3.258852	-6.80704
H	-12.0543	2.189879	-5.39274
C	-10.2628	5.138969	-5.90209
H	-10.4287	5.197561	-6.98369
H	-10.9615	5.829634	-5.42108
H	-9.24766	5.493376	-5.69519
C	-9.47447	2.784211	-6.18288
H	-8.43237	3.07796	-6.02088
H	-9.57994	1.731178	-5.90288
H	-9.67568	2.861406	-7.25619
C	-0.38907	12.19132	2.51271
C	1.469226	12.55219	-2.25099
C	2.367427	13.75604	-1.87978
H	2.556364	14.37713	-2.76264
H	1.905014	14.39187	-1.1192
H	3.333198	13.41797	-1.49003
C	2.173744	11.75644	-3.36598
H	3.150968	11.38172	-3.04481
H	1.573983	10.90372	-3.7
H	2.337595	12.40413	-4.23326
C	0.122594	13.07107	-2.80884
H	-0.41208	13.68579	-2.07886
H	0.290581	13.68456	-3.70122
H	-0.53171	12.23862	-3.08749
C	-0.74191	13.6742	2.29159
H	-1.43243	13.80915	1.45261
H	-1.22972	14.07351	3.186577
H	0.149023	14.28223	2.10346

C	-1.69939	11.42561	2.813781
H	-2.38996	11.48372	1.965871
H	-1.51503	10.36798	3.022944
H	-2.20006	11.85545	3.688671
C	0.550574	12.09867	3.738438
H	0.806228	11.06287	3.979055
H	1.485126	12.6394	3.556614
H	0.070463	12.53694	4.620659
C	10.02426	6.352731	3.905684
C	10.504	7.758941	4.3369
H	11.00367	7.709326	5.310945
H	11.21276	8.1857	3.621202
H	9.659701	8.45058	4.423359
C	9.086659	5.810632	5.001123
H	8.21421	6.455497	5.147336
H	8.73049	4.801288	4.771164
H	9.623632	5.76156	5.953868
C	11.24279	5.404834	3.804315
H	11.97395	5.756448	3.070523
H	11.7512	5.332477	4.772381
H	10.93156	4.397516	3.508783
C	10.32655	7.566657	-1.0248
C	10.82197	8.995461	-0.69813
H	11.43209	9.018781	0.209542
H	11.43348	9.384309	-1.52016
H	9.978529	9.677905	-0.55068
C	11.54566	6.637857	-1.23609
H	12.16419	7.002683	-2.06389
H	12.17896	6.586622	-0.34559
H	11.22418	5.619131	-1.47595
C	9.535514	7.625199	-2.34528
H	9.181018	6.636135	-2.65269
H	8.669603	8.291171	-2.27323
H	10.17987	8.007168	-3.14366

Cartesian coordinates of optimized geometry of **CP3** (xyz format; number of atoms: 309)

MPM conformer, E(RB3LYP) = -11572.7628298 a.u.

C	-6.59194	3.350492	1.248737
C	-5.90031	2.176858	1.230789
C	-4.5295	2.502865	0.927323
N	-4.38589	3.861234	0.808885
C	-5.6409	4.408035	0.986346
C	-4.14877	8.871008	0.478199

C	-5.32663	8.206344	0.639717
C	-5.01949	6.795504	0.637071
N	-3.6672	6.616333	0.454242
C	-3.11078	7.872506	0.376491
C	-5.95805	5.772942	0.886327
C	1.347692	6.268378	0.287538

C	0.703108	7.468403	0.281558
C	-0.71527	7.188646	0.327996
N	-0.91159	5.822418	0.354446
C	0.331435	5.246184	0.30482
C	-1.73118	8.158495	0.312885
C	-1.21992	0.732535	0.036212
C	0.012022	1.41606	-0.07653
C	-0.3015	2.837148	0.069346
N	-1.65496	3.001758	0.220437
C	-2.2295	1.757698	0.28186
C	-3.54143	1.543287	0.694882
C	0.609513	3.881949	0.190776
Zn	-2.65851	4.827341	0.441524
C	9.741284	-0.73639	0.385516
C	9.762412	0.399081	-0.36747
C	8.389504	0.78292	-0.59191
N	7.55037	-0.13094	0.004167
C	8.35503	-1.07309	0.603585
C	4.729671	-3.7325	2.100008
C	6.08962	-3.73602	2.179674
C	6.557144	-2.54632	1.503026
N	5.474417	-1.83684	1.022226
C	4.354876	-2.54766	1.369878
C	7.905876	-2.20022	1.32305
C	1.22095	0.673163	-0.14555
C	1.196269	-0.71161	0.135175
C	2.589032	-1.08729	0.373968
N	3.414715	-0.05817	-0.00248
C	2.626743	0.999411	-0.38124
C	3.034384	-2.21992	1.047329
C	6.224012	3.520355	-2.17986
C	4.864523	3.565121	-2.10433
C	4.445894	2.395472	-1.37343
N	5.538556	1.646128	-1.02135
C	6.647086	2.316309	-1.4996
C	7.982318	1.924097	-1.31408
C	3.113685	2.115048	-1.05416
Zn	5.500263	-0.0951	0.000603
C	0.44178	-7.48394	-0.29764
C	1.127933	-6.30719	-0.30339
C	0.147934	-5.25015	-0.31939
N	-1.11446	-5.78261	-0.36852
C	-0.96592	-7.15482	-0.34278
C	-5.61016	-8.01148	-0.6485
C	-4.45594	-8.71682	-0.48892
C	-3.38365	-7.75514	-0.38833
N	-3.89613	-6.48028	-0.46501
C	-5.2541	-6.61216	-0.64578
C	-2.01478	-8.08893	-0.32639
C	-5.97421	-1.96551	-1.23841
C	-6.70651	-3.11422	-1.25466
C	-5.79253	-4.20438	-0.9939
N	-4.51885	-3.70173	-0.81886
C	-4.61507	-2.33917	-0.93734
C	-6.15695	-5.5574	-0.89312
C	-0.03782	-1.4116	0.064499
C	-1.24494	-0.68531	-0.04869
C	-2.28982	-1.67455	-0.2943
N	-1.75908	-2.93791	-0.23313
C	-0.40069	-2.82076	-0.08201
C	0.473402	-3.89653	-0.20446
C	-3.59383	-1.41459	-0.70637
Zn	-2.82575	-4.72745	-0.45397
H	-7.64893	3.495266	1.413449
H	-6.28028	1.174104	1.376655
H	-3.98898	9.938535	0.459483
H	-6.31067	8.628369	0.777629
H	2.412946	6.081133	0.251987
H	1.138697	8.455227	0.241004
H	-3.84149	0.519056	0.866261
H	1.658118	3.623276	0.203244
H	10.58452	-1.29964	0.755743
H	10.62601	0.933302	-0.73368
H	4.035424	-4.46719	2.486461
H	6.7257	-4.47563	2.641996
H	2.281201	-2.90556	1.411923
H	6.887519	4.235947	-2.64156
H	4.197787	4.323134	-2.49417
H	2.38627	2.826562	-1.42151
H	0.842439	-8.48553	-0.25805
H	2.199088	-6.15719	-0.26843
H	-6.60846	-8.39897	-0.78523
H	-4.33325	-9.78924	-0.47074
H	-6.31917	-0.95011	-1.38379
H	-7.76826	-3.22191	-1.41715
H	1.530395	-3.67453	-0.21701
H	-3.8582	-0.38055	-0.87762
C	-7.60229	-5.90907	-1.07962
C	-8.20686	-5.78497	-2.33728
C	-8.36081	-6.36843	0.004928

C	-9.55522	-6.11014	-2.52976
H	-7.59971	-5.4364	-3.16383
C	-9.71223	-6.70301	-0.14416
H	-7.87428	-6.45264	0.969078
C	-10.2799	-6.56406	-1.41866
H	-11.3264	-6.81983	-1.5512
C	-1.65674	-9.54336	-0.26152
C	-1.93361	-10.2854	0.893974
C	-1.04674	-10.1742	-1.35349
C	-1.60872	-11.6448	0.978951
H	-2.40242	-9.77662	1.72748
C	-0.70704	-11.532	-1.3105
H	-0.85021	-9.58389	-2.24016
C	-0.99815	-12.2384	-0.13489
H	-0.74022	-13.2916	-0.08513
C	8.941323	-3.10412	1.923128
C	9.11206	-3.16142	3.307332
C	9.747312	-3.90718	1.099489
C	10.07298	-4.00234	3.893296
H	8.484889	-2.53161	3.93035
C	10.71378	-4.75771	1.640637
H	9.594516	-3.85382	0.028457
C	10.85572	-4.7851	3.040319
H	11.60307	-5.44179	3.468542
C	9.050625	2.790154	-1.91273
C	9.874046	3.570994	-1.09144
C	9.231105	2.826625	-3.30124
C	10.87531	4.386275	-1.63315
H	9.712267	3.532733	-0.02106
C	10.22161	3.627202	-3.88358
H	8.585751	2.211616	-3.9166
C	11.0253	4.39289	-3.02725
H	11.79786	5.018808	-3.46277
C	-1.32369	9.599777	0.247594
C	-1.57991	10.35347	-0.90967
C	-0.68997	10.21073	1.33114
C	-1.2102	11.69747	-0.99484
H	-2.06885	9.860241	-1.74098
C	-0.30274	11.56047	1.288375
H	-0.50671	9.618152	2.221816
C	-0.57328	12.27578	0.1186
H	-0.28354	13.31787	0.061316
C	-7.38987	6.17463	1.076404
C	-7.99412	6.073742	2.336268
C	-8.1358	6.657473	-0.00665
C	-9.32992	6.445155	2.532502
H	-7.39653	5.706186	3.161564
C	-9.47462	7.038007	0.14612
H	-7.64984	6.7232	-0.97254
C	-10.0424	6.921045	1.422748
H	-11.0793	7.212391	1.558019
C	-10.5777	-7.20526	1.028396
C	-10.2506	-5.99126	-3.90044
C	-11.1215	-8.61609	0.700417
H	-11.7349	-8.61715	-0.20536
H	-11.7431	-8.98611	1.523613
H	-10.3017	-9.32594	0.548878
C	-11.7646	-6.23682	1.245432
H	-12.3985	-6.16251	0.357022
H	-11.4086	-5.22994	1.486493
H	-12.3924	-6.5827	2.074375
C	-9.78507	-7.29295	2.346284
H	-9.39665	-6.31695	2.654472
H	-8.94222	-7.98746	2.270305
H	-10.4395	-7.6547	3.145932
C	-10.7753	-7.38042	-4.33453
H	-11.2763	-7.31216	-5.30676
H	-11.4947	-7.78713	-3.61774
H	-9.95331	-8.09778	-4.42592
C	-9.29979	-5.47507	-4.99702
H	-8.44887	-6.14685	-5.14876
H	-8.91082	-4.47854	-4.76436
H	-9.83808	-5.40517	-5.94772
C	-11.4386	-5.00607	-3.79231
H	-12.1784	-5.33711	-3.05768
H	-11.9471	-4.91446	-4.75866
H	-11.0951	-4.01001	-3.49428
C	-1.89256	-12.4893	2.236919
C	-2.82945	-13.6642	1.86892
H	-3.03675	-14.2778	2.752886
H	-2.3888	-14.3155	1.108462
H	-3.78453	-13.2961	1.480055
C	-2.56907	-11.67	3.352247
H	-3.53412	-11.2641	3.032246
H	-1.94146	-10.837	3.684651
H	-2.75243	-12.3114	4.220343
C	-0.56196	-13.0502	2.792311
H	-0.04913	-13.6825	2.061612
H	-0.74712	-13.657	3.685894
H	0.119481	-12.2389	3.068117

C	-0.04027	-12.259	-2.4951
C	-0.94609	-13.423	-2.96225
H	-1.11799	-14.152	-2.16491
H	-0.48352	-13.9523	-3.80297
H	-1.9221	-13.0514	-3.29082
C	1.329103	-12.8236	-2.04826
H	1.814606	-13.3485	-2.87874
H	1.228418	-13.5321	-1.22086
H	1.995579	-12.0195	-1.71956
C	0.196489	-11.3256	-3.69741
H	0.857101	-10.4906	-3.44264
H	-0.74086	-10.9135	-4.08471
H	0.671136	-11.8852	-4.50982
C	10.2269	-4.02728	5.426573
C	11.61165	-5.6565	0.767935
C	13.096	-5.29325	1.010017
H	13.74811	-5.92784	0.399358
H	13.38492	-5.42962	2.0562
H	13.29191	-4.24958	0.742991
C	11.37948	-7.13907	1.144736
H	11.61348	-7.33485	2.195241
H	12.01476	-7.79145	0.535018
H	10.33666	-7.42662	0.975689
C	11.31913	-5.49378	-0.73564
H	11.49079	-4.46757	-1.07617
H	10.28843	-5.76763	-0.98287
H	11.98118	-6.14771	-1.31232
C	11.32491	-5.00145	5.893175
H	11.10978	-6.03283	5.595022
H	11.3933	-4.98241	6.985581
H	12.30844	-4.7282	5.497108
C	8.889984	-4.46655	6.069718
H	8.608995	-5.47214	5.739558
H	8.071013	-3.78913	5.81151
H	8.978475	-4.48076	7.161872
C	10.59559	-2.61128	5.929422
H	9.83114	-1.87393	5.66847
H	11.54372	-2.27675	5.495869
H	10.70135	-2.60978	7.020148
C	11.79638	5.261338	-0.7601
C	10.45291	3.685843	-5.40653
C	11.48198	5.126095	0.74182
H	11.61344	4.098511	1.095779
H	10.45904	5.439102	0.974308
H	12.16057	5.762763	1.318579
C	13.26859	4.840706	-0.98233
H	13.93772	5.457302	-0.37164
H	13.57279	4.954645	-2.02691
H	13.42283	3.793477	-0.70234
C	11.62327	6.747117	-1.15504
H	11.87568	6.922563	-2.20487
H	12.27569	7.382268	-0.54527
H	10.59009	7.07497	-1.0009
C	11.89301	3.219941	-5.72711
H	12.64346	3.849895	-5.24058
H	12.07506	3.258935	-6.80704
H	12.05434	2.189963	-5.39274
C	10.26273	5.139039	-5.90209
H	10.42865	5.197631	-6.98369
H	10.96148	5.829711	-5.42108
H	9.247622	5.493439	-5.69519
C	9.474449	2.784276	-6.18288
H	8.432346	3.078017	-6.02088
H	9.579934	1.731243	-5.90288
H	9.675663	2.86147	-7.25619
C	0.388976	12.19133	2.512699
C	-1.46933	12.55218	-2.25099
C	-2.36754	13.75603	-1.87978
H	-2.55649	14.37711	-2.76264
H	-1.90513	14.39186	-1.1192
H	-3.33331	13.41794	-1.49003
C	-2.17385	11.75642	-3.36598
H	-3.15107	11.3817	-3.04481
H	-1.57408	10.9037	-3.70001
H	-2.33771	12.40411	-4.23327
C	-0.12271	13.07107	-2.80885
H	0.411963	13.6858	-2.07887
H	-0.2907	13.68456	-3.70124
H	0.5316	12.23863	-3.0875
C	0.7418	13.6742	2.291577
H	1.432318	13.80917	1.452595
H	1.229611	14.07353	3.186562
H	-0.14914	14.28223	2.103448
C	1.699307	11.42563	2.813767
H	2.389871	11.48375	1.965855
H	1.514951	10.36799	3.022931
H	2.199971	11.85547	3.688655
C	-0.55066	12.09867	3.738428
H	-0.80631	11.06286	3.979046
H	-1.48522	12.63939	3.556607

H	-0.07055	12.53694	4.620648	C	-10.3266	7.566572	-1.02479
C	-10.0243	6.352651	3.905695	C	-10.822	8.995371	-0.69812
C	-10.5041	7.758858	4.336912	H	-11.4322	9.018688	0.209555
H	-11.0037	7.709239	5.310959	H	-11.4336	9.384215	-1.52015
H	-11.2128	8.185612	3.621216	H	-9.97861	9.677823	-0.55067
H	-9.65976	8.450503	4.423369	C	-11.5457	6.637762	-1.23607
C	-9.0867	5.810559	5.001133	H	-12.1642	7.002582	-2.06388
H	-8.21425	6.45543	5.147344	H	-12.179	6.586523	-0.34557
H	-8.73052	4.801218	4.771174	H	-11.2242	5.619038	-1.47593
H	-9.62367	5.761484	5.953879	C	-9.53558	7.62512	-2.34527
C	-11.2428	5.404745	3.804329	H	-9.18108	6.636058	-2.65268
H	-11.974	5.756354	3.070538	H	-8.66967	8.291098	-2.27322
H	-11.7512	5.332385	4.772396	H	-10.1799	8.007083	-3.14365
H	-10.9316	4.39743	3.508797				

Cartesian coordinates of optimized geometry of **CP3** (xyz format; number of atoms: 309)

MMM conformer, E(RB3LYP) = -11572.7641282 a.u.

C	1.219108	-7.25661	-1.42917	C	-5.85129	6.11572	-0.40216
C	1.758056	-6.00757	-1.35945	N	-4.53502	6.072056	-0.00245
C	0.721661	-5.12923	-0.87802	C	-4.20387	7.345117	0.402114
N	-0.43411	-5.83684	-0.66955	C	0.307755	6.249739	1.348417
C	-0.1566	-7.14968	-0.99646	C	-0.50833	7.337749	1.423339
C	-4.4403	-8.73706	0.256223	C	-1.82167	6.913328	0.991436
C	-3.27964	-9.23645	-0.25377	N	-1.78469	5.573183	0.659491
C	-2.37064	-8.12529	-0.40138	C	-0.49512	5.15486	0.864693
N	-2.99104	-6.96326	-0.00259	C	-2.94249	7.751804	0.884222
C	-4.26025	-7.31231	0.399042	C	-1.29853	0.559312	-0.04117
C	-1.04934	-8.2272	-0.8835	C	-0.16665	1.404422	0.020781
C	-5.56681	-2.85738	1.345657	C	-0.69477	2.759911	0.178601
C	-6.10157	-4.10797	1.419386	N	-2.05349	2.750777	-0.00886
C	-5.07725	-5.03328	0.988085	C	-2.44823	1.450788	-0.19772
N	-3.93445	-4.3315	0.657803	C	0.009856	3.874551	0.620583
C	-4.21674	-3.00554	0.863183	C	-6.89167	2.573832	-1.43618
C	-5.24347	-6.42298	0.879798	C	-6.07973	1.482409	-1.36562
C	0.165578	-1.40421	-0.04075	C	-4.80169	1.94029	-0.88147
C	-1.13224	-0.84647	0.020756	N	-4.83689	3.294843	-0.67197
C	-2.0422	-1.98146	0.178495	C	-6.11198	3.711269	-1.00081
N	-1.35498	-3.15368	-0.00846	C	-6.59907	5.022921	-0.88704
C	-0.03167	-2.84568	-0.19678	C	-3.71713	1.092224	-0.63991
C	0.913541	-3.76569	-0.63762	Zn	-3.30578	4.427417	-0.00652
C	-3.36001	-1.92826	0.619862	C	5.674663	4.683272	-1.43403
Zn	-2.18106	-5.07649	-0.00613	C	4.323526	4.525596	-1.36338
C	-5.34838	8.212819	0.260624	C	4.081284	3.189306	-0.88062
C	-6.36026	7.457735	-0.2518	N	5.272081	2.542341	-0.67226

C	6.270105	3.438794	-1.0005
C	9.787327	0.524165	0.253708
C	9.639192	1.778475	-0.25753
C	8.222342	2.009737	-0.40514
N	7.526443	0.891719	-0.00513
C	8.463542	-0.03237	0.397284
C	7.649674	3.204497	-0.88807
C	5.259278	-3.3909	1.347382
C	6.609687	-3.22848	1.420849
C	6.898657	-1.87908	0.988289
N	5.719383	-1.24074	0.657558
C	4.712336	-2.14812	0.863884
C	8.185215	-1.32808	0.879419
C	1.133923	0.845504	-0.04096
C	1.299893	-0.5573	0.02101
C	2.737861	-0.77764	0.17868
N	3.409286	0.4036	-0.00898
C	2.48079	1.395459	-0.19753
C	2.804632	2.67383	-0.6392
C	3.350936	-1.94515	0.620647
Zn	5.487487	0.649727	-0.00769
H	1.70228	-8.17259	-1.73412
H	2.768069	-5.70049	-1.5973
H	-5.33899	-9.27939	0.508489
H	-3.05433	-10.2623	-0.50301
H	-6.03924	-1.91285	1.581585
H	-7.09815	-4.3872	1.726387
H	1.900327	-3.38842	-0.86786
H	-3.76547	-0.95233	0.848396
H	-5.36981	9.261622	0.515469
H	-7.36155	7.775222	-0.50079
H	1.361957	6.186752	1.584396
H	-0.25219	8.340158	1.731483
H	1.057755	3.737881	0.849279
H	-7.92589	2.613827	-1.74324
H	-6.31842	0.454378	-1.60475
H	-3.88348	0.049056	-0.87064
H	6.226202	5.559382	-1.74015
H	3.552461	5.24654	-1.60149
H	10.70647	0.017455	0.506351
H	10.41483	2.486451	-0.50757
H	4.677647	-4.27216	1.584172
H	7.349903	-3.95159	1.728386
H	1.984339	3.339575	-0.86941
H	2.708638	-2.78418	0.849899
C	9.346024	-2.18465	1.289788
C	10.22398	-2.70222	0.323319
C	9.564518	-2.48036	2.636311
C	11.30857	-3.50365	0.686308
H	10.0332	-2.46712	-0.71671
C	10.64406	-3.28111	3.045245
H	8.879691	-2.06995	3.371632
C	11.49555	-3.7765	2.053994
H	12.33475	-4.39662	2.344459
C	8.578125	4.309922	-1.29531
C	9.301786	5.020074	-0.32879
C	8.725767	4.643656	-2.64759
C	10.17051	6.057229	-0.68979
H	9.167183	4.749502	0.711414
C	9.585084	5.673049	-3.05143
H	8.159648	4.078496	-3.378
C	10.29138	6.359389	-2.05363
H	10.96118	7.160561	-2.35004
C	-2.78134	9.184826	1.296479
C	-2.63196	9.519908	2.643239
C	-2.77482	10.20551	0.331614
C	-2.4781	10.85462	3.053978
H	-2.64313	8.720452	3.377311
C	-2.62318	11.54502	0.696439
H	-2.88503	9.924355	-0.70864
C	-2.47751	11.84128	2.064276
H	-2.35997	12.87771	2.356123
C	-8.02037	5.274467	-1.29524
C	-8.99919	5.540185	-0.32912
C	-8.38075	5.24128	-2.64831
C	-10.3315	5.773386	-0.69132
H	-8.6993	5.554659	0.711656
C	-9.70152	5.470693	-3.05337
H	-7.60664	5.038108	-3.37831
C	-10.6512	5.732843	-2.05593
H	-11.6797	5.911994	-2.35331
C	-6.5663	-6.99956	1.288861
C	-7.45302	-7.49971	0.321233
C	-6.93265	-7.04201	2.635073
C	-8.68996	-8.0379	0.68277
H	-7.15313	-7.45134	-0.71851
C	-8.16656	-7.57612	3.042555
H	-6.23501	-6.65541	3.371257
C	-9.02083	-8.06443	2.050167
H	-9.97798	-8.48079	2.339473

C	-0.5561	-9.58432	-1.28935
C	-0.30481	-10.5658	-0.32203
C	-0.33897	-9.87974	-2.64118
C	0.158939	-11.8371	-0.68179
H	-0.47323	-10.3134	0.717812
C	0.122805	-11.139	-3.04379
H	-0.54384	-9.1071	-3.37224
C	0.362014	-12.0936	-2.04523
H	0.72069	-13.0746	-2.34067
C	10.84653	-3.57544	4.544452
C	12.28866	-4.09279	-0.3471
C	12.07763	-4.46204	4.810223
H	11.99159	-5.4379	4.321151
H	12.17677	-4.64081	5.885685
H	13.00361	-3.98794	4.468673
C	9.599762	-4.3062	5.097149
H	9.445059	-5.25996	4.581799
H	8.690689	-3.71009	4.976595
H	9.722104	-4.51442	6.166048
C	11.03955	-2.24394	5.308505
H	10.17612	-1.58168	5.198632
H	11.9207	-1.70694	4.942354
H	11.17852	-2.43532	6.378526
C	13.7194	-3.58489	-0.04882
H	14.42982	-3.99966	-0.77281
H	14.05609	-3.87519	0.950657
H	13.76818	-2.49293	-0.11341
C	11.93013	-3.69087	-1.79026
H	11.95559	-2.60537	-1.93011
H	10.93665	-4.04907	-2.0786
H	12.6533	-4.12993	-2.48508
C	12.26504	-5.6373	-0.25893
H	12.5559	-5.99419	0.733365
H	12.96078	-6.07136	-0.98588
H	11.26412	-6.02578	-0.47338
C	10.97797	6.862896	0.347132
C	12.48971	6.706745	0.057522
H	13.07821	7.278515	0.783911
H	12.75369	7.066946	-0.94108
H	12.79469	5.657311	0.124997
C	10.71628	6.389809	1.789584
H	10.9967	5.341297	1.932735
H	9.66472	6.503667	2.071884
H	11.31134	6.98838	2.486806
C	10.59063	8.357851	0.254352
H	10.79475	8.771641	-0.73751
H	11.15981	8.945282	0.983648
H	9.525017	8.49929	0.462121
C	9.775897	6.063261	-4.5305
C	11.26073	5.880392	-4.92471
H	11.92412	6.502229	-4.31648
H	11.41368	6.157773	-5.97379
H	11.57289	4.838407	-4.80013
C	9.371919	7.543387	-4.72953
H	9.507173	7.838102	-5.7763
H	9.97458	8.217638	-4.11404
H	8.320916	7.701011	-4.46608
C	8.919881	5.202422	-5.47871
H	7.850023	5.307423	-5.27169
H	9.178254	4.140777	-5.41102
H	9.087758	5.517105	-6.51374
C	-2.32212	11.17489	4.553423
C	-2.6064	12.69011	-0.33512
C	-3.76154	13.67388	-0.03224
H	-3.7599	14.4978	-0.75477
H	-3.67626	14.10887	0.967805
H	-4.73142	13.16962	-0.0953
C	-1.25731	13.44272	-0.24908
H	-1.09159	13.87178	0.74343
H	-1.23156	14.26333	-0.97493
H	-0.42048	12.77102	-0.46649
C	-2.77851	12.18094	-1.7787
H	-3.73123	11.65967	-1.9169
H	-1.97173	11.50085	-2.0703
H	-2.76239	13.02794	-2.47214
C	-2.16713	12.68377	4.821053
H	-1.27928	13.09658	4.330944
H	-2.0597	12.85729	5.896578
H	-3.0405	13.25045	4.481968
C	-1.06645	10.45789	5.103971
H	-0.16312	10.79976	4.587932
H	-1.13004	9.37276	4.982573
H	-0.94576	10.66678	6.172925
C	-3.57179	10.67727	5.318038
H	-3.71557	9.598829	5.206442
H	-4.47692	11.17405	4.953633
H	-3.47404	10.89138	6.388314
C	-11.4354	6.062645	0.34507
C	-10.1322	5.44733	-4.53331
C	-10.8972	6.067635	1.788428

H	-10.1314	6.83594	1.936105
H	-10.4682	5.099848	2.067276
H	-11.7148	6.278732	2.485243
C	-12.0592	7.449572	0.060573
H	-12.8501	7.668219	0.786933
H	-12.5021	7.501261	-0.93834
H	-11.3049	8.240027	0.132361
C	-12.5334	4.977268	0.2457
H	-12.992	4.950259	-0.74711
H	-13.3286	5.171202	0.974453
H	-12.1209	3.983881	0.449857
C	-10.7202	6.824808	-4.92115
H	-11.5924	7.082189	-4.31312
H	-11.035	6.823196	-5.97083
H	-9.97682	7.617962	-4.79093
C	-11.208	4.354867	-4.74007
H	-11.5289	4.329132	-5.78755
H	-12.0949	4.533397	-4.12513
H	-10.8161	3.365785	-4.48123
C	-8.956	5.145466	-5.48111
H	-8.50922	4.166752	-5.27867
H	-8.16834	5.902208	-5.40782
H	-9.31057	5.138037	-6.51682
C	-8.52387	-7.60577	4.54145
C	-9.68966	-8.59077	-0.35191
C	-9.96547	-10.0841	-0.05552
H	-10.6794	-10.4911	-0.78047
H	-10.3859	-10.2316	0.943495
H	-9.04421	-10.6723	-0.1201
C	-9.16139	-8.4796	-1.7946
H	-8.23407	-9.04443	-1.93448
H	-8.97445	-7.43983	-2.08159
H	-9.90282	-8.88539	-2.49039
C	-11.0154	-7.79802	-0.26363
H	-11.4707	-7.87273	0.728222
H	-11.7387	-8.18243	-0.99163
H	-10.851	-6.7367	-0.47651
C	-9.90841	-8.2269	4.805319

H	-10.7093	-7.66269	4.316255
H	-10.1137	-8.22436	5.880618
H	-9.9621	-9.2654	4.462528
C	-8.53167	-6.16145	5.096256
H	-9.27848	-5.54854	4.580715
H	-7.55989	-5.67366	4.977967
H	-8.77478	-6.1645	6.164783
C	-7.46914	-8.44138	5.305122
H	-6.46315	-8.02631	5.196266
H	-7.4462	-9.47257	4.937755
H	-7.70502	-8.4669	6.374979
C	0.45098	-12.9389	0.356052
C	-0.44034	-14.1697	0.0658
H	-0.241	-14.9649	0.792985
H	-0.25903	-14.5791	-0.93227
H	-1.50164	-13.9085	0.131416
C	0.170431	-12.4749	1.797917
H	-0.87787	-12.1929	1.939544
H	0.794824	-11.6213	2.080635
H	0.389968	-13.2893	2.495816
C	1.939234	-13.3517	0.265401
H	2.196642	-13.7358	-0.72601
H	2.162038	-14.1382	0.995237
H	2.594679	-12.4998	0.47376
C	0.367279	-11.5001	-4.52234
C	-0.5339	-12.694	-4.91737
H	-0.3288	-13.5793	-4.3083
H	-0.36876	-12.9659	-5.96604
H	-1.59234	-12.4424	-4.79459
C	1.851085	-11.8916	-4.71905
H	2.04016	-12.156	-5.76557
H	2.132019	-12.751	-4.10333
H	2.513357	-11.0608	-4.45435
C	0.052171	-10.3285	-5.47146
H	0.678345	-9.45484	-5.26368
H	-0.99631	-10.0206	-5.4056
H	0.242209	-10.6317	-6.50607

Cartesian coordinates of optimized geometry of **CP3** (xyz format; number of atoms: 309)

PPP conformer, E(RB3LYP) = -11572.7641394 a.u.

C	6.58304	-3.28072	-1.43261
C	5.231373	-3.43252	-1.3592
C	4.694432	-2.18585	-0.87452

N	5.708667	-1.28675	-0.66743
C	6.882777	-1.93425	-0.99851
C	9.653141	1.697892	0.255066

C	9.791232	0.44301	-0.25763	C	-5.58837	-2.81545	-1.35751
C	8.462933	-0.10191	-0.40393	C	-4.24001	-2.97344	-0.87341
N	7.533211	0.829739	-0.00158	N	-3.96829	-4.30123	-0.6658
C	8.238098	1.941162	0.400765	C	-5.11617	-4.99455	-0.99608
C	8.17397	-1.3945	-0.88801	C	-3.35439	-9.2092	0.257694
C	4.359613	4.489763	1.355626	C	-4.51038	-8.70177	-0.25501
C	5.711907	4.636098	1.428126	C	-4.31873	-7.27888	-0.40121
C	6.297487	3.386739	0.99488	N	-3.04709	-6.93911	0.000916
N	5.292308	2.498886	0.664881	C	-2.43659	-8.10504	0.403322
C	4.106703	3.155718	0.872144	C	-5.29408	-6.38265	-0.88507
C	7.675103	3.140332	0.884316	C	1.709529	-6.01935	1.356956
C	1.295133	-0.56778	-0.03102	C	1.160253	-7.26366	1.430252
C	1.140425	0.836299	0.030919	C	-0.21456	-7.14652	0.996977
C	2.491713	1.375334	0.187844	N	-0.48105	-5.8323	0.666264
N	3.412163	0.376004	-0.00045	C	0.680524	-5.13371	0.873029
C	2.731244	-0.79977	-0.18846	C	-1.11656	-8.21657	0.886916
C	3.334727	-1.97204	-0.63091	C	-1.1392	-0.83768	-0.03121
C	2.825963	2.651011	0.629662	C	0.154155	-1.40562	0.030727
Zn	5.492275	0.605168	-0.00133	C	-0.05442	-2.84535	0.187941
C	-6.29855	7.50959	0.254229	N	-1.38004	-3.14305	-0.00011
C	-5.28013	8.257533	-0.25569	C	-2.05802	-1.9656	-0.18836
C	-4.14352	7.380166	-0.40093	C	-3.37506	-1.90255	-0.63063
N	-4.48579	6.108636	-0.00053	C	0.883521	-3.7723	0.629878
C	-5.80146	6.162566	0.399632	Zn	-2.22135	-5.0592	0.000151
C	-6.06889	1.528983	1.352553	H	7.317666	-4.00924	-1.74093
C	-6.87231	2.626596	1.424243	H	4.642641	-4.30885	-1.59676
C	-6.08307	3.758775	0.99209	H	10.43453	2.398726	0.507401
N	-4.81101	3.33277	0.663488	H	10.70628	-0.07143	-0.50945
C	-4.78679	1.977554	0.870486	H	3.594387	5.217115	1.59306
C	-6.55885	5.074858	0.881304	H	6.270118	5.507528	1.735347
C	-0.1557	1.40549	-0.03086	H	2.685573	-2.80574	-0.86039
C	-1.2943	0.569387	0.03065	H	2.011167	3.323627	0.859387
C	-2.43687	1.469938	0.187508	H	-7.29681	7.835255	0.504904
N	-2.03174	2.766839	-0.00018	H	-5.29213	9.307442	-0.50651
C	-0.67298	2.76522	-0.18789	H	-6.31601	0.502475	1.589628
C	-3.70889	1.121158	0.628617	H	-7.90643	2.673904	1.730415
C	-0.44953	7.343161	-1.42568	H	-3.88382	0.079112	0.858024
C	0.357537	6.24823	-1.35372	H	-0.18551	8.344127	-1.73199
C	-0.45407	5.159198	-0.87133	H	1.410964	6.17682	-1.59076
N	-1.73992	5.587805	-0.66439	H	1.087111	3.729343	-0.85865
C	-1.76584	6.92893	-0.99329	H	-7.13109	-4.33409	-1.73763
C	-2.87897	7.777123	-0.88257	H	-6.05314	-1.86758	-1.59529
C	0.040504	3.874429	-0.62919	H	-3.13773	-10.2362	0.509942
Zn	-3.27054	4.453588	-0.00056	H	-5.41317	-9.23738	-0.50695
C	-6.13268	-4.062	-1.43003	H	2.722021	-5.72004	1.594162

H	1.63589	-8.18255	1.738141
H	-3.77267	-0.92371	-0.86058
H	1.873372	-3.40262	0.859265
C	-0.63552	-9.57669	1.297078
C	-0.39471	-10.5667	0.330327
C	-0.41709	-9.87236	2.643612
C	0.056894	-11.8375	0.693032
H	-0.56347	-10.3154	-0.70971
C	0.035746	-11.138	3.052229
H	-0.61177	-9.09832	3.37921
C	0.263149	-12.0962	2.060699
H	0.613014	-13.0793	2.350935
C	-6.62109	-6.94843	-1.29583
C	-7.51363	-7.43526	-0.33223
C	-6.9801	-6.99039	-2.64904
C	-8.75808	-7.9634	-0.69713
H	-7.21813	-7.38689	0.708771
C	-8.21419	-7.51179	-3.05678
H	-6.27265	-6.61247	-3.3771
C	-9.07904	-7.9889	-2.06181
H	-10.0399	-8.3958	-2.36111
C	-7.97832	5.338015	1.288072
C	-8.34686	5.29759	2.633759
C	-8.95381	5.62346	0.318703
C	-9.67042	5.538292	3.038909
H	-7.58091	5.079946	3.371394
C	-10.2811	5.867626	0.677936
H	-8.64918	5.643257	-0.7206
C	-10.6116	5.819393	2.044835
H	-11.6387	6.008034	2.332397
C	-2.70473	9.209887	-1.2907
C	-2.6811	10.22436	-0.32504
C	-2.56014	9.544471	-2.64311
C	-2.51615	11.5669	-0.68706
H	-2.78795	9.942168	0.715296
C	-2.39422	10.87476	-3.04795
H	-2.58574	8.744331	-3.37284
C	-2.37626	11.86032	-2.051
H	-2.24819	12.8965	-2.34813
C	8.612895	4.236953	1.293612
C	9.349433	4.940073	0.326192
C	8.760588	4.574347	2.639973
C	10.22442	5.966626	0.688065
H	9.215583	4.667872	-0.71368
C	9.63068	5.599289	3.047775

H	8.187798	4.01912	3.376025
C	10.34617	6.274992	2.055565
H	11.0229	7.069583	2.345077
C	9.327149	-2.26087	-1.29957
C	10.1947	-2.79168	-0.33638
C	9.543097	-2.5494	-2.65306
C	11.27426	-3.60509	-0.70197
H	10.00497	-2.56098	0.704833
C	10.61163	-3.35721	-3.06146
H	8.862262	-2.12488	-3.38081
C	11.45707	-3.86873	-2.06692
H	12.28991	-4.49709	-2.36683
C	0.257783	-11.4177	4.551453
C	0.33635	-12.9461	-0.34072
C	0.753149	-12.8518	4.816756
H	1.712658	-13.0495	4.327735
H	0.895667	-12.9985	5.892195
H	0.033037	-13.6024	4.47485
C	1.315235	-10.4339	5.106286
H	2.273783	-10.5591	4.591812
H	1.005548	-9.39173	4.986663
H	1.478418	-10.6121	6.175151
C	-1.07403	-11.2205	5.313869
H	-1.46035	-10.2033	5.203353
H	-1.84088	-11.9104	4.94668
H	-0.932	-11.4086	6.384077
C	-0.56496	-14.1679	-0.04299
H	-0.37434	-14.9677	-0.76742
H	-0.38476	-14.5749	0.956243
H	-1.62407	-13.8976	-0.10732
C	0.055936	-12.4858	-1.78375
H	-0.99015	-12.1948	-1.92344
H	0.687286	-11.6392	-2.07187
H	0.26594	-13.3051	-2.47882
C	1.821216	-13.3718	-0.25249
H	2.078303	-13.7536	0.73985
H	2.03466	-14.1636	-0.97937
H	2.483499	-12.5267	-0.467
C	-9.76662	-8.50256	0.336522
C	-10.0568	-9.99384	0.044119
H	-10.777	-10.3913	0.768189
H	-10.4759	-10.1401	-0.95572
H	-9.14191	-10.5914	0.113002
C	-9.24151	-8.39201	1.780472
H	-8.3206	-8.966	1.925215

H	-9.0447	-7.3533	2.064682	C	-2.47999	12.70787	0.348882
H	-9.9893	-8.78775	2.475244	C	-2.23803	11.27934	-4.52722
C	-11.0837	-7.69646	0.241424	C	-2.64951	12.19508	1.791525
H	-11.5363	-7.76974	-0.75178	H	-3.60724	11.68409	1.933388
H	-11.8134	-8.07093	0.968182	H	-1.84878	11.50491	2.076015
H	-10.9092	-6.63616	0.451235	H	-2.61987	13.03925	2.487949
C	-8.63928	-7.57818	-4.53705	C	-3.62532	13.70608	0.056607
C	-8.89758	-9.05101	-4.93402	H	-3.61018	14.52698	0.782412
H	-9.68794	-9.50434	-4.32863	H	-3.54054	14.14415	-0.94216
H	-9.20461	-9.11471	-5.98405	H	-4.60067	13.21294	0.122813
H	-7.99282	-9.65446	-4.80773	C	-1.12279	13.44487	0.257422
C	-9.93596	-6.75863	-4.73831	H	-0.95819	13.87538	-0.73467
H	-10.2544	-6.79986	-5.78606	H	-1.08287	14.26254	0.985935
H	-10.7587	-7.13883	-4.12582	H	-0.2925	12.7627	0.467104
H	-9.78004	-5.70796	-4.47251	C	-3.38552	12.23852	-4.92322
C	-7.56324	-7.01	-5.48167	H	-3.38528	13.14799	-4.3153
H	-7.35155	-5.9564	-5.27284	H	-3.28633	12.53927	-5.97232
H	-6.62363	-7.56744	-5.41211	H	-4.35983	11.75466	-4.7995
H	-7.91043	-7.0773	-6.51772	C	-0.88087	11.99512	-4.7256
C	-10.0275	5.487285	4.537277	H	-0.75661	12.29348	-5.77268
C	-11.3784	6.17873	-0.35871	H	-0.80089	12.89688	-4.11153
C	-11.9867	7.570504	-0.06385	H	-0.04818	11.33566	-4.4599
H	-12.7732	7.804458	-0.79019	C	-2.28104	10.06534	-5.47446
H	-12.4313	7.619035	0.934414	H	-1.4738	9.35582	-5.26603
H	-11.223	8.352575	-0.12745	H	-3.23298	9.528935	-5.40738
C	-12.4897	5.105687	-0.27205	H	-2.16479	10.40207	-6.50963
H	-12.9516	5.075126	0.719103	C	9.763136	5.931839	4.546818
H	-13.2803	5.315788	-1.00128	C	11.04398	6.762769	-0.34639
H	-12.0884	4.109389	-0.48429	C	12.55303	6.593325	-0.04975
C	-10.8363	6.190016	-1.80048	H	13.14976	7.158223	-0.7748
H	-10.0612	6.950682	-1.93927	H	12.81623	6.953117	0.94918
H	-10.4175	5.219801	-2.0865	H	12.84854	5.540965	-0.11406
H	-11.6493	6.416587	-2.49774	C	10.78469	6.289567	-1.78917
C	-11.5176	5.776392	4.79853	H	11.05598	5.238203	-1.92895
H	-12.1678	5.04419	4.308624	H	9.735529	6.412619	-2.07651
H	-11.7186	5.72753	5.873522	H	11.3885	6.881254	-2.48475
H	-11.8062	6.775137	4.454883	C	10.67014	8.261537	-0.25814
C	-9.70631	4.079905	5.094127	H	10.87289	8.675279	0.734001
H	-10.293	3.312234	4.578742	H	11.24862	8.842253	-0.9855
H	-8.64876	3.826476	4.977392	H	9.607013	8.41243	-0.47197
H	-9.94499	4.02864	6.162426	C	10.75852	7.077142	4.811011
C	-9.19229	6.542428	5.300875	H	10.4502	8.007226	4.322349
H	-8.11799	6.367776	5.193572	H	10.81561	7.273886	5.886392
H	-9.4048	7.551199	4.932001	H	11.76807	6.828096	4.46802
H	-9.4292	6.514717	6.370451	C	8.383097	6.357006	5.102391

H	8.012377	7.249578	4.587556
H	7.634874	5.568082	4.983859
H	8.456745	6.588144	6.171041
C	10.25788	4.67982	5.309437
H	9.56935	3.837144	5.199987
H	11.2382	4.359667	4.941612
H	10.3508	4.897332	6.379459
C	12.24501	-4.21056	0.331167
C	13.68216	-3.7178	0.038934
H	14.38599	-4.14411	0.762644
H	14.01785	-4.00735	-0.96111
H	13.74352	-2.62681	0.108542
C	11.88735	-3.81152	1.775389
H	11.92474	-2.72707	1.920575
H	10.88924	-4.15988	2.059735
H	12.60383	-4.26204	2.46978
C	12.20359	-5.75413	0.235135
H	12.49295	-6.10919	-0.75829
H	12.89218	-6.20014	0.961664
H	11.19762	-6.13205	0.444667
C	10.88197	-3.69068	-4.54201
C	12.28662	-3.17734	-4.93813
H	13.07429	-3.6352	-4.33262
H	12.49581	-3.41079	-5.9882
H	12.35643	-2.09211	-4.81127
C	10.82093	-5.22325	-4.74479
H	11.01594	-5.4773	-5.79278
H	11.56169	-5.74613	-4.13281
H	9.833159	-5.61407	-4.47942
C	9.852008	-3.04217	-5.48624
H	8.833667	-3.38559	-5.27762
H	9.865155	-1.94977	-5.41603
H	10.08389	-3.3086	-6.52245

Cartesian coordinates of optimized geometry of **CP4** (xyz format; number of atoms: 412)

MMMM conformer, E(RB3LYP) = -15430.1242744 a.u.

C	1.745986	-7.46413	1.944336
C	0.802255	-6.49049	1.909515
C	1.405523	-5.3205	1.233742
N	2.689014	-5.58508	0.878579
C	2.968171	-6.93432	1.307644
C	7.458488	-6.87305	-0.23128
C	6.592022	-7.72773	0.424645
C	5.330508	-7.04459	0.54005
N	5.425411	-5.77744	-0.03014
C	6.7261	-5.66919	-0.52226
C	4.152141	-7.59568	1.146158
C	6.078642	-1.28694	-2.26565
C	7.048844	-2.23559	-2.27719
C	6.549071	-3.40341	-1.5262
N	5.204333	-3.10792	-1.09745
C	4.94058	-1.83872	-1.50088
C	7.249854	-4.53958	-1.2316
C	0.449306	-1.74295	0.071945
C	1.522031	-0.7353	-0.05552
C	2.732063	-1.56229	-0.44742
N	2.542707	-2.89534	-0.0632
C	1.209638	-3.03085	0.339613
C	0.688795	-4.11671	0.982503
C	3.778229	-1.08202	-1.17843
Zn	3.976638	-4.35713	-0.08179
H	1.679058	-8.46013	2.347093
H	-0.20638	-6.50696	2.27516
H	8.48581	-7.04503	-0.48569
H	6.793283	-8.71426	0.793124
H	6.079393	-0.30706	-2.70237
H	8.023756	-2.20379	-2.73217
H	-0.35204	-4.10506	1.317973
H	3.780965	-0.03925	-1.50796
C	1.59963	0.584419	0.177531
C	0.637231	1.701323	0.085961
C	1.523598	2.910922	-0.16022
N	2.847327	2.621889	0.192863
C	2.906452	1.266559	0.533704
C	7.369629	1.379583	2.119257
C	6.297149	0.549009	2.13615
C	5.184447	1.262033	1.473304
N	5.568129	2.510561	1.102509
C	6.960335	2.634941	1.459497
C	3.927022	0.654234	1.201043
C	7.381228	7.014335	-0.31414
C	8.174253	6.049103	0.278016
C	7.331822	4.912462	0.539608
N	6.033155	5.176497	0.106362

C	6.052174	6.470151	-0.4109
C	7.764655	3.702156	1.174739
C	1.437876	6.451547	-1.59204
C	2.478484	7.320682	-1.6354
C	3.662368	6.639967	-1.07483
N	3.256936	5.313766	-0.67587
C	1.939749	5.199252	-0.98553
C	1.107507	4.068115	-0.75191
C	4.919012	7.163439	-0.95359
Zn	4.438366	3.919409	0.187322
H	8.363273	1.211466	2.496876
H	6.212374	-0.44593	2.528708
H	3.825941	-0.38875	1.514443
H	7.674473	7.989074	-0.65106
H	9.218467	6.108455	0.513399
H	0.42227	6.589342	-1.90957
H	2.499702	8.334117	-1.99739
H	0.064799	4.179641	-1.06307
C	-6.19704	1.55067	-2.42088
C	-7.07267	2.57729	-2.55804
C	-6.57403	3.709513	-1.75156
N	-5.33101	3.300848	-1.14333
C	-5.11256	2.017415	-1.52899
C	-0.69841	1.755993	0.206806
C	-1.77578	0.76505	-0.01151
C	-2.97281	1.626376	-0.372
N	-2.77264	2.936647	0.072058
C	-1.4601	3.020638	0.557257
C	-4.01778	1.196714	-1.1385
C	-2.05178	7.290743	2.534212
C	-1.13655	6.288203	2.509491
C	-1.68784	5.215744	1.655564
N	-2.91852	5.557444	1.19505
C	-3.19823	6.877996	1.703135
C	-0.9701	4.025962	1.337187
C	-7.36491	7.237187	-0.54331
C	-6.56608	7.988902	0.298119
C	-5.39503	7.202854	0.585115
N	-5.48043	5.973869	-0.06954
C	-6.6842	5.989527	-0.76912
C	-7.18485	4.918982	-1.58379
C	-4.30667	7.625299	1.416275
Zn	-4.13958	4.455938	0.014125
H	-6.22793	0.567568	-2.84941
H	-7.98245	2.622513	-3.13173

H	-4.05523	0.157891	-1.47744
H	-2.00548	8.238493	3.042155
H	-0.17923	6.229904	2.989889
H	0.048917	3.964383	1.729922
H	-8.31652	7.502746	-0.95988
H	-6.75796	8.970696	0.68425
C	-7.35931	-7.26811	-0.27985
C	-8.08759	-6.44265	0.556362
C	-7.30005	-5.25764	0.772421
N	-6.09236	-5.36238	0.085595
C	-6.11864	-6.59643	-0.5637
C	-1.7207	-6.15768	-2.33315
C	-2.74501	-7.04484	-2.4083
C	-3.86972	-6.50683	-1.61916
N	-3.45981	-5.23092	-1.08599
C	-2.18045	-5.03122	-1.49412
C	-5.06681	-7.12402	-1.38322
C	-1.85553	-0.56942	0.115422
C	-0.88365	-1.67049	-0.05936
C	-1.75947	-2.85919	-0.40664
N	-3.07312	-2.62611	0.020412
C	-3.15253	-1.29195	0.432718
C	-1.34918	-3.92654	-1.14982
C	-7.40913	-1.77921	2.469204
C	-6.43388	-0.84519	2.338608
C	-5.36723	-1.4409	1.504405
N	-5.69699	-2.7072	1.142022
C	-6.99127	-2.97971	1.718055
C	-7.70973	-4.13104	1.561651
C	-4.1804	-0.74294	1.144162
Zn	-4.59584	-3.99398	0.040098
H	-7.64182	-8.22912	-0.6624
H	-9.05544	-6.62544	0.979707
H	-0.74248	-6.20963	-2.77039
H	-2.78741	-7.98861	-2.92405
H	-0.31444	-3.98397	-1.4995
H	-8.34269	-1.71566	3.001123
H	-6.38739	0.150283	2.735693
H	-4.12753	0.301627	1.463192
C	8.676819	-4.60944	-1.65882
C	9.059488	-5.45053	-2.71017
C	9.619087	-3.80979	-0.99784
C	10.40055	-5.49672	-3.11238
H	8.300572	-6.06014	-3.19891
C	10.96116	-3.83666	-1.3952

H	9.288203	-3.17806	-0.17441
C	11.33471	-4.68522	-2.44911
H	12.37929	-4.71559	-2.7603
C	4.244774	-9.00981	1.610371
C	4.283813	-9.29788	2.980047
C	4.283561	-10.0368	0.658062
C	4.35076	-10.629	3.410828
H	4.26809	-8.4747	3.692725
C	4.364455	-11.3717	1.072834
H	4.252829	-9.77539	-0.39929
C	4.390556	-11.6502	2.448382
H	4.445334	-12.6882	2.778097
C	-9.03075	-4.23633	2.245597
C	-10.2089	-4.20125	1.489091
C	-9.07608	-4.36197	3.640348
C	-11.4516	-4.30197	2.127126
H	-10.1377	-4.09871	0.406399
C	-10.3117	-4.44284	4.294526
H	-8.14061	-4.40109	4.196978
C	-11.486	-4.41327	3.525713
H	-12.4513	-4.48087	4.029145
C	-5.30383	-8.45969	-2.002
C	-5.34877	-9.59911	-1.18896
C	-5.46656	-8.56245	-3.38934
C	-5.5689	-10.8584	-1.7607
H	-5.21265	-9.48498	-0.11392
C	-5.66154	-9.81764	-3.9791
H	-5.44415	-7.65536	-3.99105
C	-5.7137	-10.9515	-3.15334
H	-5.87221	-11.931	-3.60616
C	5.149867	8.56555	-1.40529
C	5.069436	8.881181	-2.76756
C	5.440056	9.554818	-0.4561
C	5.261988	10.20243	-3.19146
H	4.863582	8.086285	-3.48336
C	5.663657	10.87433	-0.86729
H	5.492082	9.275343	0.596175
C	5.563246	11.18241	-2.23338
H	5.726176	12.21062	-2.5586
C	9.211423	3.604922	1.523136
C	9.61754	3.650745	2.863217
C	10.14766	3.442031	0.494339
C	10.97332	3.51447	3.185837
H	8.864886	3.795599	3.636786
C	11.5096	3.321148	0.800221
H	9.799337	3.411092	-0.53831
C	11.90309	3.347897	2.146398
H	12.95995	3.237829	2.393184
C	-4.39733	9.00305	1.979864
C	-4.22663	10.10219	1.122803
C	-4.64077	9.192345	3.341181
C	-4.30112	11.40122	1.631348
H	-4.03937	9.918126	0.065378
C	-4.71141	10.4946	3.869562
H	-4.78078	8.328027	3.989244
C	-4.53564	11.58109	3.008204
H	-4.58056	12.5984	3.39677
C	-8.47855	5.162769	-2.28406
C	-9.64166	4.531621	-1.82538
C	-8.51274	6.015375	-3.39458
C	-10.8577	4.74293	-2.48734
H	-9.5818	3.887262	-0.94939
C	-9.72291	6.24457	-4.0609
H	-7.58889	6.49162	-3.72055
C	-10.8814	5.600596	-3.59813
H	-11.8262	5.772902	-4.11486
C	-12.7693	-4.26851	1.345162
C	-10.4206	-4.57848	5.817337
C	-10.9818	-5.97879	6.135087
H	-11.0678	-6.13656	7.214277
H	-11.9752	-6.12852	5.70089
H	-10.3306	-6.76432	5.736083
C	-11.3727	-3.4906	6.3555
H	-11.4335	-3.52191	7.447897
H	-11.0285	-2.49028	6.071823
H	-12.3903	-3.60908	5.970635
C	-9.05981	-4.41733	6.520049
H	-8.34582	-5.18669	6.205749
H	-8.61318	-3.4382	6.315691
H	-9.16571	-4.5041	7.607034
C	-13.6807	-5.41674	1.824097
H	-13.9656	-5.30415	2.874827
H	-14.6054	-5.4589	1.240329
H	-13.1795	-6.38517	1.720741
C	-13.4455	-2.90951	1.614112
H	-12.807	-2.07996	1.291513
H	-14.3947	-2.8253	1.076608
H	-13.6552	-2.7642	2.678519
C	-12.5517	-4.43122	-0.17047
H	-11.9567	-3.61252	-0.58838

H	-12.0392	-5.37083	-0.40422
H	-13.5058	-4.43986	-0.70693
C	-5.83949	-9.98705	-5.49176
C	-7.30484	-10.3871	-5.75704
H	-7.49527	-10.5139	-6.82728
H	-7.56582	-11.3279	-5.2624
H	-7.99609	-9.62233	-5.38556
C	-4.88825	-11.0913	-5.99746
H	-3.84799	-10.8626	-5.74246
H	-5.12567	-12.0679	-5.56406
H	-4.94663	-11.1973	-7.08531
C	-5.52645	-8.69305	-6.26632
H	-6.20079	-7.87668	-5.98551
H	-4.49884	-8.35675	-6.09151
H	-5.63888	-8.84336	-7.34549
C	-5.62297	-12.1335	-0.91183
C	-4.31009	-12.9091	-1.14244
H	-4.28609	-13.8338	-0.55738
H	-4.17946	-13.1807	-2.19475
H	-3.44029	-12.3099	-0.8506
C	-6.82878	-12.9938	-1.34332
H	-6.7362	-13.3413	-2.37721
H	-6.92871	-13.882	-0.71108
H	-7.76375	-12.4274	-1.26862
C	-5.76984	-11.8252	0.589989
H	-4.91189	-11.2659	0.978571
H	-6.67205	-11.2373	0.792891
H	-5.8433	-12.7468	1.176811
C	4.397056	-11.0052	4.895785
C	4.400263	-12.5323	0.072619
C	5.561701	-13.4818	0.431401
H	5.424912	-13.945	1.413535
H	5.652105	-14.2919	-0.29934
H	6.516832	-12.9454	0.451002
C	3.055323	-13.2812	0.164871
H	3.018365	-14.1197	-0.53735
H	2.88216	-13.6836	1.167798
H	2.216353	-12.6163	-0.06855
C	4.605475	-12.0498	-1.37541
H	3.783889	-11.4085	-1.7123
H	5.538014	-11.4846	-1.48247
H	4.656963	-12.8954	-2.06918
C	5.780173	-11.6186	5.19259
H	5.87566	-11.8921	6.24778
H	5.961524	-12.5215	4.601466
H	6.583467	-10.9107	4.959565
C	3.285928	-12.0337	5.190937
H	2.301507	-11.6404	4.915686
H	3.432915	-12.9665	4.637724
H	3.254598	-12.2892	6.254768
C	4.185407	-9.78942	5.816908
H	4.966597	-9.03369	5.678728
H	3.216655	-9.31062	5.638054
H	4.209934	-10.0858	6.871162
C	-4.966	10.66557	5.371209
C	-4.10993	12.63416	0.740914
C	-5.08521	12.14467	5.785671
H	-5.27408	12.23599	6.861158
H	-4.16646	12.70101	5.572557
H	-5.91358	12.64205	5.269842
C	-3.78647	10.03379	6.138117
H	-3.69714	8.960963	5.938214
H	-2.83595	10.49919	5.856392
H	-3.90641	10.15312	7.219678
C	-6.2823	9.950995	5.737088
H	-7.1208	10.34214	5.150474
H	-6.23026	8.873354	5.54904
H	-6.52667	10.08368	6.795582
C	-2.75491	13.27535	1.102118
H	-2.55773	14.1597	0.488485
H	-2.71959	13.58747	2.150606
H	-1.93024	12.57161	0.943842
C	-5.2553	13.63478	0.997206
H	-5.24965	14.01085	2.025141
H	-5.17998	14.50237	0.334107
H	-6.23138	13.16859	0.823264
C	-4.1113	12.27816	-0.75747
H	-3.28565	11.60774	-1.01868
H	-5.0465	11.79036	-1.05385
H	-4.00316	13.17535	-1.37569
C	-9.82043	7.162361	-5.28449
C	-12.1621	4.086051	-2.02263
C	-13.0717	5.190041	-1.44684
H	-13.3096	5.952692	-2.1949
H	-14.0183	4.777341	-1.08489
H	-12.589	5.699926	-0.6054
C	-11.9263	3.018092	-0.93822
H	-11.2683	2.218654	-1.29573
H	-11.4765	3.446678	-0.03563
H	-12.8692	2.55067	-0.6347

C	-12.8486	3.406615	-3.22539
H	-13.7578	2.880408	-2.91772
H	-13.1375	4.129373	-3.99495
H	-12.1847	2.673375	-3.69585
C	-8.49811	7.895829	-5.57559
H	-8.18658	8.517247	-4.72862
H	-7.6849	7.1963	-5.79755
H	-8.59766	8.557414	-6.44285
C	-10.9151	8.21996	-5.03425
H	-10.9806	8.928007	-5.86648
H	-11.904	7.766875	-4.91455
H	-10.706	8.795239	-4.12549
C	-10.1873	6.297394	-6.50747
H	-11.1507	5.794783	-6.37779
H	-10.2532	6.902875	-7.41679
H	-9.43496	5.520008	-6.68098
C	5.180869	10.59822	-4.66976
C	6.614864	10.87299	-5.16413
H	6.624253	11.15688	-6.22084
H	7.090053	11.6821	-4.60043
H	7.247684	9.985264	-5.05359
C	4.317377	11.86768	-4.8181
H	3.32015	11.71785	-4.39132
H	4.765046	12.72989	-4.31386
H	4.189374	12.13915	-5.87055
C	4.553108	9.489813	-5.53553
H	5.149231	8.570607	-5.51683
H	3.540605	9.241324	-5.19924
H	4.480276	9.802075	-6.58305
C	5.994581	11.99121	0.129004
C	7.234827	12.76459	-0.36497
H	7.051409	13.26769	-1.31941
H	7.535477	13.53323	0.35441
H	8.088314	12.09168	-0.50643
C	4.777917	12.9354	0.208739
H	4.955012	13.75286	0.914946
H	4.547048	13.38341	-0.76278
H	3.882398	12.39849	0.541145
C	6.298741	11.44697	1.537097
H	5.436998	10.92644	1.968707
H	7.141756	10.74684	1.524447
H	6.560994	12.25771	2.225038
C	11.47695	3.541299	4.633273
C	12.41036	4.757181	4.801166
H	12.77793	4.837369	5.828788
H	13.284	4.696178	4.144982
H	11.88821	5.690308	4.562037
C	10.32988	3.656463	5.65412
H	9.751108	4.575373	5.512471
H	9.639922	2.808911	5.586244
H	10.71599	3.675874	6.678616
C	12.24878	2.235676	4.915567
H	13.13251	2.135242	4.27791
H	12.59128	2.194347	5.954318
H	11.61699	1.358457	4.740661
C	12.56178	3.118396	-0.29577
C	13.81338	3.966759	0.00931
H	14.30484	3.654029	0.936454
H	14.55359	3.883953	-0.79348
H	13.55512	5.026902	0.113006
C	12.03415	3.531213	-1.68254
H	11.1837	2.916632	-1.99731
H	11.70956	4.57796	-1.69156
H	12.80866	3.42303	-2.4487
C	12.93248	1.621755	-0.31655
H	13.6868	1.410372	-1.08043
H	13.33463	1.293104	0.646843
H	12.05771	0.998626	-0.53505
C	10.8795	-6.39541	-4.25784
C	12.03042	-2.98283	-0.7045
C	12.8174	-2.19277	-1.77055
H	12.14641	-1.57869	-2.3811
H	13.54917	-1.52292	-1.30752
H	13.36593	-2.85352	-2.4489
C	12.97644	-3.92953	0.061226
H	13.47419	-4.63758	-0.60864
H	13.75595	-3.37193	0.589135
H	12.42762	-4.51774	0.805495
C	11.4239	-1.9745	0.289178
H	10.72387	-1.29127	-0.20437
H	10.88689	-2.47648	1.101577
H	12.20326	-1.36017	0.753037
C	9.753667	-7.28292	-4.8199
H	9.336434	-7.94204	-4.05053
H	8.933709	-6.68593	-5.23361
H	10.12371	-7.92342	-5.62768
C	11.41063	-5.49501	-5.39178
H	12.25859	-4.88477	-5.0662
H	11.74501	-6.08982	-6.24759
H	10.63348	-4.80991	-5.74809

C	12.00505	-7.31171	-3.73537
H	12.34573	-8.00422	-4.51154
H	12.87763	-6.7412	-3.40273
H	11.66185	-7.91057	-2.88436

Cartesian coordinates of optimized geometry of **CP4** (xyz format; number of atoms: 412)

MMMP conformer, E(RB3LYP) = -15430.1965754 a.u.

C	7.130445	-1.94432	-2.47956
C	6.293113	-0.95367	-2.00508
C	5.00592	-1.56412	-1.78116
N	5.043343	-2.90211	-2.14484
C	6.356836	-3.15892	-2.55376
C	5.567771	-7.77195	-3.40292
C	6.625934	-6.91915	-3.39563
C	6.130341	-5.59392	-2.99279
N	4.72008	-5.70775	-2.73554
C	4.380384	-6.99598	-2.99922
C	6.866385	-4.43834	-2.92237
C	-0.25771	-6.31834	-2.17761
C	0.545399	-7.31556	-2.59094
C	1.950672	-6.79696	-2.6133
N	1.933956	-5.4401	-2.21296
C	0.605255	-5.128	-1.89136
C	3.051559	-7.53938	-2.93924
C	1.554057	-0.7971	-0.14903
C	0.509742	-1.68109	-0.20902
C	0.973818	-2.87762	-0.96314
N	2.290206	-2.76012	-1.26939
C	2.701466	-1.44237	-0.86092
C	3.90159	-0.88966	-1.17451
C	0.144318	-3.96944	-1.32882
Zn	3.498502	-4.19442	-2.10488
H	8.166233	-1.86128	-2.74593
H	6.526907	0.075052	-1.82011
H	5.536479	-8.81677	-3.6496
H	7.655862	-7.11526	-3.63651
H	-1.3236	-6.30572	-2.04176
H	0.301817	-8.3254	-2.87476
H	4.089273	0.16669	-0.92716
H	-0.92926	-3.85454	-1.15641
C	-0.80136	-1.56191	0.372984
C	-1.67083	-0.46711	0.304
C	-2.88309	-0.87372	1.005479
N	-2.78079	-2.17701	1.427262
C	-1.48177	-2.60559	1.096005
C	-2.05094	-7.0059	2.785549
C	-1.02547	-6.20163	2.395148
C	-1.59321	-4.88748	2.064908
N	-3.00611	-4.93388	2.332128
C	-3.28386	-6.20086	2.727596
C	-0.92033	-3.84209	1.500402
C	-7.94647	-5.4029	3.24149
C	-7.10491	-6.45057	3.310053
C	-5.72852	-5.95419	2.997032
N	-5.80974	-4.56089	2.712672
C	-7.14107	-4.19205	2.879295
C	-4.59869	-6.71678	3.016689
C	-6.38616	0.418439	2.029834
C	-7.40649	-0.38891	2.389757
C	-6.87931	-1.78356	2.450801
N	-5.55185	-1.79393	2.141379
C	-5.18746	-0.43523	1.816107
C	-4.00248	-0.02278	1.315899
C	-7.66777	-2.91636	2.766793
Zn	-4.28033	-3.36087	2.164876
H	-2.02984	-8.03692	3.087548
H	0.020138	-6.43539	2.311291
H	0.162429	-3.96029	1.324742
H	-9.00985	-5.35827	3.405164
H	-7.30437	-7.48261	3.544519
H	-6.37703	1.484513	1.883849
H	-8.43223	-0.1467	2.608066
H	-3.85822	1.051474	1.128718
C	-1.07901	6.816416	0.846497
C	-1.96828	7.765677	0.504423
C	-3.02966	7.12202	-0.33511
N	-2.72451	5.748917	-0.46976
C	-1.52978	5.525924	0.231882
C	-1.49826	0.846672	-0.25096
C	-0.51262	1.814915	-0.08702
C	-1.23629	3.117545	-0.23445
N	-2.45242	2.935683	-0.81511
C	-2.64328	1.519424	-0.92719

C	-0.84316	4.349519	0.353182
C	-6.63632	1.851712	-3.4549
C	-5.76506	0.881867	-2.99641
C	-4.73563	1.560877	-2.25257
N	-4.96917	2.933006	-2.25876
C	-6.1422	3.122994	-2.98997
C	-3.66243	0.903318	-1.58355
C	-6.24637	7.828913	-2.28956
C	-6.98268	6.897868	-2.94708
C	-6.33243	5.58937	-2.75017
N	-5.15156	5.798699	-1.95232
C	-5.1059	7.12516	-1.66656
C	-4.10788	7.775785	-0.86666
C	-6.78075	4.383368	-3.21452
Zn	-3.8148	4.352552	-1.39909
H	-0.18176	6.893827	1.435246
H	-1.98593	8.814888	0.74706
H	0.033596	4.334838	1.00947
H	-7.52276	1.717157	-4.04359
H	-5.81878	-0.1764	-3.14906
H	-3.68811	-0.1975	-1.6023
H	-6.4041	8.888299	-2.20309
H	-7.88328	7.025363	-3.52217
C	8.158507	5.363717	3.153804
C	7.716025	6.399577	2.389983
C	6.505714	5.953485	1.689242
N	6.230796	4.603506	2.098363
C	7.233589	4.239346	2.938556
C	5.841709	-0.30644	3.194091
C	6.823932	0.46235	3.69856
C	6.612006	1.862262	3.210403
N	5.447712	1.877716	2.398098
C	4.944	0.574921	2.38084
C	7.419853	2.924367	3.497924
C	0.889191	1.662939	0.165207
C	1.660943	0.535066	0.387713
C	2.911764	0.995998	1.064359
N	3.02351	2.346657	0.981167
C	1.870741	2.801633	0.244397
C	3.80599	0.13967	1.74799
C	3.854024	6.964548	-0.8698
C	2.759865	6.195681	-1.21669
C	2.858655	4.966532	-0.46982
N	4.002636	4.9688	0.305336
C	4.624629	6.208626	0.085638
C	5.796163	6.66497	0.750188
C	1.856385	3.940238	-0.48934
Zn	4.664339	3.463924	1.466313
H	9.011502	5.315976	3.804378
H	8.136476	7.383103	2.277615
H	5.665549	-1.36019	3.310344
H	7.651178	0.195978	4.334811
H	3.551389	-0.92148	1.782674
H	4.107933	7.945593	-1.22191
H	1.973329	6.439239	-1.90052
H	1.010511	4.142739	-1.16462
C	2.897446	-8.98281	-3.25144
C	3.223162	-9.93335	-2.27454
C	2.426771	-9.3833	-4.5087
C	3.085896	-11.2989	-2.55167
H	3.581129	-9.58952	-1.30574
C	2.266318	-10.7448	-4.79427
H	2.19811	-8.62097	-5.25111
C	2.60069	-11.6877	-3.80955
H	2.481483	-12.7491	-4.02836
C	8.324289	-4.51474	-3.23074
C	8.798186	-4.07321	-4.47177
C	9.200488	-5.02944	-2.26716
C	10.16766	-4.13939	-4.7583
H	8.087967	-3.67983	-5.19817
C	10.57071	-5.11559	-2.54456
H	8.800283	-5.35019	-1.30622
C	11.03682	-4.66486	-3.78905
H	12.10377	-4.7218	-4.00856
C	6.28323	8.026157	0.381211
C	6.115434	9.094314	1.269773
C	6.902022	8.215187	-0.86172
C	6.577717	10.37071	0.92177
H	5.617177	8.917971	2.222145
C	7.353452	9.48786	-1.2308
H	7.018025	7.360146	-1.52714
C	7.187516	10.5511	-0.32866
H	7.539891	11.54443	-0.6099
C	8.588105	2.734843	4.395442
C	9.874286	2.638144	3.846738
C	8.394706	2.653589	5.780297
C	10.98314	2.472171	4.68503
H	9.989908	2.695656	2.765446
C	9.492005	2.464198	6.630456
H	7.385739	2.747526	6.178325

C	10.77588	2.380858	6.070447
H	11.63484	2.243315	6.728001
C	-9.1245	-2.71534	2.96306
C	-9.6088	-2.24498	4.191041
C	-10.0047	-2.9976	1.90991
C	-10.9828	-2.04266	4.370739
H	-8.90069	-2.04546	4.992869
C	-11.3834	-2.8194	2.080676
H	-9.59802	-3.3483	0.962812
C	-11.8551	-2.33661	3.310503
H	-12.9263	-2.18483	3.446769
C	-4.69898	-8.1682	3.319778
C	-5.10732	-9.05188	2.312013
C	-4.38711	-8.63816	4.601896
C	-5.21754	-10.4214	2.5843
H	-5.32844	-8.65717	1.32171
C	-4.47361	-10.0075	4.883864
H	-4.08002	-7.92478	5.364806
C	-4.89368	-10.8824	3.869616
H	-4.96862	-11.9488	4.085919
C	-8.05245	4.357065	-3.99372
C	-9.27476	4.436758	-3.30901
C	-8.01875	4.253437	-5.38535
C	-10.4761	4.415578	-4.02326
H	-9.26617	4.504341	-2.22206
C	-9.21922	4.231006	-6.11774
H	-7.05924	4.185951	-5.89776
C	-10.4321	4.304078	-5.42561
H	-11.3737	4.270726	-5.97416
C	-4.28437	9.226698	-0.60502
C	-3.52126	10.16148	-1.31739
C	-5.20847	9.645858	0.361419
C	-3.67005	11.52941	-1.05807
H	-2.82187	9.803313	-2.07048
C	-5.38018	11.01125	0.620587
H	-5.78077	8.894223	0.901981
C	-4.60204	11.93749	-0.09083
H	-4.72419	13.00147	0.113116
C	6.395159	11.57462	1.852407
C	8.035237	9.750069	-2.57801
C	9.52043	10.06594	-2.30851
H	10.06218	10.25203	-3.24108
H	9.640105	10.95097	-1.67607
H	10.01529	9.231685	-1.79899
C	7.35405	10.9503	-3.26759
H	7.775937	11.12829	-4.26199
H	6.279503	10.77508	-3.39108
H	7.472827	11.87498	-2.69446
C	7.946525	8.538351	-3.52407
H	8.457136	7.660726	-3.11242
H	6.905701	8.258529	-3.72449
H	8.414253	8.757382	-4.49013
C	7.690034	12.41208	1.889238
H	7.929246	12.84047	0.910381
H	7.603376	13.24525	2.593848
H	8.545246	11.8025	2.200448
C	5.234122	12.42253	1.294895
H	4.305468	11.84229	1.252894
H	5.047537	13.30199	1.918458
H	5.442508	12.77481	0.279518
C	6.063295	11.14624	3.294275
H	5.116336	10.598	3.349957
H	6.84683	10.50517	3.712566
H	5.967766	12.01654	3.952078
C	9.326749	2.388533	8.15216
C	9.770701	3.744605	8.736997
H	9.676225	3.758929	9.826879
H	10.81407	3.967425	8.491453
H	9.161502	4.564347	8.33997
C	10.20506	1.255661	8.722089
H	9.962538	0.295748	8.253634
H	11.27127	1.442742	8.558076
H	10.0581	1.144682	9.801059
C	7.868053	2.112489	8.563195
H	7.194997	2.915317	8.242221
H	7.500744	1.175399	8.131017
H	7.773212	2.030598	9.651011
C	12.40556	2.352201	4.126867
C	12.81799	0.868549	4.209945
H	13.83053	0.714702	3.824894
H	12.79588	0.50059	5.240664
H	12.14068	0.237103	3.624292
C	13.3712	3.216246	4.96413
H	13.43835	2.868379	5.999948
H	14.38361	3.195314	4.548758
H	13.04385	4.261367	4.98792
C	12.49545	2.82073	2.662164
H	11.87669	2.206354	1.998698
H	12.1702	3.861331	2.554043
H	13.52354	2.758836	2.290196

C	10.73906	-3.68025	-6.10403
C	11.57089	-5.65088	-1.51416
C	12.55039	-6.62838	-2.1956
H	13.15962	-6.13297	-2.95832
H	13.23898	-7.07043	-1.4686
H	12.01291	-7.44834	-2.6842
C	12.34136	-4.4453	-0.93947
H	13.0721	-4.76044	-0.18851
H	12.88263	-3.90128	-1.72013
H	11.66004	-3.7325	-0.46091
C	10.87369	-6.39942	-0.3624
H	10.20408	-5.7428	0.204252
H	10.28249	-7.244	-0.73235
H	11.60533	-6.79936	0.347567
C	11.17799	-4.93549	-6.88489
H	11.59092	-4.67075	-7.86312
H	11.94397	-5.50177	-6.34607
H	10.33223	-5.61101	-7.05419
C	11.95095	-2.758	-5.85931
H	11.67378	-1.90176	-5.23433
H	12.77001	-3.28108	-5.35603
H	12.34698	-2.36536	-6.80115
C	9.707158	-2.90351	-6.94228
H	8.838767	-3.52104	-7.19593
H	9.344546	-2.01567	-6.41202
H	10.1435	-2.56138	-7.88673
C	-9.15305	4.093619	-7.64265
C	-11.8355	4.470464	-3.31702
C	-10.5172	4.343598	-8.31347
H	-10.4393	4.25812	-9.40288
H	-11.2698	3.617968	-7.98834
H	-10.8944	5.347628	-8.09267
C	-8.69008	2.659638	-7.96744
H	-7.71128	2.439695	-7.52882
H	-9.39356	1.917334	-7.57744
H	-8.60675	2.503133	-9.04659
C	-8.14677	5.116331	-8.207
H	-8.40951	6.136545	-7.90826
H	-7.12697	4.924561	-7.85612
H	-8.12113	5.087145	-9.30089
C	-12.4402	3.052591	-3.35542
H	-13.4158	3.021654	-2.86061
H	-12.5788	2.699491	-4.38243
H	-11.7886	2.331346	-2.84936
C	-12.7645	5.461707	-4.04736

H	-12.9821	5.141289	-5.07155
H	-13.7236	5.561711	-3.52915
H	-12.3124	6.457911	-4.10341
C	-11.7102	4.926093	-1.85051
H	-11.1092	4.228908	-1.2564
H	-11.2478	5.916199	-1.77609
H	-12.6932	4.988417	-1.37183
C	-6.36341	11.51596	1.682545
C	-2.86515	12.59335	-1.81264
C	-3.8354	13.35745	-2.7364
H	-4.63181	13.85043	-2.17019
H	-3.31352	14.12881	-3.31083
H	-4.31571	12.67955	-3.4508
C	-1.74038	11.98035	-2.66788
H	-1.03436	11.40948	-2.05513
H	-2.13496	11.30986	-3.43916
H	-1.16774	12.75928	-3.18261
C	-2.22356	13.56373	-0.79922
H	-1.59647	14.306	-1.30302
H	-2.97611	14.11191	-0.22374
H	-1.59127	13.02552	-0.08482
C	-7.34483	10.4187	2.136206
H	-7.92584	10.02788	1.293873
H	-6.82512	9.576005	2.605061
H	-8.05672	10.80633	2.872469
C	-7.18771	12.68906	1.113219
H	-7.93901	13.0316	1.831684
H	-6.5581	13.5503	0.867374
H	-7.7132	12.39361	0.198655
C	-5.54035	11.98956	2.897691
H	-4.85029	12.79585	2.62946
H	-6.18842	12.36147	3.69704
H	-4.93969	11.17172	3.311048
C	-11.5624	-1.54345	5.699685
C	-12.2731	-2.73212	6.38044
H	-12.7052	-2.44042	7.343142
H	-13.085	-3.12679	5.760992
H	-11.5748	-3.55634	6.566194
C	-12.5714	-0.40617	5.431629
H	-12.1034	0.413441	4.874541
H	-13.4319	-0.75007	4.848399
H	-12.9602	0.008506	6.367571
C	-10.4722	-1.00324	6.644674
H	-9.74476	-1.77798	6.913388
H	-9.92384	-0.17061	6.18968

H	-10.9087	-0.63458	7.579652
C	-12.374	-3.0956	0.944388
C	-13.6278	-3.80579	1.494251
H	-14.1755	-3.17758	2.204149
H	-14.3221	-4.06408	0.688425
H	-13.3604	-4.73311	2.011869
C	-12.7655	-1.73726	0.329289
H	-13.4762	-1.86223	-0.49312
H	-13.228	-1.07794	1.070681
H	-11.8876	-1.2153	-0.06759
C	-11.7619	-3.99086	-0.1495
H	-10.9014	-3.51433	-0.63225
H	-11.4273	-4.94993	0.259653
H	-12.4915	-4.2066	-0.93696
C	-5.6426	-11.4262	1.507513
C	-6.66532	-12.4213	2.093245
H	-7.02901	-13.1134	1.326775
H	-6.23367	-13.0272	2.896554
H	-7.53429	-11.8973	2.505615
C	-6.29178	-10.7356	0.293048
H	-7.17431	-10.1563	0.585061
H	-5.59524	-10.0562	-0.21051
H	-6.61583	-11.4707	-0.45162
C	-4.37864	-12.1788	1.045482
H	-3.9005	-12.7126	1.873206
H	-4.61407	-12.9144	0.270158
H	-3.63546	-11.4878	0.632076
C	-4.15585	-10.5697	6.273656
C	-5.49171	-10.9508	6.942743
H	-6.03502	-11.7033	6.362393
H	-5.3318	-11.3596	7.945141
H	-6.14758	-10.0791	7.041244
C	-3.42088	-9.54815	7.16106
H	-2.47925	-9.22423	6.704357
H	-4.02883	-8.65596	7.346373
H	-3.17772	-9.97833	8.138525
C	-3.25911	-11.8169	6.136554
H	-2.96841	-12.2057	7.117554
H	-3.76446	-12.6285	5.603507
H	-2.34062	-11.5832	5.587242
C	3.413424	-12.3705	-1.50606
C	1.766589	-11.2352	-6.15732
C	0.647662	-12.2768	-5.95228
H	-0.17302	-11.8611	-5.35835
H	0.23123	-12.6047	-6.90978
H	1.010315	-13.1696	-5.43332
C	2.958807	-11.8783	-6.89375
H	3.370993	-12.725	-6.33606
H	2.665429	-12.2446	-7.8819
H	3.770698	-11.1565	-7.03473
C	1.20287	-10.0911	-7.02017
H	0.368857	-9.58575	-6.52302
H	1.965379	-9.33847	-7.2453
H	0.830979	-10.4665	-7.97869
C	4.254689	-11.8094	-0.34409
H	5.196039	-11.3791	-0.70356
H	3.71942	-11.0287	0.207261
H	4.507733	-12.5948	0.37553
C	2.078066	-12.9065	-0.95116
H	1.459326	-13.3432	-1.74155
H	2.242945	-13.6794	-0.19453
H	1.493819	-12.1052	-0.48565
C	4.210466	-13.5171	-2.16147
H	4.508179	-14.2668	-1.42169
H	3.627205	-14.0341	-2.93027
H	5.122209	-13.1404	-2.63761

Cartesian coordinates of optimized geometry of **CP4** (xyz format; number of atoms: 412)

MMPP conformer, E(RB3LYP) = -15430.2560488 a.u.

C	-6.58701	-2.76718	3.220049
C	-5.22912	-2.82926	3.130539
C	-4.83932	-1.97436	2.037372
N	-5.95638	-1.4054	1.469758
C	-7.04257	-1.88693	2.164675
C	-10.1968	0.315388	-0.60356
C	-10.1951	-0.53244	0.461717
C	-8.81623	-0.72869	0.847317
N	-7.99929	0.005077	0.016309
C	-8.82007	0.660315	-0.87561
C	-8.38894	-1.58561	1.882178
C	-5.25275	2.866965	-3.08481
C	-6.61048	2.799956	-3.17254
C	-7.05301	1.840567	-2.18267

N	-5.96153	1.334115	-1.51545
C	-4.85189	1.952293	-2.04499
C	-8.39707	1.521959	-1.90757
C	-1.68934	-0.6442	0.283905
C	-1.69425	0.643127	-0.2882
C	-3.09205	0.879231	-0.64334
N	-3.91333	-0.01052	-0.00569
C	-3.0862	-0.89257	0.634721
C	-3.52283	-1.78257	1.619383
C	-3.53517	1.77177	-1.62249
Zn	-5.96731	-0.02006	-0.00866
H	-7.22777	-3.26762	3.930145
H	-4.5443	-3.39474	3.748627
H	-11.0485	0.6683	-1.16516
H	-11.0447	-1.00378	0.932318
H	-4.57624	3.486731	-3.65839
H	-7.26081	3.356774	-3.83016
H	-2.76552	-2.37545	2.1185
H	-2.7847	2.387341	-2.10404
C	-0.70358	1.720062	-0.24746
C	0.692333	1.725967	-0.24795
C	1.083862	3.102724	0.101538
N	-0.01469	3.907711	0.199702
C	-1.10649	3.0935	0.102221
C	-4.21479	6.571896	1.002795
C	-4.16687	5.21125	0.965007
C	-2.78894	4.844092	0.746923
N	-2.0092	5.974291	0.711891
C	-2.85748	7.049816	0.848806
C	-2.37963	3.5385	0.486127
C	0.639264	10.2351	0.776298
C	-0.72145	10.22938	0.776618
C	-1.13955	8.846499	0.694652
N	-0.03189	8.034877	0.614681
C	1.068935	8.855787	0.694089
C	-2.47175	8.403823	0.806486
C	4.126871	5.246096	0.962424
C	4.163383	6.607091	1.000181
C	2.801985	7.073585	0.847169
N	1.962676	5.990972	0.710813
C	2.751919	4.867371	0.745297
C	2.353442	3.558394	0.484716
C	2.404886	8.424312	0.805163
Zn	-0.02332	5.982391	0.53337
H	-5.08439	7.20185	1.113376
H	-4.9899	4.511837	1.0308
H	-3.15865	2.800763	0.587103
H	1.297235	11.08716	0.858519
H	-1.38652	11.07589	0.859137
H	4.955804	4.553636	1.027624
H	5.027737	7.244339	1.110074
H	3.138708	2.827229	0.585165
C	5.258738	-2.78663	3.12471
C	6.616197	-2.71366	3.212745
C	7.063474	-1.82939	2.15722
N	5.972675	-1.35633	1.463683
C	4.860896	-1.93451	2.032288
C	1.692034	0.657453	-0.28962
C	1.698398	-0.62998	0.28212
C	3.097616	-0.86678	0.631626
N	3.916796	0.022304	-0.00931
C	3.087526	0.905361	-0.64591
C	3.542464	-1.75338	1.615625
C	6.587581	2.858631	-3.17585
C	5.229368	2.913454	-3.08728
C	4.837161	1.994242	-2.04818
N	5.952489	1.385246	-1.51993
C	7.039132	1.901963	-2.18731
C	3.52232	1.802048	-1.62499
C	10.20294	-0.44848	0.451476
C	10.19662	0.400545	-0.61284
C	8.816826	0.734623	-0.88288
N	8.002392	0.071783	0.009153
C	8.826217	-0.65617	0.838522
C	8.407069	-1.51719	1.87328
C	8.385911	1.594072	-1.91345
Zn	5.970753	0.030398	-0.01365
H	4.579175	-3.35785	3.743315
H	7.261739	-3.20918	3.921955
H	2.790521	-2.35258	2.115307
H	7.232358	3.422037	-3.83326
H	4.547145	3.527845	-3.65987
H	2.766293	2.411461	-2.10567
H	11.05688	-0.91349	0.920514
H	11.04486	0.760808	-1.17504
C	-0.63572	-10.2296	-0.70843
C	0.724971	-10.2238	-0.70946
C	1.143058	-8.84028	-0.63834
N	0.035349	-8.02818	-0.56389
C	-1.0655	-8.8497	-0.63662

C	-4.12249	-5.24172	-0.93801
C	-4.15947	-6.60303	-0.96369
C	-2.7984	-7.0687	-0.80515
N	-1.95897	-5.98521	-0.67721
C	-2.74771	-4.86164	-0.72238
C	-2.40146	-8.41915	-0.75152
C	0.70724	-1.70654	0.248683
C	-0.68923	-1.71243	0.249532
C	-1.0805	-3.09175	-0.09017
N	0.018011	-3.89753	-0.182
C	1.109751	-3.08246	-0.0916
C	-2.34945	-3.5506	-0.47215
C	4.2172	-6.5673	-0.97039
C	4.168667	-5.20635	-0.94443
C	2.791053	-4.83802	-0.72646
N	2.011999	-5.96828	-0.68012
C	2.860419	-7.04456	-0.80959
C	2.475104	-8.39835	-0.75535
C	2.382029	-3.53046	-0.47544
Zn	0.026627	-5.97505	-0.49863
H	-1.29359	-11.0823	-0.78415
H	1.389976	-11.0709	-0.78618
H	-4.9509	-4.54931	-1.0103
H	-5.02391	-7.24091	-1.06923
H	-3.13439	-2.82042	-0.58115
H	5.08686	-7.19777	-1.07754
H	4.991029	-4.5069	-1.018
H	3.160555	-2.7936	-0.58552
C	-9.44989	2.156977	-2.76497
C	-10.3238	3.111329	-2.22765
C	-9.56647	1.803863	-4.11587
C	-11.3105	3.717498	-3.01468
H	-10.2112	3.37571	-1.18329
C	-10.5406	2.384197	-4.93731
H	-8.88433	1.060292	-4.50966
C	-11.3951	3.334671	-4.36086
H	-12.1552	3.794931	-4.98439
C	-9.44423	-2.23877	2.722092
C	-9.64307	-3.62478	2.665181
C	-10.2467	-1.4656	3.571713
C	-10.6265	-4.25037	3.440956
H	-9.01863	-4.20311	1.995304
C	-11.2398	-2.05153	4.365951
H	-10.0733	-0.39691	3.604272
C	-11.4059	-3.44102	4.279597
H	-12.172	-3.91079	4.888576
C	3.549029	-9.43892	-0.85773
C	3.862089	-10.2386	0.249194
C	4.245718	-9.62689	-2.05869
C	4.858738	-11.2196	0.178307
H	3.314322	-10.0734	1.169036
C	5.249418	-10.5966	-2.17228
H	3.981159	-9.00487	-2.90511
C	5.533247	-11.3747	-1.04096
H	6.308601	-12.1311	-1.11264
C	-3.46662	-9.46884	-0.8523
C	-4.164	-9.66222	-2.05201
C	-3.77066	-10.2717	0.254833
C	-5.15956	-10.6404	-2.16415
H	-3.9064	-9.03759	-2.89865
C	-4.75899	-11.2612	0.185369
H	-3.22255	-10.1023	1.173698
C	-5.43451	-11.4215	-1.03266
H	-6.2035	-12.1844	-1.1032
C	3.470156	9.473141	0.914035
C	4.165922	9.658688	2.115881
C	3.776005	10.28267	-0.18769
C	5.161707	10.63582	2.23543
H	3.906998	9.028761	2.958189
C	4.764772	11.27119	-0.11082
H	3.229118	10.11914	-1.10834
C	5.438607	11.42368	1.109147
H	6.207925	12.18572	1.185388
C	-3.54573	9.443701	0.915831
C	-3.85937	10.25007	-0.18601
C	-4.24195	9.623978	2.118211
C	-4.85634	11.23029	-0.10875
H	-3.31196	10.09067	-1.10708
C	-5.24579	10.59278	2.238177
H	-3.977	8.99664	2.960583
C	-5.53031	11.37774	1.111762
H	-6.30591	12.13336	1.188359
C	9.433486	2.239325	-2.76952
C	10.30404	3.199397	-2.2271
C	9.554	1.897901	-4.11807
C	11.28363	3.813717	-3.01008
H	10.1896	3.457309	-1.18134
C	10.52664	2.489581	-4.941
H	8.878087	1.151062	-4.52237
C	11.3736	3.439492	-4.36349

H	12.13113	3.910507	-4.97791
C	9.468565	-2.16234	2.71155
C	9.677982	-3.54677	2.654151
C	10.26639	-1.38312	3.560076
C	10.6674	-4.16486	3.428333
H	9.057004	-4.12981	1.985135
C	11.26512	-1.96147	4.352669
H	10.08473	-0.31581	3.593108
C	11.4419	-3.34962	4.265839
H	12.21252	-3.81355	4.873547
C	5.231264	-12.113	1.378087
C	6.031368	-10.8329	-3.47961
C	5.813426	-12.2888	-3.95532
H	6.366655	-12.4738	-4.88304
H	6.155892	-13.0162	-3.21346
H	4.753226	-12.4826	-4.14798
C	7.539545	-10.597	-3.22787
H	8.110712	-10.7655	-4.14781
H	7.72511	-9.56986	-2.89697
H	7.936144	-11.2705	-2.46249
C	5.581856	-9.88814	-4.61026
H	4.525491	-10.0266	-4.86186
H	5.735427	-8.83619	-4.34894
H	6.165379	-10.0919	-5.51386
C	5.004328	-13.5974	1.005724
H	5.61173	-13.9008	0.148031
H	5.269402	-14.2468	1.847709
H	3.954964	-13.7812	0.752911
C	6.720133	-11.8977	1.73923
H	6.908681	-10.8548	2.014188
H	7.001885	-12.5296	2.589132
H	7.382503	-12.1475	0.905257
C	4.38613	-11.7991	2.627135
H	4.525324	-10.7672	2.965047
H	3.31799	-11.9584	2.447747
H	4.68459	-12.4582	3.448741
C	-5.12158	-12.1583	1.385421
C	-4.88316	-13.6406	1.01178
H	-5.14112	-14.2926	1.853964
H	-5.48981	-13.9486	0.155187
H	-3.83284	-13.8156	0.756705
C	-6.61142	-11.9555	1.749777
H	-6.80799	-10.9144	2.025725
H	-7.27343	-12.2103	0.917021
H	-6.88619	-12.5902	2.599893
C	-4.27646	-11.8381	2.632874
H	-3.20741	-11.9884	2.451131
H	-4.42351	-10.8076	2.971704
H	-4.56769	-12.5002	3.454713
C	-5.94198	-10.8828	-3.47008
C	-7.45163	-10.6599	-3.2153
H	-8.02313	-10.8328	-4.1342
H	-7.84096	-11.3371	-2.44946
H	-7.64532	-9.63455	-2.88356
C	-5.71255	-12.3366	-3.94692
H	-6.04734	-13.0672	-3.20474
H	-6.26601	-12.5259	-4.87363
H	-4.65112	-12.5212	-4.14176
C	-5.50275	-9.93374	-4.60115
H	-5.66484	-8.88325	-4.3391
H	-4.44572	-10.063	-4.85483
H	-6.08622	-10.1421	-5.50374
C	-10.8758	-5.77101	3.397195
C	-12.135	-1.2313	5.315732
C	-13.6171	-1.40978	4.909096
H	-13.9306	-2.45672	4.957176
H	-14.268	-0.83636	5.578694
H	-13.7874	-1.05723	3.886557
C	-11.9391	-1.72832	6.767631
H	-12.5729	-1.1569	7.4552
H	-12.2	-2.78513	6.875898
H	-10.8984	-1.60726	7.085332
C	-11.8065	0.273198	5.276946
H	-10.7757	0.473661	5.586259
H	-11.9519	0.697269	4.278167
H	-12.4674	0.812015	5.963543
C	-12.3296	-6.04263	2.943016
H	-12.524	-7.12064	2.910576
H	-13.0601	-5.59359	3.622331
H	-12.5095	-5.63509	1.942879
C	-10.66	-6.36949	4.80742
H	-9.63454	-6.19977	5.151788
H	-11.3366	-5.92954	5.545833
H	-10.839	-7.45054	4.794396
C	-9.92727	-6.49261	2.421255
H	-10.0524	-6.13763	1.393255
H	-8.87695	-6.36345	2.701552
H	-10.1404	-7.5664	2.427246
C	10.62403	2.073568	-6.4218
C	12.25018	4.8754	-2.44948

C	11.74482	2.818354	-7.17109	H	13.80442	-0.94752	3.869633
H	11.77169	2.488531	-8.2146	C	11.96555	-1.63317	6.753335
H	11.58547	3.901646	-7.17251	H	12.23473	-2.68797	6.861085
H	12.72974	2.617398	-6.737	H	12.59592	-1.05698	7.440039
C	9.284206	2.382584	-7.13122	H	10.92443	-1.52014	7.072583
H	8.446875	1.849591	-6.67178	C	5.942472	10.86976	3.543887
H	9.05985	3.453763	-7.09359	C	5.713387	12.32081	4.029195
H	9.332008	2.083052	-8.18425	H	6.265671	12.50414	4.957814
C	10.91227	0.556091	-6.51161	H	6.049703	13.05568	3.291911
H	11.8601	0.308304	-6.02267	H	4.651808	12.50497	4.22366
H	10.12651	-0.03711	-6.03535	C	7.452316	10.64734	3.289853
H	10.97801	0.242029	-7.55944	H	7.645713	9.623872	2.952218
C	12.06546	6.201861	-3.22424	H	7.84314	11.32881	2.528583
H	12.74953	6.9667	-2.83948	H	8.022679	10.8144	4.210561
H	12.26783	6.082169	-4.29256	C	5.501004	9.914199	4.668601
H	11.04243	6.577188	-3.11821	H	4.443684	10.04264	4.9215
C	13.70814	4.384698	-2.61507	H	5.662782	8.865209	4.400434
H	13.96243	4.205345	-3.66374	H	6.08329	10.11676	5.57328
H	14.40855	5.132338	-2.226	C	5.129684	12.17524	-1.30494
H	13.871	3.450418	-2.06776	C	4.891209	13.65535	-0.92287
C	12.0079	5.157338	-0.95462	H	5.496619	13.95799	-0.06349
H	10.99947	5.54136	-0.77023	H	5.150779	14.31227	-1.76075
H	12.14933	4.261023	-0.3421	H	3.840543	13.82929	-0.66843
H	12.71778	5.912848	-0.60272	C	6.620025	11.97393	-1.66809
C	12.15548	-1.1345	5.301209	H	6.896375	12.61343	-2.51407
C	10.92818	-5.68356	3.384183	H	7.280828	12.22374	-0.83285
C	12.38345	-5.94427	2.92833	H	6.816729	10.9344	-1.94978
H	13.11134	-5.48973	3.60677	C	4.28644	11.86271	-2.55561
H	12.58588	-7.02079	2.895724	H	4.43366	10.83413	-2.90024
H	12.55907	-5.53545	1.927961	H	3.217153	12.01245	-2.37477
C	9.983948	-6.41226	2.409314	H	4.579338	12.52947	-3.37306
H	8.933009	-6.29114	2.6909	C	-5.22984	12.13067	-1.30298
H	10.10513	-6.05621	1.381209	C	-5.00298	13.61287	-0.92195
H	10.20528	-7.4844	2.414917	H	-5.26872	14.26722	-1.75992
C	10.71841	-6.28365	4.794656	H	-5.60995	13.91104	-0.06212
H	10.90544	-7.36335	4.781405	H	-3.9535	13.79545	-0.66868
H	11.39266	-5.83871	5.532264	C	-4.3854	11.82429	-2.55438
H	9.692183	-6.12152	5.140249	H	-3.31718	11.98285	-2.37477
C	11.81534	0.367443	5.263099	H	-4.5246	10.79434	-2.89824
H	11.95596	0.792708	4.264138	H	-4.6846	12.48815	-3.3719
H	10.78351	0.559959	5.574044	C	-6.71888	11.91709	-1.66447
H	12.47316	0.911257	5.948717	H	-7.38083	12.162	-0.8287
C	13.63826	-1.30152	4.892359	H	-7.00127	12.55386	-2.51052
H	14.28578	-0.72325	5.561104	H	-6.90745	10.87584	-1.94541
H	13.95982	-2.34604	4.939808	C	-6.0273	10.82084	3.547232

C	-5.80993	12.274	4.03161	H	-12.8985	2.165384	-6.40434
H	-6.1531	13.00565	3.29429	H	-12.2501	1.177438	-7.71947
H	-6.36284	12.45315	4.960672	H	-12.2849	0.542571	-6.06441
H	-4.74974	12.46714	4.224958	C	-9.67728	0.955001	-6.87847
C	-5.57681	9.869563	4.672003	H	-8.64718	1.306534	-6.76105
H	-5.73004	8.819128	4.404478	H	-9.78415	0.018677	-6.32142
H	-4.52037	10.00698	4.923865	H	-9.82895	0.727007	-7.93842
H	-6.15995	10.06768	5.57712	C	-12.0407	5.065954	-0.96428
C	-7.53546	10.58564	3.294702	H	-12.1757	4.171672	-0.34735
H	-8.10635	10.74838	4.215851	H	-11.0345	5.457264	-0.783
H	-7.93268	11.26342	2.53345	H	-12.755	5.818563	-0.61498
H	-7.72057	9.560409	2.957766	C	-12.1082	6.099519	-3.23868
C	-12.2833	4.775619	-2.45753	H	-12.3115	5.973383	-4.30611
C	-10.7008	2.013146	-6.42491	H	-12.7964	6.861905	-2.85651
C	-10.5029	3.276538	-7.2957	H	-11.0873	6.48179	-3.1363
H	-9.50203	3.697058	-7.15338	C	-13.7381	4.274565	-2.61894
H	-10.6196	3.030155	-8.35709	H	-14.4431	5.019208	-2.23239
H	-11.2299	4.057375	-7.05405	H	-13.9923	4.088795	-3.66654
C	-12.119	1.443201	-6.66444	H	-13.8942	3.341589	-2.06747

Cartesian coordinates of optimized geometry of **CP4** (xyz format; number of atoms: 412)

MPMP conformer, E(RB3LYP) = -15430.3521694 a.u.

C	0.800122	6.668739	3.713304	C	-0.46361	2.780042	1.31997
C	1.332638	5.794965	2.815	C	0.486576	3.743273	1.653968
C	0.327728	4.794283	2.556032	C	-3.5881	1.139583	1.679894
N	-0.7971	5.054527	3.30238	Zn	-2.5188	3.972479	3.28185
C	-0.5337	6.19792	4.022376	H	1.255851	7.555389	4.127404
C	-4.76526	6.252997	6.155115	H	2.309879	5.824621	2.351338
C	-3.61835	6.985412	6.148283	H	-5.63992	6.399658	6.770541
C	-2.73545	6.382241	5.176586	H	-3.37834	7.843929	6.757078
N	-3.35349	5.292033	4.604533	H	-6.23677	0.364049	2.405156
C	-4.59762	5.193012	5.187718	H	-7.34893	2.058833	4.180534
C	-1.42725	6.829928	4.905849	H	1.447229	3.675532	1.156072
C	-5.79515	1.241099	2.859707	H	-3.93464	0.236453	1.190399
C	-6.35646	2.097063	3.757632	C	-1.68796	-0.2659	-0.39303
C	-5.36598	3.111236	4.052079	C	-0.94475	-1.42857	-0.38293
N	-4.22402	2.865255	3.323691	C	-1.5856	-2.33338	-1.32393
C	-4.46708	1.730747	2.586258	N	-2.68897	-1.73762	-1.87753
C	-5.55621	4.192936	4.930762	C	-2.77087	-0.47939	-1.33994
C	-0.25943	1.689145	0.380201	C	-6.64062	0.759525	-3.77654
C	-1.42221	0.946073	0.387664	C	-5.77417	1.301126	-2.87657
C	-2.31786	1.595141	1.331817	C	-4.77513	0.299268	-2.60006
N	-1.71661	2.703001	1.870326	N	-5.02892	-0.83278	-3.33765

C	-6.16672	-0.57692	-4.06914
C	-3.73152	0.467226	-1.69107
C	-6.20242	-4.82847	-6.16217
C	-6.9355	-3.68201	-6.17202
C	-6.34071	-2.78965	-5.20379
N	-5.25483	-3.40161	-4.61717
C	-5.15039	-4.65108	-5.18787
C	-6.79127	-1.47923	-4.94902
C	-1.21748	-5.82476	-2.81599
C	-2.06556	-6.39478	-3.71591
C	-3.07767	-5.40761	-4.028
N	-2.83829	-4.25879	-3.30826
C	-1.70996	-4.49445	-2.55904
C	-1.1268	-3.60675	-1.656
C	-4.15195	-5.60661	-4.91379
Zn	-3.94646	-2.55372	-3.29179
H	-7.52422	1.210755	-4.20193
H	-5.80795	2.282746	-2.42254
H	-3.66814	1.432669	-1.20194
H	-6.34362	-5.70897	-6.77051
H	-7.78919	-3.44826	-6.78997
H	-0.34411	-6.26171	-2.34999
H	-2.02332	-7.39109	-4.12932
H	-0.22777	-3.94831	-1.15558
C	5.797811	-1.19255	2.877758
C	6.359687	-2.03161	3.79116
C	5.369665	-3.04051	4.104514
N	4.227348	-2.80833	3.372178
C	4.469791	-1.68754	2.6139
C	0.260388	-1.68664	0.410809
C	1.423155	-0.94358	0.403627
C	2.319639	-1.57523	1.358751
N	1.718871	-2.67306	1.917985
C	0.465412	-2.76017	1.37021
C	3.590109	-1.11329	1.697365
C	-0.79635	-6.60478	3.834895
C	-1.32951	-5.74739	2.921331
C	-0.32483	-4.75158	2.643436
N	0.800608	-4.99824	3.393481
C	0.537763	-6.12837	4.134418
C	-0.48447	-3.71715	1.722544
C	4.770942	-6.14352	6.264835
C	3.624162	-6.87614	6.272091
C	2.74051	-6.29082	5.290217
N	3.357932	-5.21115	4.697997

C	4.602416	-5.10128	5.278522
C	5.560561	-4.10583	5.002852
C	1.432136	-6.74358	5.028814
Zn	2.522256	-3.91654	3.351669
H	6.238974	-0.32386	2.407
H	7.352256	-1.98523	4.213034
H	3.936186	-0.21929	1.19107
H	-1.25192	-7.48376	4.265116
H	-2.30713	-5.78551	2.459087
H	-1.44556	-3.6585	1.224345
H	5.64607	-6.27886	6.88219
H	3.384794	-7.7235	6.896585
C	6.196561	4.709318	-6.25631
C	6.92936	3.56268	-6.24616
C	6.335682	2.688368	-5.26093
N	5.250755	3.311271	-4.68413
C	5.145833	4.550102	-5.27747
C	1.216208	5.767906	-2.92242
C	2.063398	6.32126	-3.8335
C	3.074835	5.328273	-4.12888
N	2.836004	4.192786	-3.3881
C	1.708593	4.442304	-2.64199
C	4.148014	5.510779	-5.01956
C	1.688159	0.253898	-0.39936
C	0.944993	1.416591	-0.40978
C	1.585025	2.304037	-1.36775
N	2.687768	1.698131	-1.91152
C	2.770107	0.449935	-1.35112
C	1.126144	3.571261	-1.72242
C	6.636772	-0.83408	-3.76919
C	5.771438	-1.35887	-2.85826
C	4.772892	-0.35193	-2.59895
N	5.025931	0.766345	-3.35752
C	6.162734	0.496869	-4.08566
C	6.786274	1.382717	-4.98281
C	3.730311	-0.50305	-1.68584
Zn	3.943813	2.488054	-3.34188
H	6.337127	5.578475	-6.88089
H	7.782144	3.317426	-6.86089
H	0.343507	6.213489	-2.46339
H	2.020966	7.309941	-4.26481
H	0.227712	3.922078	-1.22736
H	7.519726	-1.29325	-4.18737
H	5.805643	-2.33198	-2.38631
H	3.667373	-1.45935	-1.17903

C	-6.86788	4.283589	5.651258
C	-7.77096	5.311051	5.348438
C	-7.20339	3.342341	6.633312
C	-9.00218	5.413581	6.007249
H	-7.49378	6.024678	4.582133
C	-8.42332	3.409714	7.317352
H	-6.48783	2.560479	6.856802
C	-9.29944	4.452215	6.983593
H	-10.2498	4.518012	7.50416
C	-0.9512	8.06135	5.616282
C	0.054056	7.974279	6.588321
C	-1.50723	9.311356	5.314202
C	0.512629	9.112301	7.263048
H	0.465183	6.997317	6.811464
C	-1.07621	10.47429	5.964037
H	-2.27912	9.35674	4.555608
C	-0.06879	10.34402	6.930465
H	0.276132	11.23605	7.443966
C	8.011189	0.900652	-5.70038
C	7.915321	-0.11277	-6.66307
C	9.263894	1.459106	-5.41434
C	9.047192	-0.57723	-7.34408
H	6.936362	-0.52562	-6.87393
C	10.42089	1.022418	-6.07094
H	9.316157	2.237392	-4.66276
C	10.28189	0.006803	-7.02752
H	11.16927	-0.34265	-7.54599
C	4.231313	6.815891	-5.75274
C	5.261414	7.721894	-5.46818
C	3.280423	7.142276	-6.72856
C	5.357144	8.947095	-6.13913
H	5.982579	7.451843	-4.7064
C	3.340755	8.355929	-7.4243
H	2.49663	6.424543	-6.93787
C	4.386217	9.235264	-7.10873
H	4.446648	10.1808	-7.63855
C	-4.23595	-6.92496	-5.62283
C	-3.28661	-7.26879	-6.59415
C	-5.2652	-7.82596	-5.31995
C	-3.34771	-8.49498	-7.26749
H	-2.50345	-6.55474	-6.81792
C	-5.3616	-9.06329	-5.96817
H	-5.98516	-7.54225	-4.56201
C	-4.39224	-9.3688	-6.93402
H	-4.45322	-10.3239	-7.44635
C	-8.0174	-1.01081	-5.67355
C	-9.26931	-1.56499	-5.37599
C	-7.92348	-0.01475	-6.65437
C	-10.4274	-1.1411	-6.03895
H	-9.32005	-2.3297	-4.61051
C	-9.05653	0.436449	-7.34231
H	-6.9451	0.39503	-6.87377
C	-10.2904	-0.14281	-7.01386
H	-11.1786	0.196443	-7.53763
C	0.957558	-7.96187	5.762389
C	1.51748	-9.21975	5.483638
C	-0.04517	-7.86335	6.728959
C	1.089039	-10.3678	6.153364
H	2.289972	-9.27673	4.726453
C	-0.50445	-8.99227	7.427488
H	-0.46358	-6.8841	6.938774
C	0.077138	-10.2255	7.120624
H	-0.26084	-11.1107	7.645388
C	6.872491	-4.18274	5.724481
C	7.207522	-3.22382	6.689442
C	7.776168	-5.21488	5.439956
C	8.427553	-3.27817	7.374441
H	6.491471	-2.43855	6.898979
C	9.007568	-5.30477	6.100292
H	7.499314	-5.94233	4.686647
C	9.30432	-4.32591	7.05926
H	10.25472	-4.38177	7.580833
C	8.976399	-1.6859	-8.41283
C	11.81514	1.610126	-5.77595
C	12.3994	2.225799	-7.06948
H	13.39391	2.644125	-6.87741
H	12.49966	1.483009	-7.86624
H	11.75902	3.031447	-7.44329
C	12.75321	0.486613	-5.27469
H	13.75093	0.888156	-5.06422
H	12.36726	0.036684	-4.35421
H	12.86549	-0.31158	-6.01427
C	11.77093	2.711625	-4.7
H	11.14249	3.554738	-5.00454
H	11.39484	2.333214	-3.74408
H	12.78055	3.098155	-4.52733
C	9.49154	-1.13419	-9.76331
H	10.52701	-0.78805	-9.69454
H	9.451212	-1.91259	-10.5336
H	8.879529	-0.29123	-10.1003

C	9.859398	-2.8792	-7.97703
H	9.511956	-3.29641	-7.02619
H	9.823831	-3.67482	-8.72978
H	10.90626	-2.58809	-7.85053
C	7.541599	-2.20508	-8.62437
H	7.124252	-2.6366	-7.70891
H	6.867466	-1.41342	-8.96672
H	7.543039	-2.98971	-9.38783
C	2.315158	8.743719	-8.50786
C	3.045078	8.962692	-9.85427
H	2.329126	9.243322	-10.635
H	3.79342	9.757895	-9.78824
H	3.556227	8.049154	-10.175
C	1.598884	10.0515	-8.09523
H	1.065313	9.923235	-7.14775
H	2.301489	10.88092	-7.9724
H	0.8691	10.34379	-8.8587
C	1.243474	7.65693	-8.71607
H	1.682121	6.708425	-9.04212
H	0.667653	7.471013	-7.80375
H	0.539509	7.977985	-9.49059
C	6.473158	9.969365	-5.84628
C	5.842689	11.29886	-5.36825
H	6.624077	12.0383	-5.15937
H	5.172966	11.72651	-6.1201
H	5.263055	11.14933	-4.45155
C	7.28994	10.2244	-7.13524
H	6.665756	10.61682	-7.94334
H	8.08528	10.95386	-6.9448
H	7.75586	9.300185	-7.4925
C	7.443622	9.480644	-4.75436
H	6.93284	9.310175	-3.80123
H	7.948093	8.552638	-5.04219
H	8.216493	10.23699	-4.5836
C	1.61215	9.051323	8.341804
C	-1.66147	11.86572	5.651488
C	-2.28824	12.46152	6.93437
H	-1.55233	12.56901	7.736553
H	-2.70493	13.45423	6.729798
H	-3.09707	11.82445	7.306983
C	-0.5338	12.79943	5.151398
H	-0.93365	13.7951	4.928409
H	0.257992	12.9186	5.896749
H	-0.07593	12.40521	4.238366
C	-2.75372	11.81163	4.566589
H	-2.36713	11.42688	3.617415
H	-3.59939	11.1859	4.869612
H	-3.13878	12.8196	4.381444
C	1.04878	9.578204	9.682911
H	1.820583	9.544997	10.46019
H	0.702934	10.61289	9.601997
H	0.203146	8.968895	10.01806
C	2.808911	9.930832	7.908427
H	3.234297	9.57516	6.96428
H	2.518622	10.97644	7.770228
H	3.598083	9.902162	8.668217
C	2.129872	7.61862	8.570459
H	1.335484	6.947278	8.911974
H	2.569316	7.193336	7.662446
H	2.90794	7.627072	9.34056
C	-1.60972	-8.83068	8.489387
C	1.676683	-11.7639	5.868244
C	-1.98739	-10.1671	9.155464
H	-2.77349	-9.99845	9.898457
H	-2.3709	-10.8915	8.429417
H	-1.13597	-10.6198	9.674195
C	-2.88001	-8.2497	7.823964
H	-2.69048	-7.27557	7.364125
H	-3.25447	-8.92076	7.043792
H	-3.6741	-8.11798	8.567581
C	-1.12342	-7.86349	9.594783
H	-0.2274	-8.25302	10.089
H	-0.88011	-6.87541	9.19405
H	-1.90124	-7.7322	10.3555
C	0.551055	-12.7091	5.385148
H	0.952981	-13.7082	5.181931
H	-0.24139	-12.8153	6.131722
H	0.093761	-12.3334	4.464038
C	2.303283	-12.334	7.162944
H	1.567017	-12.4272	7.966565
H	2.721534	-13.3299	6.977695
H	3.111021	-11.6887	7.523601
C	2.76986	-11.7291	4.78356
H	2.383497	-11.3633	3.82681
H	3.614159	-11.0961	5.075256
H	3.156805	-12.7398	4.618231
C	10.02747	-6.42287	5.807115
C	8.824882	-2.24377	8.446111
C	9.056492	-2.96283	9.796247
H	9.851313	-3.71146	9.728878

H	9.344045	-2.24057	10.56864
H	8.146124	-3.47165	10.12943
C	7.73962	-1.17081	8.655709
H	7.545043	-0.60244	7.740528
H	6.794355	-1.60714	8.994046
H	8.067528	-0.46051	9.421516
C	10.12853	-1.5304	8.015769
H	10.42748	-0.79439	8.770631
H	10.95709	-2.23367	7.891011
H	9.991375	-1.00451	7.065243
C	9.52908	-7.40225	4.7276
H	8.603982	-7.90471	5.028029
H	9.349611	-6.89922	3.772004
H	10.28414	-8.17622	4.556083
C	10.29453	-7.22913	7.100256
H	11.02241	-8.02586	6.909574
H	10.6942	-6.59834	7.899626
H	9.373757	-7.69231	7.469762
C	11.35234	-5.79597	5.311872
H	11.78665	-5.12011	6.054346
H	12.09007	-6.57886	5.102544
H	11.19426	-5.22379	4.391927
C	-2.32396	-8.90211	-8.34569
C	-3.05631	-9.1459	-9.6865
H	-2.34171	-9.44053	-10.4633
H	-3.80425	-9.94	-9.60454
H	-3.56838	-8.23854	-10.0229
C	-1.60638	-10.2019	-7.91061
H	-1.07114	-10.0561	-6.9666
H	-2.30839	-11.0292	-7.77141
H	-0.87786	-10.5077	-8.66995
C	-1.25312	-7.8189	-8.57574
H	-1.6928	-6.87666	-8.91818
H	-0.67562	-7.61616	-7.66808
H	-0.55052	-8.15375	-9.34563
C	-6.47667	-10.0805	-5.65466
C	-7.29561	-10.3594	-6.93729
H	-6.67269	-10.7664	-7.73913
H	-8.09034	-11.0855	-6.73208
H	-7.7625	-9.44207	-7.31065
C	-5.84479	-11.4007	-5.15348
H	-6.6255	-12.1365	-4.92971
H	-5.17622	-11.8418	-5.89856
H	-5.2636	-11.2342	-4.2407
C	-7.44541	-9.57219	-4.57017

H	-6.93302	-9.38407	-3.62123
H	-7.95077	-8.64981	-4.87407
H	-8.21766	-10.3256	-4.3842
C	-11.8208	-1.72463	-5.73181
C	-12.406	-2.36396	-7.01338
H	-13.4	-2.77955	-6.81271
H	-12.5077	-1.63571	-7.82328
H	-11.7654	-3.17571	-7.37334
C	-11.7743	-2.80661	-4.63632
H	-11.1456	-3.65454	-4.92648
H	-11.3973	-2.4108	-3.68783
H	-12.7834	-3.19082	-4.45546
C	-12.7594	-0.59318	-5.24965
H	-12.8734	0.19143	-6.00335
H	-13.7564	-0.99185	-5.03062
H	-12.3726	-0.12634	-4.33796
C	-8.9879	1.525587	-8.43109
C	-9.50328	0.948673	-9.77091
H	-10.5383	0.602544	-9.69507
H	-9.46448	1.712925	-10.5554
H	-8.89044	0.100457	-10.0928
C	-7.5539	2.042621	-8.6531
H	-7.1364	2.49111	-7.74589
H	-6.87905	1.24574	-8.98162
H	-7.55689	2.81333	-9.4306
C	-9.8721	2.725533	-8.01649
H	-9.83803	3.507343	-8.78364
H	-10.9185	2.435488	-7.884
H	-9.52454	3.160423	-7.07366
C	-10.0215	6.526828	5.694157
C	-8.82126	2.394883	8.407343
C	-10.1254	1.674844	7.989879
H	-9.98872	1.132043	7.048844
H	-10.4248	0.952591	8.75774
H	-10.9535	2.376352	7.852695
C	-9.05227	3.138048	9.744464
H	-9.8466	3.88589	9.663822
H	-9.34025	2.429866	10.52963
H	-8.14154	3.652112	10.06846
C	-7.73672	1.325085	8.635982
H	-7.54265	0.740292	7.731096
H	-6.79111	1.766737	8.966352
H	-8.06503	0.628828	9.414412
C	-9.52245	7.48662	4.597485
H	-8.59697	7.993628	4.888951

H	-9.3434	6.966648	3.650921
H	-10.277	8.257975	4.412331
C	-11.3466	5.89188	5.209958
H	-11.7813	5.229504	5.964261
H	-12.0839	6.671274	4.986672
H	-11.1887	5.303379	4.300337
C	-10.2883	7.356079	6.972729
H	-11.0158	8.149614	6.767813
H	-10.6884	6.73981	7.783159
H	-9.36737	7.82534	7.334049

Cartesian coordinates of optimized geometry of **CP4** (xyz format; number of atoms: 412)

MPPP conformer, E(RB3LYP) = -15429.9115004 a.u.

C	-6.75887	-1.83035	-3.2268
C	-6.03223	-0.93666	-2.46723
C	-4.74493	-1.54347	-2.22421
N	-4.67502	-2.78194	-2.83444
C	-5.91945	-2.98262	-3.45338
C	-4.79518	-7.27459	-5.16304
C	-5.87676	-6.448	-5.14243
C	-5.50311	-5.2451	-4.39009
N	-4.14596	-5.39752	-3.94856
C	-3.72173	-6.60024	-4.41777
C	-6.30188	-4.1468	-4.16892
C	0.66523	-6.17357	-2.62264
C	-0.02493	-7.05844	-3.36551
C	-1.40827	-6.51907	-3.55524
N	-1.4955	-5.26478	-2.89359
C	-0.2534	-5.03526	-2.30122
C	-2.40374	-7.15168	-4.23973
C	-1.44456	-0.89577	-0.27503
C	-0.39284	-1.76664	-0.29981
C	-0.76354	-2.90473	-1.18523
N	-2.03634	-2.74801	-1.63958
C	-2.51788	-1.4895	-1.13104
C	-3.72102	-0.94027	-1.42597
C	0.104664	-3.96845	-1.51399
Zn	-3.0896	-4.03853	-2.83287
H	-7.76095	-1.72345	-3.5954
H	-6.33556	0.025517	-2.10805
H	-4.68636	-8.24031	-5.61926
H	-6.84783	-6.59417	-5.58071
H	1.684072	-6.20808	-2.28354
H	0.286703	-7.99993	-3.7852
H	-3.9724	0.050467	-1.01779
H	1.128497	-3.91102	-1.13697
C	0.857976	-1.61743	0.394556
C	1.662798	-0.47618	0.379827
C	2.815452	-0.78443	1.21026
N	2.725103	-2.06882	1.698592
C	1.511297	-2.59613	1.222996
C	1.998396	-6.80463	3.377298
C	1.058712	-6.14474	2.649138
C	1.598796	-4.81574	2.327157
N	2.904723	-4.70854	2.922071
C	3.148221	-5.89427	3.533324
C	0.984902	-3.86612	1.564129
C	7.478992	-4.64791	5.056856
C	6.686284	-5.7339	5.092987
C	5.415872	-5.39725	4.377665
N	5.506605	-4.0578	3.905902
C	6.744892	-3.57074	4.317509
C	4.358795	-6.24554	4.22864
C	6.09708	0.781861	2.535097
C	7.033211	0.101885	3.231218
C	6.527053	-1.28721	3.427817
N	5.298452	-1.42394	2.85531
C	4.976051	-0.15203	2.252654
C	3.874134	0.134957	1.525513
C	7.245778	-2.29821	4.111637
Zn	4.099871	-3.05841	2.846467
H	1.96924	-7.79687	3.788814
H	0.088823	-6.48344	2.334096
H	-0.01867	-4.09212	1.164941
H	8.466461	-4.49796	5.459593
H	6.858505	-6.70138	5.533746
H	6.101265	1.805446	2.20458
H	7.985827	0.431147	3.608413
H	3.753647	1.155639	1.132768
C	1.013479	6.856424	0.096022
C	1.78253	7.765816	-0.5297
C	2.735845	7.028842	-1.42048
N	2.503446	5.64231	-1.275

C	1.432614	5.498887	-0.38094
C	1.463198	0.796225	-0.26604
C	0.471992	1.762775	-0.13564
C	1.153551	3.046429	-0.4911
N	2.319031	2.808086	-1.15343
C	2.558417	1.397939	-1.06996
C	0.811649	4.337569	-0.00959
C	6.29179	1.445543	-3.98276
C	5.567337	0.543477	-3.22681
C	4.566767	1.298794	-2.51712
N	4.686901	2.651537	-2.82221
C	5.741934	2.753413	-3.72885
C	3.584308	0.725717	-1.65863
C	5.5209	7.496986	-4.0083
C	6.214823	6.491651	-4.60023
C	5.716505	5.217938	-4.05047
N	4.658183	5.522015	-3.12214
C	4.55627	6.874625	-3.07779
C	3.660299	7.618756	-2.23834
C	6.207208	3.969765	-4.32088
Zn	3.537491	4.147137	-2.10699
H	0.21771	6.999564	0.806014
H	1.777122	8.840578	-0.45955
H	0.053262	4.390263	0.77801
H	7.111953	1.244514	-4.64411
H	5.696556	-0.5176	-3.16542
H	3.674069	-0.3574	-1.48015
H	5.605221	8.559096	-4.14662
H	6.998756	6.547233	-5.33513
C	-7.72311	4.474661	4.709324
C	-7.37958	5.644486	4.101037
C	-6.29915	5.354284	3.152915
N	-5.98718	3.956641	3.253055
C	-6.85988	3.425795	4.148318
C	-5.50968	-1.06363	3.316314
C	-6.41197	-0.42311	4.083061
C	-6.25184	1.047498	3.853631
N	-5.20714	1.236922	2.907101
C	-4.71338	-0.02621	2.585829
C	-6.98682	2.023304	4.45905
C	-0.9056	1.566711	0.225432
C	-1.60474	0.383035	0.372334
C	-2.82078	0.680576	1.188893
N	-2.97158	2.022835	1.327635
C	-1.91671	2.633507	0.551422
C	-3.64421	-0.30884	1.768447
C	-4.09558	6.842945	0.404329
C	-3.07728	6.16595	-0.23843
C	-3.05781	4.828115	0.302034
N	-4.06438	4.675081	1.232331
C	-4.70501	5.923559	1.332972
C	-5.73678	6.236143	2.256132
C	-2.05277	3.85293	-0.01953
Zn	-4.54241	2.983532	2.203591
H	-8.47303	4.291807	5.455333
H	-7.79424	6.626243	4.244478
H	-5.32903	-2.11664	3.202679
H	-7.15115	-0.82102	4.757527
H	-3.38986	-1.35179	1.571507
H	-4.39905	7.862857	0.266768
H	-2.41341	6.53446	-0.99229
H	-1.3398	4.176887	-0.79327
C	-2.14286	-8.48056	-4.85143
C	-2.48117	-9.64393	-4.14741
C	-1.56078	-8.55758	-6.12285
C	-2.24628	-10.9004	-4.71889
H	-2.92717	-9.54935	-3.15886
C	-1.30043	-9.80791	-6.69892
H	-1.32364	-7.63577	-6.65171
C	-1.65263	-10.9657	-5.98921
H	-1.46177	-11.9419	-6.43643
C	-7.69029	-4.17039	-4.71715
C	-7.959	-3.5551	-5.94576
C	-8.70504	-4.80645	-3.99349
C	-9.26167	-3.56532	-6.45992
H	-7.14446	-3.07227	-6.48471
C	-10.0094	-4.84225	-4.50479
H	-8.46717	-5.26275	-3.03312
C	-10.271	-4.21533	-5.73202
H	-11.2864	-4.22996	-6.13097
C	-6.24429	7.639309	2.241793
C	-5.8533	8.530877	3.245024
C	-7.10871	8.045847	1.215772
C	-6.3358	9.84877	3.237182
H	-5.16952	8.194631	4.024422
C	-7.58275	9.36182	1.184055
H	-7.39624	7.323342	0.452484
C	-7.18894	10.2487	2.200985
H	-7.55645	11.27556	2.175531
C	-8.01182	1.652006	5.46861

C	-9.31874	1.359094	5.055355
C	-7.66144	1.59976	6.822833
C	-10.292	1.020171	6.002533
H	-9.5569	1.405563	3.994025
C	-8.61946	1.23998	7.780665
H	-6.64195	1.846252	7.116113
C	-9.92688	0.961011	7.357363
H	-10.6813	0.694012	8.09822
C	8.598122	-1.96189	4.62178
C	8.743409	-1.42393	5.907523
C	9.718368	-2.18457	3.8106
C	10.0163	-1.09335	6.38876
H	7.85487	-1.26986	6.516874
C	11.00027	-1.87966	4.286422
H	9.57363	-2.59202	2.81155
C	11.13206	-1.33104	5.57089
H	12.12626	-1.08172	5.943517
C	4.431901	-7.61744	4.795446
C	4.965141	-8.65656	4.022592
C	3.965826	-7.85653	6.094439
C	5.047102	-9.95076	4.552292
H	5.306729	-8.44125	3.011517
C	4.02196	-9.14947	6.6301
H	3.562022	-7.0262	6.671317
C	4.568683	-10.1812	5.851129
H	4.621485	-11.1887	6.265997
C	7.326064	3.848624	-5.30004
C	8.648156	3.801434	-4.83555
C	7.04445	3.783771	-6.66664
C	9.702938	3.692218	-5.74765
H	8.832252	3.841991	-3.76286
C	8.093571	3.673435	-7.5947
H	6.008745	3.811291	-7.00532
C	9.409233	3.61848	-7.12108
H	10.23335	3.51068	-7.82745
C	3.773565	9.098878	-2.26748
C	2.857481	9.853152	-3.01305
C	4.79321	9.727821	-1.5416
C	2.948232	11.25042	-3.02633
H	2.08579	9.33334	-3.57772
C	4.908587	11.12401	-1.56046
H	5.485988	9.116144	-0.96595
C	3.977479	11.86947	-2.29877
H	4.053902	12.9573	-2.30776
C	-5.88872	10.84336	4.314022

C	-8.53794	9.854112	0.091054
C	-9.92995	10.03701	0.728736
H	-10.6619	10.38435	-0.00702
H	-9.91014	10.7669	1.544183
H	-10.3004	9.093673	1.145077
C	-8.03149	11.19909	-0.46951
H	-8.6627	11.54812	-1.29298
H	-7.00945	11.10587	-0.85279
H	-8.02759	11.9857	0.291713
C	-8.64829	8.858822	-1.07911
H	-9.05561	7.893844	-0.75804
H	-7.67299	8.673477	-1.543
H	-9.31244	9.241597	-1.86131
C	-6.96541	11.91852	4.561442
H	-7.14611	12.5291	3.670595
H	-6.66505	12.60054	5.363471
H	-7.91826	11.46473	4.854146
C	-4.59345	11.51114	3.811245
H	-3.80367	10.77109	3.640609
H	-4.21345	12.23971	4.533486
H	-4.75637	12.0354	2.863824
C	-5.61946	10.12691	5.651696
H	-4.79233	9.412533	5.574816
H	-6.5022	9.575173	5.992238
H	-5.35317	10.84147	6.437169
C	-8.27377	1.198181	9.273145
C	-8.73693	2.532737	9.891034
H	-8.52133	2.57208	10.96286
H	-9.81395	2.68199	9.762605
H	-8.23231	3.383507	9.419776
C	-8.99696	0.022675	9.961264
H	-8.74575	-0.92954	9.481995
H	-10.0856	0.134564	9.925287
H	-8.71439	-0.05276	11.01607
C	-6.76063	1.024221	9.504043
H	-6.18666	1.868118	9.105308
H	-6.38662	0.112355	9.025854
H	-6.52864	0.954796	10.57169
C	-11.7314	0.68178	5.599188
C	-11.9173	-0.8418	5.750029
H	-12.9314	-1.14783	5.475822
H	-11.7393	-1.17113	6.778619
H	-11.2201	-1.39033	5.107357
C	-12.7182	1.428151	6.520661
H	-12.6253	1.109552	7.563807

H	-13.755	1.250725	6.218336
H	-12.5437	2.509205	6.490015
C	-12.0391	1.084359	4.144613
H	-11.4081	0.545012	3.429772
H	-11.8847	2.157259	3.984723
H	-13.0793	0.862133	3.885151
C	-9.60228	-2.91846	-7.80702
C	-11.1515	-5.50511	-3.72701
C	-12.0944	-6.25226	-4.69202
H	-12.5872	-5.57058	-5.39302
H	-12.8834	-6.77875	-4.14559
H	-11.5473	-6.99556	-5.28177
C	-11.9231	-4.38685	-2.99843
H	-12.7586	-4.79072	-2.41888
H	-12.331	-3.65442	-3.70266
H	-11.2715	-3.84228	-2.30569
C	-10.627	-6.52111	-2.69448
H	-10.007	-6.04216	-1.92835
H	-10.0247	-7.30143	-3.17203
H	-11.4528	-7.01677	-2.17354
C	-9.78988	-4.05122	-8.83637
H	-10.0341	-3.65272	-9.82587
H	-10.5959	-4.73248	-8.54597
H	-8.87777	-4.64977	-8.93655
C	-10.9033	-2.09981	-7.67934
H	-10.8174	-1.34502	-6.88985
H	-11.7642	-2.73173	-7.43925
H	-11.1356	-1.57691	-8.61259
C	-8.48899	-1.97238	-8.29376
H	-7.54672	-2.5036	-8.46658
H	-8.2949	-1.17446	-7.56805
H	-8.7637	-1.49261	-9.23909
C	7.769998	3.565586	-9.08897
C	11.16223	3.608085	-5.28634
C	8.985712	3.920849	-9.96713
H	8.728478	3.876037	-11.0308
H	9.819535	3.228803	-9.80989
H	9.344605	4.934635	-9.75957
C	7.346436	2.109351	-9.36515
H	6.469394	1.826814	-8.77182
H	8.147561	1.406471	-9.11311
H	7.09244	1.960218	-10.419
C	6.621099	4.525906	-9.45656
H	6.864075	5.558418	-9.18424
H	5.687396	4.261807	-8.94747
H	6.415922	4.506274	-10.5317
C	11.61273	2.140142	-5.4259
H	12.65182	2.010252	-5.10835
H	11.53687	1.790112	-6.46036
H	10.99328	1.476403	-4.81234
C	12.04321	4.517492	-6.16709
H	12.0519	4.192147	-7.21245
H	13.08158	4.519342	-5.82058
H	11.68511	5.552496	-6.14705
C	11.33309	4.052419	-3.82143
H	10.77933	3.405879	-3.13168
H	10.98503	5.08002	-3.671
H	12.38487	4.016351	-3.51876
C	5.994137	11.84735	-0.75604
C	1.976306	12.11871	-3.8331
C	2.742598	12.68309	-5.0473
H	3.598241	13.2927	-4.7391
H	2.096897	13.31065	-5.66911
H	3.128996	11.87592	-5.67989
C	0.758801	11.3192	-4.33534
H	0.198592	10.8758	-3.50463
H	1.053823	10.50904	-5.01157
H	0.066169	11.96199	-4.88899
C	1.459993	13.27368	-2.94959
H	0.711417	13.8726	-3.47817
H	2.265606	13.95241	-2.64992
H	0.993737	12.89148	-2.03437
C	7.159598	10.90872	-0.38967
H	7.622642	10.47937	-1.28521
H	6.83123	10.07891	0.24631
H	7.940594	11.44486	0.159748
C	6.568892	13.02303	-1.57239
H	7.387186	13.51473	-1.03721
H	5.811881	13.78703	-1.77811
H	6.961601	12.67919	-2.53541
C	5.338727	12.37498	0.536288
H	4.519302	13.06761	0.318026
H	6.062375	12.90493	1.162636
H	4.922155	11.55539	1.132037
C	10.22211	-0.51849	7.794642
C	10.75888	-1.65447	8.688976
H	10.93204	-1.30734	9.712135
H	11.70565	-2.0527	8.310067
H	10.0504	-2.4885	8.737162
C	11.23981	0.639631	7.746028

H	10.91609	1.419503	7.04817	H	4.508905	-9.85347	9.976588
H	12.2306	0.301166	7.425657	H	5.394767	-8.71763	8.953049
H	11.35913	1.104207	8.729969	C	2.662784	-8.32544	8.623574
C	8.912031	0.023913	8.396396	H	1.7838	-8.14337	7.99539
H	8.161417	-0.76441	8.518571	H	3.214809	-7.38321	8.708687
H	8.476073	0.806359	7.765625	H	2.300508	-8.57589	9.626348
H	9.083283	0.459837	9.386263	C	2.70552	-10.7535	8.053851
C	12.24631	-2.08826	3.418832	H	2.300937	-10.9635	9.049114
C	13.40424	-2.64347	4.27301	H	3.300056	-11.6233	7.756332
H	13.71303	-1.93814	5.051464	H	1.859817	-10.6797	7.361587
H	14.28536	-2.8519	3.658037	C	-2.58692	-12.2001	-3.98161
H	13.11674	-3.57718	4.768081	C	-0.6878	-9.93368	-8.09791
C	12.63672	-0.71692	2.83184	C	0.353744	-11.0711	-8.1223
H	13.52741	-0.79251	2.200997	H	1.137762	-10.9034	-7.37682
H	12.84965	0.012217	3.620279	H	0.837703	-11.1441	-9.10113
H	11.82891	-0.3051	2.216872	H	-0.09925	-12.0449	-7.91204
C	11.98685	-3.0798	2.268893	C	-1.83498	-10.2495	-9.07833
H	11.22017	-2.71207	1.578018	H	-2.34714	-11.1796	-8.81241
H	11.65637	-4.05291	2.647653	H	-1.46599	-10.3549	-10.1029
H	12.89461	-3.24752	1.679988	H	-2.58703	-9.4526	-9.07686
C	5.604014	-11.121	3.734199	C	0.015473	-8.63597	-8.53799
C	6.52228	-11.9918	4.615879	H	0.807468	-8.35251	-7.83673
H	6.976545	-12.8016	4.036034	H	-0.68448	-7.79597	-8.6084
H	5.975778	-12.4547	5.444129	H	0.476846	-8.75187	-9.52419
H	7.334617	-11.3972	5.047477	C	-3.52305	-11.9598	-2.78233
C	6.423974	-10.6407	2.521922	H	-4.46023	-11.4862	-3.09535
H	7.262559	-10.0075	2.831074	H	-3.05993	-11.3174	-2.02549
H	5.812533	-10.0669	1.8169	H	-3.78202	-12.9024	-2.28882
H	6.841133	-11.488	1.967402	C	-1.26503	-12.8126	-3.47609
C	4.404928	-11.9537	3.238221	H	-0.57856	-13.0278	-4.3012
H	3.808465	-12.3385	4.071856	H	-1.4404	-13.7495	-2.93878
H	4.734221	-12.8113	2.643694	H	-0.74888	-12.1294	-2.79282
H	3.737383	-11.3524	2.611054	C	-3.28774	-13.1798	-4.94515
C	3.539086	-9.45579	8.052121	H	-3.59477	-14.094	-4.42751
C	4.786594	-9.62859	8.942114	H	-2.63495	-13.4788	-5.77154
H	5.426284	-10.443	8.587227	H	-4.18529	-12.728	-5.3815

Cartesian coordinates of optimized geometry of **CP4** (xyz format; number of atoms: 412)

PPPP conformer, E(RB3LYP) = -15429.9115004 a.u.

C	5.734014	-4.80929	2.49839	C	4.860062	-5.69176	1.701582
C	5.269006	-3.54038	2.373876	C	3.413147	-9.65565	-0.46872
C	4.074104	-3.59586	1.50416	C	4.409868	-9.24732	0.39855
N	3.825404	-4.87322	1.118246	C	4.192397	-7.84994	0.665045

N	3.067015	-7.40698	-0.02759
C	2.580928	-8.51008	-0.72614
C	5.020678	-7.03364	1.503651
C	-1.24779	-6.21602	-2.37999
C	-0.67922	-7.44022	-2.51962
C	0.567345	-7.45348	-1.72957
N	0.722182	-6.14856	-1.13403
C	-0.35872	-5.41762	-1.50825
C	1.409877	-8.5141	-1.55434
C	1.318675	-1.27099	0.079601
C	-0.04118	-1.83265	-0.06912
C	0.216217	-3.2898	-0.40552
N	1.507033	-3.65178	-0.00288
C	2.165351	-2.48643	0.408738
C	3.32229	-2.44453	1.131018
C	-0.63744	-4.07413	-1.12581
Zn	2.28836	-5.53793	-0.013
H	6.582274	-5.17136	3.053099
H	5.649694	-2.63179	2.798637
H	3.259648	-10.6319	-0.88454
H	5.206591	-9.8343	0.811138
H	-2.16178	-5.83898	-2.79641
H	-1.025	-8.29023	-3.08211
H	3.733393	-1.4831	1.45061
H	-1.60946	-3.68214	-1.43728
C	-1.27171	-1.32091	0.085849
C	-1.83526	0.037524	-0.07036
C	-3.29166	-0.22547	-0.40542
N	-3.65229	-1.51205	0.013171
C	-2.48534	-2.16587	0.426007
C	-4.79631	-5.72067	2.551624
C	-3.52809	-5.25688	2.416804
C	-3.58806	-4.06803	1.538947
N	-4.8675	-3.82247	1.157474
C	-5.68326	-4.85192	1.753789
C	-2.43912	-3.31805	1.155943
C	-9.65764	-3.42263	-0.40863
C	-9.24568	-4.41075	0.466658
C	-7.84748	-4.18991	0.726267
N	-7.40812	-3.06989	0.023144
C	-8.51413	-2.59082	-0.67621
C	-7.02663	-5.01267	1.566233
C	-6.22196	1.210139	-2.39452
C	-7.44684	0.63997	-2.52234
C	-7.45623	-0.59758	-1.71843
N	-6.14906	-0.74547	-1.12662
C	-5.42026	0.331852	-1.51511
C	-4.07671	0.617518	-1.13693
C	-8.51905	-1.43269	-1.52177
Zn	-5.53757	-2.29374	0.018845
H	-5.15531	-6.56503	3.114284
H	-2.61714	-5.63458	2.83922
H	-1.4757	-3.72589	1.473667
H	-10.6346	-3.27607	-0.82526
H	-9.83074	-5.2042	0.888174
H	-5.84709	2.119653	-2.82249
H	-8.29996	0.97997	-3.08357
H	-3.68616	1.586289	-1.46016
C	1.241541	6.213437	-2.38126
C	0.676355	7.439747	-2.51605
C	-0.56757	7.455033	-1.72187
N	-0.72564	6.148528	-1.13069
C	0.352702	5.415744	-1.50874
C	-1.3261	1.269772	0.078673
C	0.033981	1.831042	-0.06964
C	-0.2229	3.28822	-0.40595
N	-1.51313	3.650964	-0.00183
C	-2.17277	2.485531	0.407009
C	0.630206	4.071513	-1.12785
C	-5.74831	4.807095	2.486692
C	-5.28425	3.538063	2.360429
C	-4.0847	3.594854	1.497167
N	-3.83287	4.873127	1.116091
C	-4.86841	5.691424	1.698333
C	-3.33207	2.443696	1.12563
C	-3.39987	9.663478	-0.44236
C	-4.39937	9.254148	0.421192
C	-4.19015	7.853545	0.676954
N	-3.06567	7.41005	-0.01678
C	-2.57404	8.515259	-0.70814
C	-1.40459	8.518743	-1.53864
C	-5.02482	7.034934	1.507177
Zn	-2.29137	5.538932	-0.00817
H	2.152969	5.834544	-2.80159
H	1.022974	8.289799	-3.07797
H	1.601117	3.678343	-1.4413
H	-6.59978	5.167924	3.037369
H	-5.66857	2.628416	2.779678
H	-3.74449	1.482216	1.443462
H	-3.24046	10.64221	-0.84995

H	-5.19223	9.843095	0.838396
C	9.650972	3.42015	-0.41158
C	9.23925	4.408773	0.463368
C	7.841092	4.188278	0.72335
N	7.401389	3.06821	0.02062
C	8.507313	2.588479	-0.67869
C	6.21531	-1.21551	-2.39057
C	7.440021	-0.64524	-2.51957
C	7.449276	0.593545	-1.71759
N	6.142224	0.742021	-1.12562
C	5.41358	-0.33596	-1.51247
C	8.511945	1.429324	-1.52278
C	1.264652	1.319602	0.085543
C	1.828103	-0.03889	-0.06967
C	3.284723	0.223405	-0.40422
N	3.645295	1.510552	0.01276
C	2.478378	2.164841	0.424837
C	4.069934	-0.62089	-1.13404
C	4.790126	5.720433	2.548279
C	3.521787	5.257032	2.413531
C	3.581484	4.067693	1.536186
N	4.860889	3.82156	1.15494
C	5.676985	4.851049	1.750846
C	7.020428	5.011219	1.563525
C	2.432375	3.317706	1.153792
Zn	5.530812	2.292522	0.016613
H	10.62789	3.273095	-0.8282
H	9.824484	5.20232	0.884426
H	5.840499	-2.12573	-2.81707
H	8.29311	-0.98592	-3.08048
H	3.679414	-1.59013	-1.45591
H	5.14943	6.564939	3.11054
H	2.610892	5.635311	2.835619
H	1.469067	3.725917	1.471312
C	1.097201	-9.79266	-2.25567
C	1.7885	-10.1201	-3.42916
C	0.119115	-10.6471	-1.73419
C	1.503988	-11.3191	-4.09387
H	2.545001	-9.43177	-3.80364
C	-0.18558	-11.8447	-2.39524
H	-0.38879	-10.3714	-0.81078
C	0.516524	-12.167	-3.56598
H	0.292139	-13.103	-4.07936
C	6.151596	-7.72054	2.192294
C	5.986864	-8.16726	3.508127

C	7.362448	-7.90808	1.51408
C	7.048344	-8.80207	4.167337
H	5.027144	-8.02097	4.002354
C	8.425811	-8.5573	2.153305
H	7.455805	-7.54639	0.490968
C	8.254556	-8.99276	3.477129
H	9.08135	-9.49567	3.980928
C	7.703242	6.136501	2.265228
C	7.943072	7.334137	1.580112
C	8.092392	5.980365	3.600617
C	8.585392	8.393687	2.2327
H	7.626537	7.41978	0.54142
C	8.722156	7.037095	4.271557
H	7.90514	5.030345	4.099406
C	8.963381	8.231009	3.575114
H	9.460463	9.054575	4.08963
C	9.793013	1.118387	-2.22084
C	10.80094	0.42515	-1.53902
C	9.968387	1.521008	-3.54966
C	12.0063	0.131266	-2.18834
H	10.6296	0.127312	-0.50539
C	11.16266	1.219345	-4.21796
H	9.171253	2.072912	-4.04614
C	12.16894	0.530025	-3.52477
H	13.10447	0.299914	-4.03661
C	-9.80065	-1.1232	-2.21927
C	-9.9734	-1.51962	-3.55037
C	-10.8124	-0.43864	-1.53464
C	-11.1688	-1.22068	-4.21768
H	-9.17351	-2.06507	-4.04963
C	-12.0196	-0.14872	-2.18277
H	-10.6436	-0.14373	-0.49964
C	-12.1793	-0.54079	-3.52132
H	-13.1157	-0.31267	-4.03264
C	-7.70902	-6.13872	2.267046
C	-8.09822	-5.98398	3.602947
C	-7.94843	-7.33558	1.581326
C	-8.72629	-7.04173	4.273098
H	-7.91127	-5.03411	4.101958
C	-8.59152	-8.39593	2.232894
H	-7.63118	-7.42271	0.542783
C	-8.96655	-8.23585	3.575775
H	-9.46005	-9.06115	4.09118
C	-6.15753	7.720463	2.19377
C	-7.33475	7.994059	1.480691

C	-6.03473	8.081504	3.536753
C	-8.4001	8.636563	2.117308
H	-7.39208	7.702147	0.43307
C	-7.1002	8.723405	4.192479
H	-5.10845	7.869077	4.069953
C	-8.26674	8.996879	3.47193
H	-9.09839	9.505862	3.959108
C	-1.08773	9.799554	-2.23375
C	-0.08256	10.63179	-1.72748
C	-1.80257	10.15219	-3.38542
C	0.222433	11.8337	-2.37993
H	0.44397	10.3356	-0.8211
C	-1.50953	11.3503	-4.04812
H	-2.58578	9.485359	-3.74364
C	-0.5017	12.17996	-3.53057
H	-0.27708	13.11988	-4.03643
C	8.86118	9.729833	1.534402
C	9.185898	6.906029	5.726418
C	10.71742	6.729413	5.713939
H	11.11478	6.62472	6.72806
H	11.21923	7.583628	5.248255
H	11.00778	5.83551	5.150277
C	8.807069	8.177079	6.51372
H	9.077218	8.083572	7.5705
H	7.729303	8.365828	6.462839
H	9.316427	9.065807	6.127464
C	8.543337	5.697978	6.433257
H	8.824209	4.750814	5.959514
H	7.449979	5.764738	6.428068
H	8.861489	5.638364	7.479535
C	10.32836	10.14198	1.773099
H	10.53856	10.31646	2.833093
H	10.57345	11.06478	1.237908
H	11.01706	9.364211	1.424816
C	7.91034	10.78387	2.137026
H	6.862238	10.49963	1.991989
H	8.05566	11.76309	1.67064
H	8.068473	10.90494	3.213428
C	8.62625	9.653624	0.014161
H	7.584371	9.41612	-0.22658
H	9.260894	8.891862	-0.45257
H	8.857216	10.60889	-0.46891
C	11.40722	1.659974	-5.66544
C	12.34993	2.879362	-5.62727
H	12.56421	3.249165	-6.63458

H	13.3063	2.636149	-5.15323
H	11.90666	3.704355	-5.05865
C	12.06047	0.512343	-6.46233
H	11.44391	-0.39211	-6.42871
H	13.04952	0.252639	-6.07123
H	12.19042	0.785057	-7.51463
C	10.09964	2.051588	-6.37949
H	9.613614	2.908635	-5.90075
H	9.384017	1.222577	-6.38892
H	10.28774	2.332252	-7.42132
C	13.13629	-0.63544	-1.49293
C	13.19523	-2.04922	-2.10578
H	13.98209	-2.65114	-1.64109
H	13.39645	-2.01719	-3.18149
H	12.24636	-2.5799	-1.96763
C	14.47409	0.097407	-1.72232
H	14.75004	0.126355	-2.78148
H	15.29261	-0.39447	-1.18747
H	14.42137	1.132755	-1.36729
C	12.91084	-0.75594	0.025724
H	11.99971	-1.31764	0.259849
H	12.82929	0.229253	0.498913
H	13.74311	-1.27965	0.507663
C	6.901415	-9.32824	5.599322
C	9.775235	-8.77628	1.460675
C	10.23603	-10.2329	1.673109
H	10.41266	-10.4576	2.729879
H	11.16944	-10.4356	1.138565
H	9.484068	-10.9409	1.307063
C	10.79175	-7.80271	2.090674
H	11.78019	-7.90917	1.633413
H	10.90534	-7.97507	3.165714
H	10.47615	-6.76174	1.959337
C	9.700958	-8.51454	-0.05508
H	9.43329	-7.47589	-0.27771
H	8.961211	-9.16282	-0.53828
H	10.66575	-8.70696	-0.53609
C	6.670542	-10.8502	5.511777
H	6.553742	-11.2936	6.505219
H	7.506033	-11.3572	5.018384
H	5.766243	-11.0811	4.937481
C	8.182232	-9.03484	6.4072
H	8.410335	-7.96358	6.409976
H	9.053331	-9.55739	5.998732
H	8.074395	-9.35277	7.4492

C	5.715624	-8.67442	6.333856
H	4.759154	-8.90364	5.851065
H	5.819086	-7.58464	6.371
H	5.645635	-9.0317	7.366682
C	-6.93132	9.13467	5.659115
C	-9.70911	8.965714	1.390918
C	-8.27266	9.512312	6.316607
H	-8.13204	9.775159	7.370632
H	-8.98429	8.680426	6.280348
H	-8.73631	10.37696	5.830001
C	-6.31833	7.967898	6.46011
H	-5.31024	7.716284	6.112435
H	-6.93057	7.063624	6.374375
H	-6.23848	8.214886	7.523547
C	-5.98857	10.35407	5.696783
H	-6.40054	11.19278	5.124893
H	-5.00834	10.12023	5.266639
H	-5.82345	10.69937	6.721798
C	-10.8671	8.248145	2.114375
H	-11.8219	8.428138	1.610508
H	-10.9757	8.586541	3.149603
H	-10.7048	7.164873	2.140145
C	-9.92288	10.49287	1.429039
H	-10.0204	10.86777	2.452651
H	-10.8299	10.78065	0.888408
H	-9.08051	11.01999	0.967127
C	-9.69638	8.511537	-0.0807
H	-9.56157	7.428025	-0.16965
H	-8.89582	8.999253	-0.64811
H	-10.6403	8.760568	-0.57732
C	-2.26924	11.79453	-5.30281
C	1.292092	12.79154	-1.84422
C	0.564849	13.96534	-1.15826
H	-0.0859	14.50122	-1.85667
H	1.275175	14.68864	-0.7467
H	-0.06481	13.61267	-0.33337
C	2.223489	12.108	-0.82555
H	2.728715	11.24012	-1.26309
H	1.678094	11.76676	0.061474
H	3.000304	12.79697	-0.47762
C	2.159264	13.3151	-3.00751
H	2.9666	13.95754	-2.64191
H	1.575031	13.90509	-3.72112
H	2.618114	12.48833	-3.56046
C	-3.21084	10.69927	-5.83675
H	-3.98197	10.4336	-5.10513
H	-2.66238	9.786851	-6.0951
H	-3.7294	11.03267	-6.74196
C	-3.10709	13.03712	-4.93955
H	-3.68892	13.38891	-5.79697
H	-2.47899	13.86864	-4.60516
H	-3.81134	12.81492	-4.1298
C	-1.25648	12.14304	-6.41282
H	-0.61474	12.98456	-6.13365
H	-1.76551	12.41741	-7.34216
H	-0.60368	11.2908	-6.63173
C	-11.4093	-1.65639	-5.66709
C	-12.3185	-2.90114	-5.63299
H	-12.5294	-3.26854	-6.6418
H	-13.278	-2.6871	-5.1508
H	-11.8492	-3.71881	-5.07368
C	-12.098	-0.52185	-6.45294
H	-11.5055	0.398915	-6.4169
H	-13.0917	-0.29165	-6.05447
H	-12.2271	-0.79154	-7.50604
C	-10.0949	-2.00647	-6.39004
H	-9.58512	-2.85625	-5.92241
H	-9.40059	-1.15904	-6.39227
H	-10.2797	-2.28019	-7.43412
C	-13.1505	0.612069	-1.48216
C	-14.4986	-0.0822	-1.76458
H	-14.7549	-0.06343	-2.82882
H	-15.3167	0.40633	-1.22599
H	-14.4743	-1.1314	-1.4495
C	-13.168	2.048121	-2.0436
H	-13.9573	2.647013	-1.57897
H	-13.3392	2.057848	-3.12482
H	-12.2147	2.556258	-1.86017
C	-12.9534	0.669237	0.044155
H	-12.0399	1.208119	0.318601
H	-12.8932	-0.3352	0.478417
H	-13.7879	1.184924	0.530756
C	-9.18805	-6.91497	5.729015
C	-10.7228	-6.76758	5.722018
H	-11.118	-6.66588	6.737298
H	-11.2106	-7.63312	5.262514
H	-11.0323	-5.88195	5.155547
C	-8.56705	-5.69298	6.4312
H	-8.86619	-4.75281	5.954804
H	-7.47268	-5.73937	6.42522

H	-8.88501	-5.63597	7.477701
C	-8.78159	-8.17707	6.517152
H	-9.27442	-9.07632	6.134027
H	-9.04992	-8.08719	7.574694
H	-7.70051	-8.34466	6.462906
C	-8.85795	-9.73103	1.528942
C	-10.2854	-10.2183	1.851
H	-10.4172	-10.4261	2.917698
H	-10.5211	-11.1404	1.310217
H	-11.0311	-9.46784	1.566457
C	-8.72809	-9.61447	-0.0013
H	-7.71383	-9.33455	-0.30578
H	-9.41745	-8.86469	-0.40557
H	-8.9582	-10.5666	-0.49074
C	-7.82297	-10.7484	2.050005
H	-7.95974	-11.729	1.584125
H	-7.8996	-10.8835	3.133615
H	-6.80073	-10.4187	1.834636
C	2.223044	-11.7272	-5.38444
C	-1.2297	-12.8217	-1.84364
C	-2.09173	-13.3809	-2.99369
H	-2.57435	-12.5731	-3.5541
H	-2.88089	-14.0376	-2.61368
H	-1.49807	-13.9663	-3.70352
C	-0.46961	-13.9688	-1.14863
H	0.191825	-14.4944	-1.84509
H	-1.15913	-14.7058	-0.72602
H	0.154524	-13.5918	-0.33061
C	-2.17123	-12.1469	-0.82817
H	-2.69492	-11.2938	-1.27312
H	-1.62976	-11.7851	0.053012
H	-2.93275	-12.8476	-0.46978
C	3.373386	-10.7685	-5.74417
H	4.127601	-10.7279	-4.95032
H	3.01384	-9.74891	-5.92005
H	3.883229	-11.0907	-6.65847
C	1.19239	-11.7186	-6.53186
H	0.376498	-12.4257	-6.35259
H	1.657115	-11.9885	-7.48528
H	0.743303	-10.7265	-6.65169
C	2.810842	-13.1425	-5.20891
H	3.377304	-13.4495	-6.09377
H	2.031631	-13.8934	-5.04536
H	3.490376	-13.1825	-4.3503
